# Salt Cluster With Surface Defect Shows Anomalous Acid–Base Chemistry

**DOI:** 10.1002/anie.202520403

**Published:** 2026-01-07

**Authors:** Jessica C. Hartmann, Jia Yang Lim, Yiqi Sheng, Marc Reimann, Sarah J. Madlener, Christian van der Linde, Chi‐Kit Siu, Martin K. Beyer

**Affiliations:** ^1^ Institut für Ionenphysik und Angewandte Physik Universität Innsbruck Technikerstraße 25 Innsbruck 6020 Austria; ^2^ Department of Chemistry City University Hong Kong Tat Chee Avenue Kowloon Hong Kong SAR P.R. China

**Keywords:** Density functional theory, Ion‐molecule reaction, Metadynamics, Proton transfer, Salt cluster

## Abstract

The acid displacement reaction of formic acid in sea‐salt aerosols to release hydrogen chloride is an enigma in atmospheric chemistry: a weak acid transfers a proton to the conjugate base of a strong acid, reversing solution‐phase acid–base chemistry. To shed light on this question, we compared the reactivity of formic acid with Na_13_Cl_12_^+^ and Na_14_Cl_13_^+^ under ultra‐high vacuum conditions in a mass spectrometer. No reaction was observed with Na_14_Cl_13_^+^, a known magic cluster that corresponds to a cubic section of the crystal lattice. Na_13_Cl_12_^+^, which corresponds to the cubic section with a surface defect, reacts readily with release of hydrogen chloride and incorporation of formate into the salt structure. Quantum chemical calculations show that the reaction is substantially endothermic for the magic cluster. The surface defect, however, makes it exothermic. The reason lies in the induced fit of the formate ion in the Na_13_Cl_11_(HCOO)^+^ cluster. Its oxygen atoms interact with five sodium ions. In sum, these electrostatic interactions more than offset the 49 ± 13 kJ mol^−1^ difference in gas‐phase acidity between hydrogen chloride and formic acid.

## Graphical abstract

The acid displacement of formic acid in sea‐salt aerosols, releasing HCl, challenges conventional acid–base chemistry. Our gas‐phase experiments show that this reaction occurs on pure NaCl cluster ions in the absence of water. Surface defects of the NaCl cluster ions enable this reaction via enhanced electrostatic interactions of the salt ions with the formate, outweighing the gas‐phase acidity difference between HCl and formic acid.

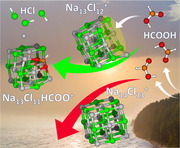

Brønsted–Lowry acid–base chemistry involving a strong and a weak acid in aqueous solution knows only one direction: the strong acid transfers a proton to the conjugate base of the weak acid. This concept also holds in the acid displacement reaction of gaseous nitric acid with solid sodium chloride samples, where Beichert and Finlayson‐Pitts observed the release of hydrogen chloride in Knudsen cell studies.^[^ ^1^ ^]^ Single‐particle analysis by Gard and coworkers confirmed that the reaction takes place in the atmosphere on the surface of sea‐salt aerosols.^[^ ^2^ ^]^ In a later combined field and laboratory study, Laskin et al. observed that weak organic acids RCOOH also react via the acid displacement reaction (1).^[^ ^3^ ^]^ They assumed that the reaction proceeds in a concentrated aqueous solution while the particles are drying, and release of HCl to the gas phase shifts the equilibrium to the product side of reaction (1). Su et al. confirmed this observation.^[^ ^4^, ^5^ ^]^

(1) $${\text{NaCl}}_{\left(\text{aq}\right)}+{\text{RCOOH}}_{\text{(aq,g)}}\to {\text{RCOONa}}_{\text{(aq)}}+{\text{HCl}}_{\text{(aq,g)}}$$

Sea‐salt aerosols are a complex mixture of a wide variety of ions, organic compounds, and water,^[^ ^6^, ^7^, ^8^, ^9^ ^]^ which makes it virtually impossible to obtain a molecular‐level understanding of the reaction pathway. Laboratory studies of macroscopic samples^[^ ^1^ ^]^ or artificial aerosols^[^ ^3^ ^]^ ensure a defined composition, but the role of water remains an open question. Reactivity studies of gas‐phase cluster ions in the ultra‐high vacuum of a mass spectrometer provide control over the size and composition of the reactant ion.^[^ ^10^, ^11^, ^12^ ^]^ Size‐selected clusters often have a unique structure,^[^ ^13^ ^]^ suggesting that the surface chemistry of small particles can be studied with a variety of structural motifs. Numerous studies of transition metal clusters have shown that their reactivity is often strongly size dependent.^[^ ^14^, ^15^, ^16^ ^]^ We have previously used salt cluster ions to probe the influence of the ionic environment on the chemistry and photochemistry of organic dopants.^[^ ^17^, ^18^, ^19^, ^20^ ^]^ Here we employ this concept with cationic sodium chloride clusters, with the known magic cluster size Na_14_Cl_13_^+^.^[^ ^21^ ^]^ This cluster of 27 atoms has a cubic 3 × 3 × 3 structure and resembles a section of the crystal lattice. Removal of a NaCl unit from an edge of the cube creates the Na_13_Cl_12_^+^ cluster, which thus features a surface defect. These two clusters serve as model systems for the much more extended surface of pristine, anhydrous sodium chloride crystals.

We generate these two model clusters by electrospray ionization from a sodium chloride solution, using isotopically enriched Na^35^Cl,^[^ ^22^ ^]^ and study their reaction with gaseous formic acid in the cell of a Fourier‐transform ion cyclotron resonance (FT‐ICR) mass spectrometer at a pressure of *P*_ICR_ = 1.4(6) × 10^−7^ mbar (see Supporting Information for further experimental details). Figure 1a shows the relevant section of the mass spectrum of the magic cluster after a reaction time *t* = 20 s. The peak at *m*/*z* 776.45 contains contributions of Na_14_Cl_13_^+^ and Na_28_Cl_26_^2+^, as evidenced by the minor contributions of the ^37^Cl isotope. The Na_28_^35^Cl_25_^37^Cl^2+^ peak (+1 u) is much smaller than the Na_14_^35^Cl_12_^37^Cl^+^ peak (+2 u), which shows that the singly charged magic cluster is dominant. No reaction products are visible at this long reaction delay, showing that both singly and doubly charged clusters are unreactive on the timescale of the experiment. The peak at *m*/*z* 718.49 in Figure 1b contains roughly equal amounts of defect bearing Na_13_Cl_12_^+^ and doubly charged Na_26_Cl_24_^2+^, evidenced by the ≈1:2 ratio of the ^37^Cl containing peaks at *m*/*z* + 2 and +1, respectively. At nominal *t* = 0 s reaction delay, a tiny signal of the Na_13_Cl_11_(HCOO)^+^ product (*x* = 1, reaction (2)) is already visible, since a small delay between ion trapping and the start of the detection cycle is required for technical reasons. After 20 s reaction delay (Figure 1c), Na_13_Cl_12_^+^ is completely converted into products containing up to three formate ions (*x* = 1–3), reaction (2). There is also a tiny signal of the first acid displacement product Na_26_Cl_23_(HCOO)^2+^ of the doubly charged Na_26_Cl_24_^2+^ at *m*/*z* 723.51, but the intensity is so small that a potential second reaction step, that would cause a signal overlapping with the *x* = 1 product of reaction (2), is negligible.

(2) $$\begin{array}{c} {\text{Na}}_{13}{\text{Cl}}_{12}^{+}+x\text{HCOOH}\to {\text{Na}}_{13}{\text{Cl}}_{12-x}{\left(\text{HCOO}\right)}_{x}^{+}+\\ \;x\text{HCl},x=1-3 \end{array}$$

**Figure 1:**
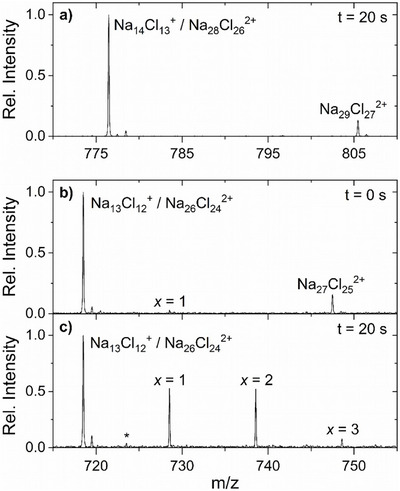
Mass spectra of the reaction of HCOOH with the magic Na_14_Cl_13_^+^ cluster at a) *t* = 20 s and the defect‐bearing Na_13_Cl_12_^+^ cluster at b) *t* = 0 s and c) *t* = 20 s. Doubly charged clusters Na_26_Cl_24_^2+^ and Na_28_Cl_26_^2+^ overlap with the reactant peaks Na_13_Cl_12_^+^ and Na_14_Cl_13_^+^, respectively, but do not interfere with the reaction of the singly charged reactants. Acid displacement reaction (2) is observed for the defect‐bearing Na_13_Cl_12_^+^, resulting in Na_13_Cl_12‐_ *_x_* (HCOO)*_x_* ^+^ (*x* = 1, 2, 3). The tiny peak labeled with an asterisk (*) in c) corresponds to the first acid displacement product Na_26_Cl_23_(HCOO)^2+^ of the doubly charged Na_26_Cl_24_^2+^.

The kinetic fit (Figure 2) confirms this interpretation. Within 20 s experiment time, up to three steps of the acid displacement reaction according to reaction (2) proceed, following pseudo‐first order kinetics. The fit also shows that the first step is relatively fast, and the reaction slows down with each additional step. The contribution of the doubly charged ion reacts very slowly to the product Na_26_Cl_23_(HCOO)^2+^. According to the fit, the ratio of the singly charged Na_13_Cl_12_^+^ and the doubly charged Na_26_Cl_24_^2+^ within the *m*/*z* 718.49 peak in Figure 1b is 1:1, which is consistent with the abundance derived from the intensities of the ^37^Cl isotopologues. The matrix of pseudo‐first order rate coefficients and starting intensities is provided as Table S2. Table 1 provides the experimental bimolecular rate coefficients *k*, which are derived from the pseudo‐first order rate coefficients scaled with the pressure, together with collision rates calculated by the hard‐sphere average dipole orientation (HSA) *k*_HSA_ and surface charge capture (SCC) *k*_SCC_ models for comparison. These models account for the finite size of the cluster.^[^ ^23^ ^]^ While the first step *x* = 1 proceeds with a rate coefficient *k* of 10%–20% collision rate, less than 1 in a 100 collisions is reactive for the subsequent steps. One additional NaCl unit, however, makes the cluster Na_14_Cl_13_^+^ completely inert, with an upper limit for the rate coefficient of *k* < 4.5 × 10^−14^ cm^3^ s^−1^, derived from the detection limit after 20 s exposure to the reactant gas. A comparison with standard average dipole orientation (ADO) theory^[^ ^24^ ^]^ is available in Table S1.

**Figure 2:**
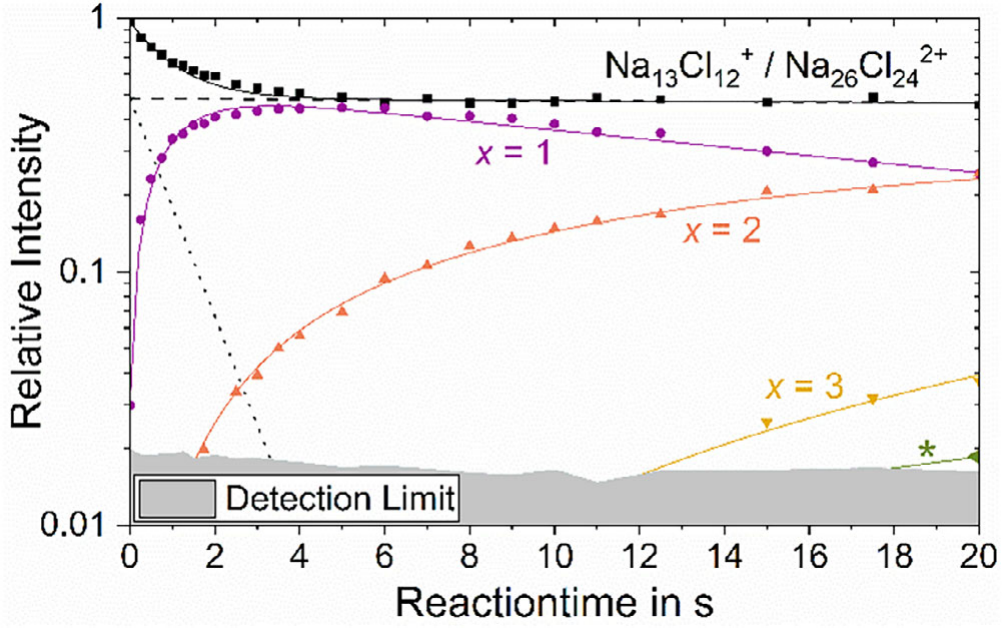
Reaction kinetics of the acid displacement reaction (2) of the defect‐bearing Na_13_Cl_12_^+^ overlapped with the doubly charged Na_26_Cl_24_^2+^. The doubly charged Na_26_Cl_24_^2+^ (dashed black line) reacts very slowly into the product Na_26_Cl_23_(HCOO)^2+^ labeled with an asterisk (*), while the singly charged Na_13_Cl_12_^+^ (dotted black line) is almost completely converted into the products Na_13_Cl_12‐_ *_x_* (HCOO)*_x_* ^+^ (*x* = 1, 2, 3) after a reaction time of 4 s.

**Table 1:** Rate coefficients *k* of reaction (2) compared with HSA and SCC collision rates.^[^ ^23^ ^]^ See Supporting Information for details.

Reaction (2)	*k* in cm^3^ s^−1^	*k*_HSA_ in cm^3^ s^−1^	*k*_SCC_ in cm^3^ s^−1^
*x* = 1	3.0 ± 1.2 × 10^−10^	1.59 × 10^−9^	2.79 × 10^−9^
*x* = 2	1.2 ± 0.5 × 10^−11^	1.59 × 10^−9^	2.80 × 10^−9^
*x* = 3	4.4 ± 1.8 × 10^−12^	1.59 × 10^−9^	2.81 × 10^−9^

To understand the reason for this pronounced cluster‐size dependence, we explored the potential energy surface (PES) for the first acid displacement reaction of the magic cluster Na_14_Cl_13_^+^ and the defect‐bearing Na_13_Cl_12_^+^ (reaction (2), *x* = 1). The minimum energy pathways are shown in Figure 3 (see Supporting Information for additional pathways and further theoretical details). The reaction starts with the uptake of the predominant *trans* rotamer^[^ ^25^ ^]^ of HCOOH to form the adduct **IM*_n_* A**, *n* = 13, 14. Isomerization to *cis*‐HCOOH via **TS*_n_* A** to **IM*_n_* B** aligns the proton toward a chloride ion, which minimizes the rate‐limiting barrier **TS*_n_* B** for the subsequent proton transfer, resulting in the HCl adduct **IM*_n_* C**, with HCOO^−^ embedded in the salt structure. Loss of HCl completes the reaction. It should be noted that once HCl is lost from the cluster, the reaction cannot proceed backward, since the probability that HCl collides with a salt cluster in the mass spectrometer before it is pumped away is vanishingly small.

**Figure 3:**
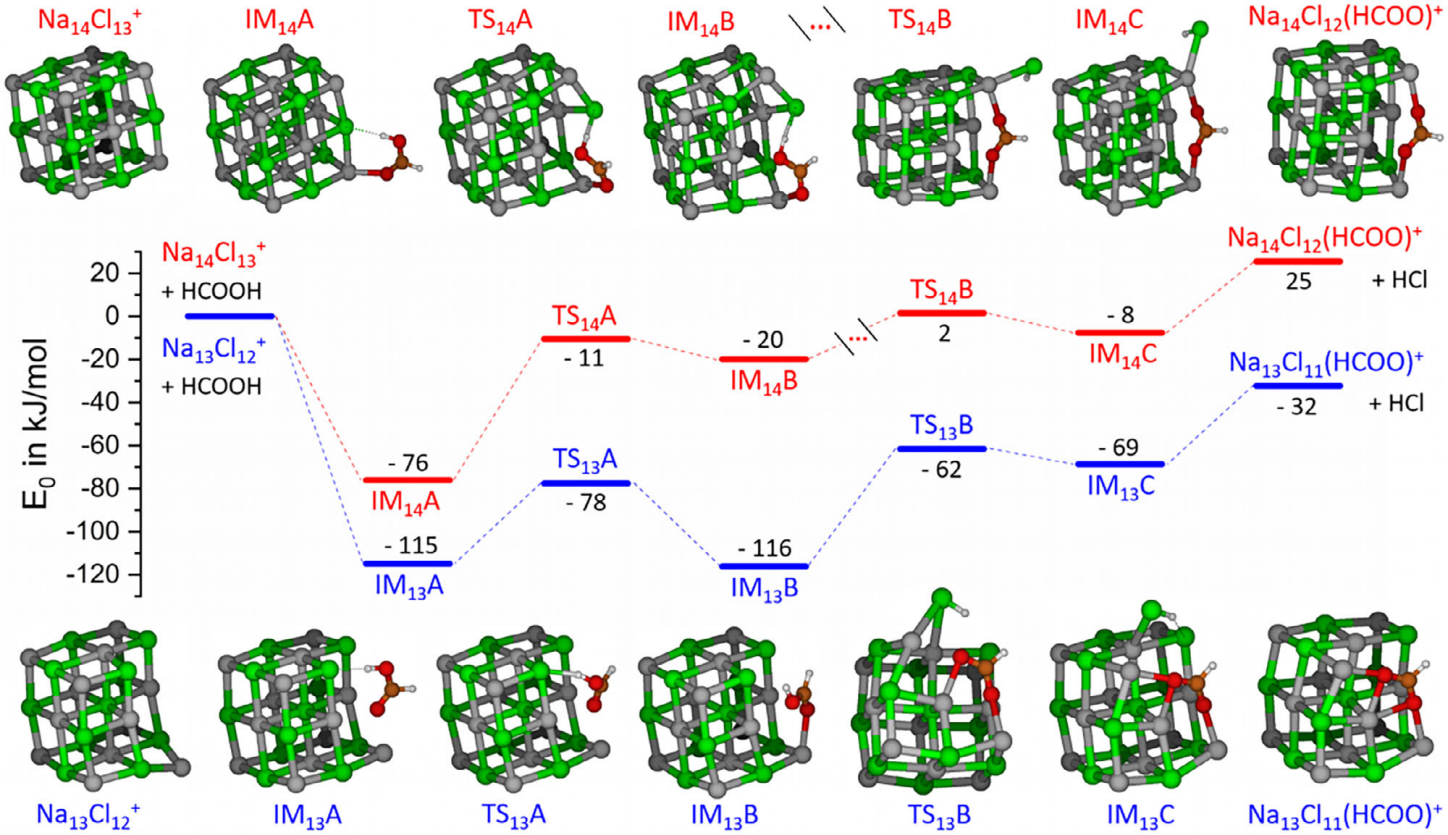
Minimum energy pathways for the reactions Na_14_Cl_13_^+^ + HCOOH → Na_14_Cl_12_(HCOO)^+^ + HCl and Na_13_Cl_12_^+^ + HCOOH → Na_13_Cl_11_(HCOO)^+^ + HCl in red and blue, respectively. Energies are obtained at the RI‐MP2‐F12/cc‐pVDZ‐F12//r^2^SCAN‐3c level of theory.

Throughout the reaction path, relative energies with respect to the entrance channel are significantly lower for the *n* = 13 species. The defect site affords 39 kJ mol^−1^ stronger binding of HCOOH in the collision complex **IM_13_A** than along the edge of Na_14_Cl_13_^+^ in **IM_14_A**. The *cis* rotamer fits even better into the defect site, so that the stronger binding offsets the higher energy, **IM_13_B** is practically isoenergetic to **IM_13_A**. Disrupting the magic cluster structure, as required for the formation of **IM_14_B**, costs additional energy, so reaching this intermediate is much less probable. The penalty paid for disrupting the magic cluster structure cannot be compensated later in the reaction, so that the separated products lie 25 kJ mol^−1^ above the entrance channel in case of the magic cluster *n* = 14, while the reaction is −32 kJ mol^−1^ exothermic in the presence of the defect for *n* = 13, in line with our experimental observations.

A closer look at the structures makes the differences in energy entirely plausible: in the collision complex (**IM_14_A**), the carbonyl oxygen atom of formic acid as well as the acidic proton interact with one Na^+^ and Cl^−^ ion of the magic cluster, respectively, while the surface defect in Na_13_Cl_12_^+^ affords close interaction with three Na^+^ and two Cl^−^ ions (**IM_13_A**). After proton transfer and HCl evaporation, the two oxygen atoms of HCOO^−^ interact with four Na^+^ ions in the former magic cluster, and the hydrogen atom is dangling. The surface defect increases the number of interacting Na^+^ ions to five, with an additional dispersive interaction of the molecule with a newly opened face of the cluster. To illustrate the energy release by replacing Cl^−^ with HCOO^−^, we calculated the energy of reaction (3) from literature values of HCl and HCOOH deprotonation reactions in the gas phase^[^ ^26^ ^]^ and Δ*E*_0_ = −32 kJ mol^−1^ of reaction (2). This shows that HCOO^−^ in Na_13_Cl_11_HCOO^+^ is 85 ± 13 kJ mol^−1^ more strongly bound than the Cl^−^ ion it replaces in Na_13_Cl_12_^+^. See Supporting Information, reactions (S1)–(S4) for complete thermochemical cycle.

(3) $${\text{Na}}_{13}{\text{Cl}}_{12}^{+}+{\text{HCOO}}^{-}\to {\text{Na}}_{13}{\text{Cl}}_{11}{\text{HCOO}}^{+}+{\text{Cl}}^{-}$$

With the substantial structural flexibility of the investigated systems, we found several alternative reaction pathways, which feature at least one higher barrier than the minimum energy paths shown in Figure 3 (Figures S2–S9). Our gas‐phase experiments under ultra‐high vacuum conditions are very well described by such a PES using zero‐point corrected energies. In the atmosphere, however, energy exchange with the environment is rapid, and the situation is better described with free energy surfaces. To this end, we studied the reaction with metadynamics, which also samples a wider part of the conformational space. This enhanced sampling technique coupled with molecular dynamics was employed to explore possible thermodynamic pathways and estimate the free energy surfaces (FES) for the proton transfer process between the reaction complexes **IM_14_A** and **IM_14_C** for the magic cluster and between **IM_13_A** and **IM_13_C** for the defect‐bearing one. Figure 4a shows the resulting FESs along the reaction coordinates defined as coordination number (CN) of O─H and H─Cl interaction, ranging from 0 (without bond) to 1 (with bond) (see Supporting Information for further information); the magic cluster (upper panel) exhibits a significantly higher proton transfer barrier than the defect‐bearing one (lower panel). The minimum free‐energy paths (MFEPs) extracted from the FESs (Figure 4b) show, that the magic cluster requires to overcome a barrier of 67(3) kJ mol^−1^, which is 36(3) kJ mol^−1^ higher than that of the defect‐bearing one. Furthermore, this proton transfer process is endergonic by 30(6) kJ mol^−1^ for the magic cluster, while only 6(5) kJ mol^−1^ for the defect‐bearing cluster. Following the MFEPs, the magic cluster must reduce the Na─Cl coordination number by two to facilitate the proton transfer, while the defect‐bearing cluster only needs to reduce it by one. This lower energetic cost is due to additional stabilization on the hydroxyl oxygen enabled by the defect site and the isomerization of the initially unreactive *trans*‐HCOOH to its *cis* analog (see snapshots in Figure 4c). Our canonical metadynamics assumes a heat bath, which is not present at the ultra‐high vacuum (UHV) conditions of our gas‐phase experiments. Instead, our calculated free energies provide an estimation of the thermodynamic landscape for the reaction in thermally equilibrated environment, such as in the atmosphere. Since the findings of the PESs in Figure 3 and the FESs in Figure 4 are very similar, we think the gas‐phase experiments are a realistic model for more complex environments.

**Figure 4:**
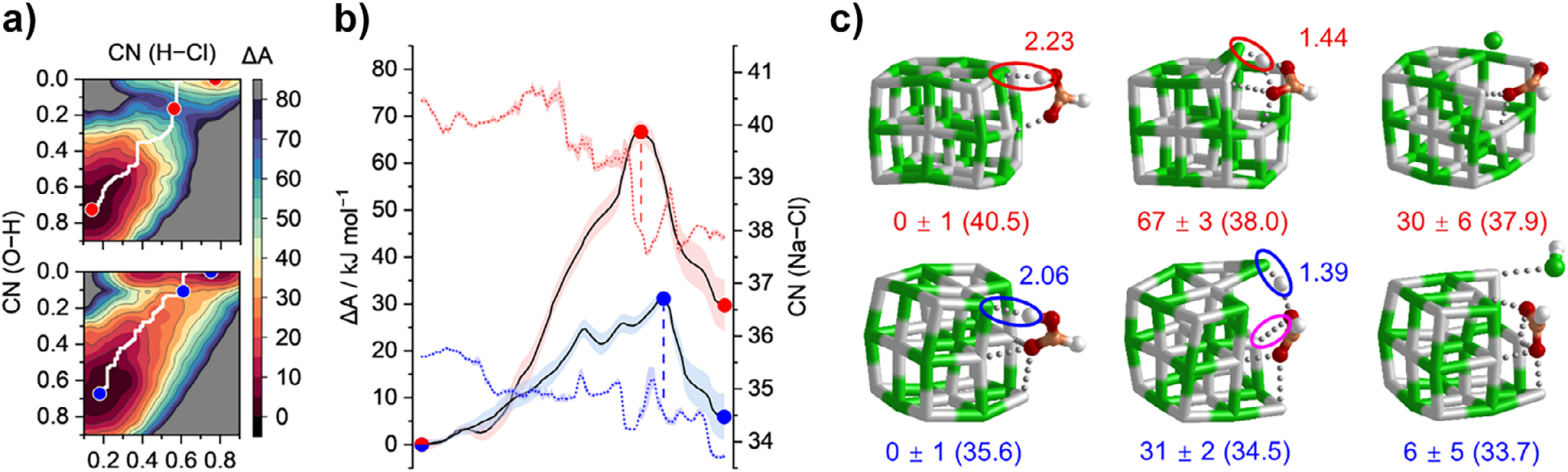
a) Free energy surfaces of the reaction complexes Na_14_Cl_13_(HCOOH)^+^ (upper panel) and Na_13_Cl_12_(HCOOH)^+^ (lower panel). b) Minimum free‐energy paths (MFEPs) extracted from the free energy surfaces. Dotted lines represent coordination number (CN) of Na─Cl with weighted standard error of 0.02−0.31 and 0.01−0.36 for Na_14_Cl_13_(HCOOH)^+^ and Na_13_Cl_12_(HCOOH)^+^, respectively. c) Selected snapshots at points along the MFEPs. Values represent Δ*A* in kJ mol^−1^ and CN(Na─Cl) in parenthesis.

To get an idea whether the relatively small size of the studied clusters has an effect, we performed static calculations similar to Figure 3 for the larger Na_62_Cl_61_^+^ cluster, which we designed to resemble a double step on a (0,0,1) surface of NaCl see Figure (S16). The reaction path we found is very similar to the one for Na_13_Cl_12_^+^ in Figure 3. This indicates that the observed reaction should also be feasible in larger particles, since the stabilizing effect of the defect site on the intermediates and products relies on the interactions of the reactant HCOOH with nearby Na^+^ and Cl^−^ ions and is thus very much localized. At the same time, it does not require the relatively large structural rearrangements observed in the smaller cluster model.

The magic cluster Na_14_Cl_13_^+^, corresponding to a cubic section of the crystal lattice, is non‐reactive, while the defect‐bearing Na_13_Cl_12_^+^ is highly reactive. This is explained by the favorable interactions of the conjugate base of the incoming acid molecule with individual ions in the crystal lattice surrounding the surface defect. These interactions even allow for the formation of the strong acid HCl by proton transfer from the weak acid HCOOH to Cl^−^. This does not make the volatilization‐driven mechanism of HCl elimination from sea‐salt aerosols^[^ ^3^ ^]^ obsolete, but it can rationalize the thermodynamic driving force for deprotonation of the weak acid. The situation in an actual sea‐salt aerosol is certainly much more complex than our ultra‐high vacuum cluster model. Collisions with N_2_ or O_2_ in the atmosphere may stabilize intermediates along the reaction path, and tiny amounts of water may completely alter the mechanism. From a purely mechanistic point of view, however, our study clearly shows that a completely dry salt environment can dramatically change acid‐base chemistry with respect to both gas phase and aqueous solution.

## Supporting Information

The authors have cited additional references within the Supporting Information.^[^ ^23^, ^24^, ^26^, ^27^, ^28^, ^29^, ^30^, ^31^, ^32^, ^33^, ^34^, ^35^, ^36^, ^37^, ^38^, ^39^, ^40^, ^41^, ^42^, ^43^, ^44^, ^45^, ^46^, ^47^, ^48^, ^49^, ^50^ ^]^

## Conflict of Interests

The authors declare no conflict of interest.

## Supporting information



Supporting Information

## Data Availability

The data that support the findings of this study are available in the Supporting Information of this article.
