# Supplementary material for: Salt Cluster With Surface Defect Shows Anomalous Acid–Base Chemistry
Source: Angew Chem Int Ed Engl. 2026 Jan 7;65(7):e20403. doi: 10.1002/anie.202520403 (PMC12887623; doi:10.1002/anie.202520403)
Supplement: Supplementary file 1 — Supporting Information [file ANIE-65-e20403-s001.docx]

Salt Cluster with Surface Defect Shows Anomalous Acid-Base Chemistry

Jessica C. Hartmann,^[a]^ Jia Yang Lim,^[b]^ Yiqi Sheng,^[b]^ Marc Reimann,^[a]^ Sarah J. Madlener,^[a]^ Christian van der Linde,^[a]^ Chi-Kit Siu,*^[b]^ and Martin K. Beyer*^[a]^

Dedicated to Prof. Dr. Dr. h.c. mult. Helmut Schwarz, Wolf Prize Laureate in Chemistry 2025

[a] J. C. Hartmann, M.Sc., Dr. M. Reimann, S. J. Madlener, M.Sc., Priv.-Doz. Dr. C. van der Linde, Prof. Dr. M. K. Beyer
Institut für Ionenphysik und Angewandte Physik
Universität Innsbruck
Technikerstraße 25, 6020 Innsbruck, Austria
E-mail: martin.beyer@uibk.ac.at

[b] J. Y. Lim, B.Sc., Y. Sheng, M.Sc., Prof. Dr. C.-K. Siu
Department of Chemistry
City University Hong Kong
Tat Chee Avenue, Kowloon, Hong Kong SAR, P. R. China
E-mail: chiksiu@cityu.edu.hk

Supporting Information

1. Experimental Details

The experimental setup includes a Bruker Apex Qe 9.4 T Fourier-Transform Ion Cyclotron Resonance Mass Spectrometer (FT-ICR MS) equipped with an Apollo ESI/MALDI Dual Source II and a Nanobay Console.^[1]^ Isotopically enriched Na^35^Cl powder (99 % ^35^Cl, Sigma Aldrich) dissolved in a 1:1 methanol-water mixture (HPLC grade, Carl Roth) at a concentration of 5 mmol/l is used to produce pure sodium chloride cluster ions [NaCl]*_x_*Na^+^ via electrospray ionization (ESI). The ions are transferred into the ICR cell of the FT-ICR MS, where the reaction itself takes place. The reaction gas – formic acid (HCOOH) - is directly dispensed into the ICR cell via a leak valve, increasing the ultra-high vacuum (UHV) pressure from ≈ 10^-10^ mbar to ≈ 10^-7^ mbar. The amount of HCOOH inside the ICR cell is considered as constant throughout our reactivity experiments. Mass spectra of the reaction between HCOOH and the clusters Na_13_Cl_12_^+^ and Na_14_Cl_13_^+^ are recorded for different reaction times. Since no reaction could be observed for the magic cluster Na_14_Cl_13_^+^, only reaction kinetics of the defect-bearing Na_13_Cl_12_^+^ have been recorded. All mass spectra and reaction kinetics shown in this work have been performed using a pressure inside of the ICR cell of 1.4(6) · 10^-7^ mbar.

The reaction kinetics are fitted via a genetic algorithm which provides relative rate coefficients. These relative rate coefficients are transferred into pressure-independent absolute rate coefficients as described in previous studies.^[2]–[5]^ For the reaction gas formic acid a polarizability of α = 3.319 Å^3^ is used for the calculation of the absolute rate coefficients.^[6]^ The ADO, HSA and SCC rates (see **Table S1**) are calculated via the program “HSA collision rate constants vs. 2”, ^[7]^ using a polarizability of α = 3.319 Å^3^,^[6]^ and a dipole moment of µ_D_ = 1.410 D.^[6]^ The locking constant c is taken from a table in ^[8]^, depending on the polarizability and the dipole moment. Screenshots of the input parameters as well as the output rates are shown in **Figures S17 – S19**.

**Table S1.** Rate coefficients *k* of reaction (2) compared with ADO,^[8]^ HSA and SCC collision rates.^[7]^

| Reaction (2) | *k* in cm^3^ s^-1^ | *k*_HSA_ in cm^3^ s^-1^ | *k*_SCC_ in cm^3^ s^-1^ | *k*_ADO_ in cm^3^ s^-1^ |
| --- | --- | --- | --- | --- |
| *x* = 1 | 3.2 ± 1.4 ∙ 10^-10^ | 1.59 ∙ 10^-9^ | 2.79 ∙ 10^-9^ | 1.31 ∙ 10^-9^ |
| *x* = 2 | 1.2 ± 0.5 ∙ 10^-11^ | 1.59 ∙ 10^-9^ | 2.80 ∙ 10^-9^ | 1.31 ∙ 10^-9^ |
| *x* = 3 | 4.4 ± 1.8 ∙ 10^-12^ | 1.59 ∙ 10^-9^ | 2.81 ∙ 10^-9^ | 1.31 ∙ 10^-9^ |

1. Theoretical Details
   1. Potential Energy Surfaces

Quantum chemical calculations have been performed with ORCA 6.1.0^[9],[10]^ in order to explore the potential energy surfaces (PES) of the acid displacement reaction (2) of the clusters Na_2_Cl_3_^+^, Na_13_Cl_12_^+^ and Na_14_Cl_13_^+^ with formic acid. Minimum energy structures of the pure Na_13_Cl_12_^+^ and Na_14_Cl_13_^+^ clusters were verified by global optimization using GOAT^[11]^ at XTB-GFN1^[12]^ level and reoptimization at r^2^SCAN-3c^[13]^ level. The GOAT results are summarized in **Chapter 7** (**Figure S10**). All structures were optimized using r^2^SCAN-3c^[13]^ with improved numerical integration grids (DefGrid3). The resolution of the identity approximation is employed by default, using the respective default basis sets (def2/J). Local Minima and first order transition states were characterized by the appropriate number of real-valued vibrational frequencies. Steepest descent calculations were performed to verify that the transition states connect the correct local minima. Additional single point (SP) calculations were performed using CCSD(T)-F12 and MP2-F12 levels of theory. These calculations employed the cc-pVTZ-F12 and cc-pVDZ-F12 basis sets^[14]^ and the appropriate auxiliary basis sets (denoted as cc-pVXZ-F12-OptRI and cc-pVXZ-F12-MP2Fit in the ORCA library). The RIJCOSX approximation^[15],[16]^ with improved integration grids (DefGrid3) was employed unless explicitly stated.

2.2 Molecular Dynamic Simulations

Density functional theory based molecular dynamics (DFT-MD) were performed using the CP2K version 2024.1 Quickstep module.^[17]^ The Perdew-Burke-Ernzerhof (PBE) functional^[18]^ was employed to evolve the dynamics of a 32 x 32 x 32 Å box with a molecule of formic acid and (NaCl)_x_Na^+^ cluster under open-boundary conditions by the Martyna-Tuckerman Poisson solver.^[19]^ Electronic wavefunctions were described by a double-ζ basis set with single polarization functions with the Goedecker-Teter norm-conserving pseudopotentials and a plane wave cutoff of 300 Ry.^[20]^ Dispersion energies of non-covalent interaction were calculated by using Grimme’s D3 correction with Becke-Johnson damping.^[21]^ A convergence of 10^−5^ Hartree was imposed for the ground-state energy. Atomic positions were propagated using the Born-Oppenheimer MD scheme with a time step of 0.5 fs. The ensemble temperature was controlled at 300 K by the stochastic velocity-rescaling thermostat with a time constant of 50 fs.^[22]^

Free energy surfaces (FES) were calculated at 300K using the PLUMED version 2.9^[23]^ interfaced to the CP2K package. The on-the-fly probability enhanced sampling (OPES)^[24]^ method was employed to accelerate sampling along selected collective variable (CVs) which are functions of atomic coordinates. This history-dependent method builds a bias potential (*V*) to disfavour visited states to sample a targeted Boltzmann distribution $p\left( s \right)$. The unbiased Helmholtz free energy, $F\left( s \right)=-k_{B}T\log\left( p\left( s \right) \right)$ is recovered by reweighting the biased probability distribution.

Three coordination numbers $\left( s \right)$ were chosen as CVs: H−Cl, H−O and Na−Cl defined with respective switching function parameters (r_0) of 1.3 Å, 1.0 Å and 2.7 Å, ($n)$ of 8, $(m)$ of 12 and $\left( d_{0} \right)$ of 0. These inputs tell PLUMED to calculate number of H−Cl, H−O and Na−Cl interactions in the system.

$$s=\frac{1-\left( \frac{r-d_{0}}{r_{0}} \right)^{n}}{1-\left( \frac{r-d_{0}}{r_{0}} \right)^{m}}$$

The OPES bias potential was updated every 100 MD step (PACE=100), utilizing an adaptive bandwidth for the Gaussian kernels. Due to the computational expense of DFT-MD, a multiple-walker strategy was adopted. Preliminary runs with the OPES BARRIER hyperparameter, ranging from 30 to 100 kJ/mol estimated from well-tempered metadynamics DFT-MD,^[25]^ were performed to identify a range that enabled barrier crossing within ~100 ps.

Production runs were performed using BARRIER from 80 to 90 kJ/mol which showed reversible exploration of CVs space under 100 ps. For (NaCl)_12_Na^+^(HCOOH) cluster, independent simulations were initiated from two different input geometries obtained from geometry optimizations, from which 9 trajectories showing reversible crossing were selected for analysis. For (NaCl)_13_Na^+^(HCOOH) cluster, three input geometries were used, yielding 14 productive trajectories. Runs were halted and discarded if significant transient heating caused simulation instability.

To construct the final FES, an ensemble average over these fully independent trajectories was performed. An initial 2 ps equilibration period from each trajectory was discarded before the COLVAR files were concatenated into a single dataset. The final FES and its statistical error were calculated from this combined dataset using the reweighting and block-averaging scheme inherent to the OPES analysis tools. Three projected 2D FES were generated where each surface calculated by integrating out the thermodynamic contribution of the nonchosen CV. Finally, the minimum free-energy path (MFEP) on each 2D FES was identified using the Dijkstra algorithm implemented in MULE version 0.20.^[26]^ The evolution of CV (Na−Cl) along the MFEP was calculated by their weighted average at each point using the concatenated COLVAR file. At each point of MFEP, the CV (Na−Cl) was calculated from an ensemble average of atomic geometries with their respective Boltzmann weight from all trajectories.

3. Reaction Kinetics

The reaction matrix of the reaction kinetics in Figure 2 is shown in **Table S2**. Due to a small delay between ion trapping and the start of the detection cycle, the product peak intensities for a nominal reaction delay *t* = 0 s might be non-zero (Figure 2, *x* = 1). Hence, the initial intensities of the products, obtained via our fitting algorithm, can also be non-zero. The sum of all initial intensities including reactants and products is always 1. The fitted initial intensity of the singly charged Na_13_Cl_12_^+^ is 48.35 % and that of the doubly charged Na_26_Cl_24_^2+^ is 48.22 % and thus, the ratio of singly and doubly charged ions according to the fit is 1:1, which is consistent with the abundance derived from the intensities of the ^37^Cl isotopologues in the experiment.

The relative rate coefficients from the reaction matrix are converted into pressure independent absolute rate coefficients as described in the SI section “1. Experimental Details”. The absolute rate coefficients are listed in **Table S3**.

**Table S2.** Reaction matrix of the kinetic fit, Figure 2. Doubly charged contribution is written in blue, singly charged contribution in green. Reactants are listed in the first line, products in the first column.Non-zero matrix elements are pressure-dependent pseudo-first order rate coefficients in s^-1^.

| Initial Int. | 0.482203 | 0.483518 | 0.000000 | 0.030663 | 0.002898 | 0.000718 |
| --- | --- | --- | --- | --- | --- | --- |
|  | Na_26_Cl_24_^2+^ | Na_13_Cl_12_^+^ | Na_26_Cl_23_(HCOO)^2+^ | Na_13_Cl_11_(HCOO)^+^ | Na_13_Cl_10_(HCOO)_2_^+^ | Na_13_Cl_9_(HCOO)_3_^+^ |
| Na_26_Cl_24_^2+^ | 0.000000 | 0.000000 | 0.000000 | 0.000000 | 0.000000 | 0.000000 |
| Na_13_Cl_12_^+^ | 0.000000 | 0.000000 | 0.000000 | 0.000000 | 0.000000 | 0.000000 |
| Na_26_Cl_23_(HCOO)^2+^ | 0.001971 | 0.000000 | 0.000000 | 0.000000 | 0.000000 | 0.000000 |
| Na_13_Cl_11_(HCOO)^+^ | 0.000000 | 0.98482 | 0.000000 | 0.000000 | 0.000000 | 0.000000 |
| Na_13_Cl_10_(HCOO)_2_^+^ | 0.000000 | 0.000000 | 0.000000 | 0.038754 | 0.000000 | 0.000000 |
| Na_13_Cl_9_(HCOO)_3_^+^ | 0.000000 | 0.000000 | 0.000000 | 0.000000 | 0.014425 | 0.000000 |

**Table S3.** Pressure-independent bimolecular rate coefficients *k* for the reactions Na_13_Cl_12_^+^ + *x* HCOOH → Na_13_Cl_12‑_*_x_*(HCOO)*_x_*^+^ + *x* HCl and Na_26_Cl_24_^2+^ + *x* HCOOH → Na_26_Cl_24‑_*_x_*(HCOO)*_x_*^2+^ + *x* HCl.

| Na_13_Cl_12_^+^ | *k* in cm^3^ s^-1^ |  | Na_26_Cl_24_^2+^ | *k* in cm^3^ s^-1^ |
| --- | --- | --- | --- | --- |
| *x* = 1 | 3.0 ± 1.2 ∙ 10^-10^ |  | *x* = 1 | 6.0 ± 2.5 ∙ 10^-13^ |
| *x* = 2 | 1.2 ± 0.5 ∙ 10^-11^ |  |  |  |
| *x* = 3 | 4.4 ± 1.8 ∙ 10^-12^ |  |  |  |

4. Benchmarking

To decide on a computational protocol usable for the larger cluster sizes Na_13_Cl_12_^+^ and Na_14_Cl_13_^+^, we benchmarked the reaction Na_3_Cl_2_^+^ + HCOOH → Na_3_Cl(HCOO)^+^ + HCl, see **Table S4** and **Figure S1**, using CCSD(T)-F12/cc-pVTZ-F12 energies as reference. All calculations were performed at r^2^SCAN-3c optimized structures and included zero-point vibrational contributions at the same level. RI-MP2-F12 energies are reasonably close to canonical CCSD(T)-F12 energies, improving again on the r^2^SCAN-3c results. Reducing the basis set from TZ to DZ quality greatly speeds up the calculation, while deteriorating the results only by about 1 kJ/mol. The introduction of the COSX-approximation has even small impact on the relative energies. Calculations on the large clusters have therefore been performed at the RI-MP2-F12/cc-pVDZ-F12 level using the RIJCOSX approximation.

**Table S4.** Benchmarking results for the reaction Na_3_Cl_2_^+^ + HCOOH → Na_3_Cl(HCOO)^+^ + HCl based on r^2^SCAN-3c optimized structures. Energies at the respected levels of theories are given in kJ/mol. Mean absolute deviation are calculated with respect to CCSD(T)-F12/cc-pVTZ-F12. All energies include zero-point vibrational contributions at r^2^SCAN-3c level.

| Reaction Path | **CCSD(T)-F12 / cc-pVTZ-F12** | r^2^SCAN-3c | RI-MP2-F12 / cc-pVDZ-F12 (No COSX) | **RI-MP2-F12 / cc-pVDZ-F12** | RI-MP2-F12 / cc-pVTZ-F12 | RI-MP2-F12 / cc-pVTZ-F12 (No COSX) |
| --- | --- | --- | --- | --- | --- | --- |
| Entrance Channel: Na_3_Cl_2_^+^ + HCOOH | **0.0** | 0.0 | 0.0 | **0.0** | 0.0 | 0.0 |
| IM_3_A | **-74.4** | -79.2 | -71.5 | **-71.9** | -72.4 | -72.4 |
| TS_3_A | **-73.0** | -78.1 | -70.1 | **-70.3** | -71.1 | -71.2 |
| IM_3_B | **-85.5** | -90.2 | -83.2 | **-83.6** | -84.0 | -84.1 |
| TS_3_B | **-83.9** | -87.2 | -81.5 | **-81.8** | -82.6 | -82.7 |
| IM_3_C | **-93.7** | -98.1 | -91.9 | **-92.1** | -92.9 | -93.0 |
| TS_3_C | **-56.0** | -55.3 | -53.0 | **-53.1** | -54.5 | -54.6 |
| IM_3_D | **-51.3** | -58.7 | -51.5 | **-51.6** | -53.3 | -53.4 |
| TS_3_D | **-38.9** | -34.7 | -34.3 | **-34.4** | -35.8 | -36.1 |
| IM_3_E | **-43.8** | -42.4 | -40.4 | **-40.5** | -41.9 | -42.3 |
| Exit Channel: Na_3_Cl(HCOO)^+^ + HCl | **-12.9** | -8.5 | -8.2 | **-9.0** | -10.0 | -10.3 |
| Mean Absolute Deviation | **−** | 4.0 | 2.8 | **2.6** | 1.9 | 1.8 |


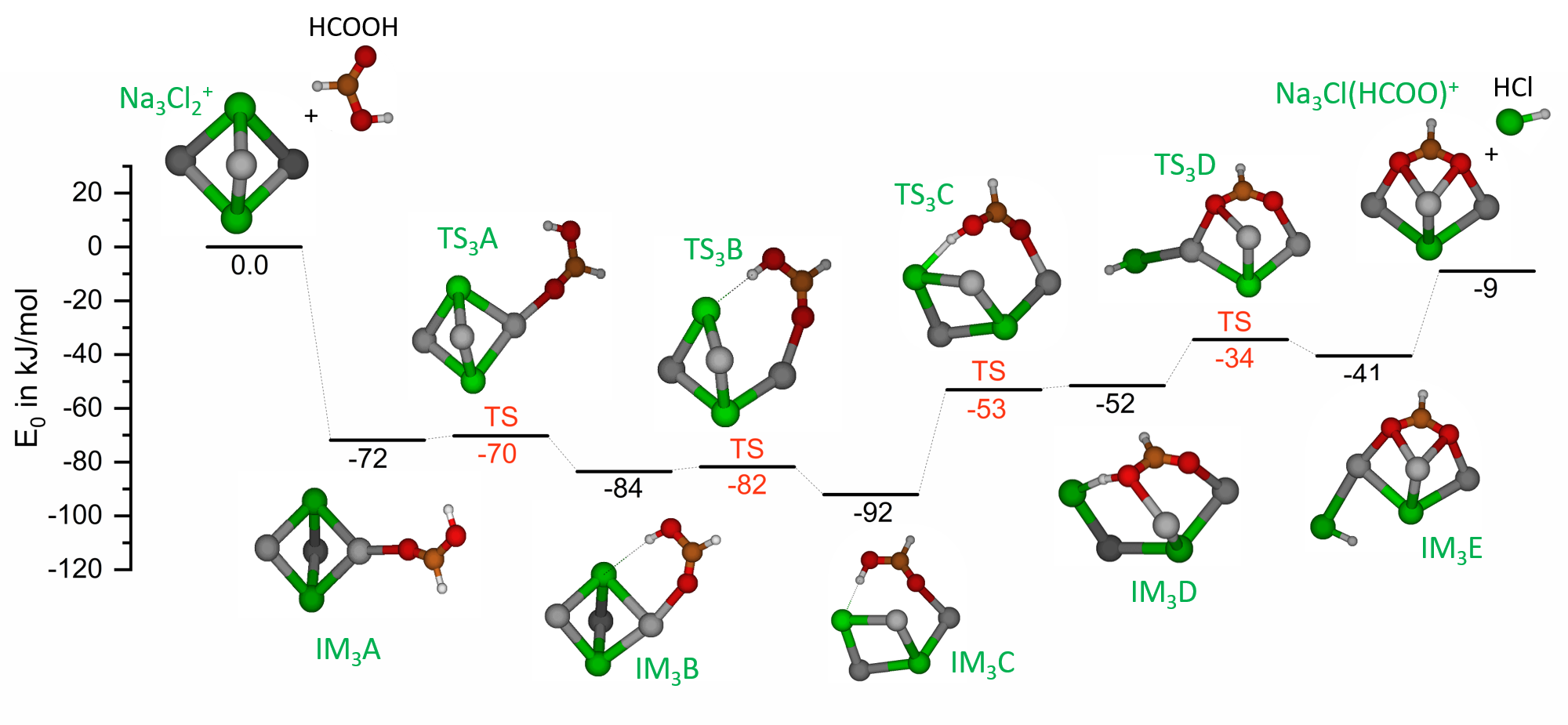


**Figure S1.** Potential energy surface for the reaction Na_3_Cl_2_^+^ + HCOOH → Na_3_Cl(HCOO)^+^ + HCl. Energies are obtained at the RI-MP2-F12/cc-pVDZ-F12//r^2^SCAN-3c level.

5. Potential Energy Surfaces

Several reaction pathways for reactions Na_13_Cl_12_^+^ + HCOOH → Na_13_Cl_11_(HCOO)^+^ + HCl and Na_14_Cl_13_^+^ + HCOOH → Na_14_Cl_12_(HCOO)^+^ + HCl were calculated in order to facilitate the minimum energy pathway for each reaction. The surfaces were obtained using the computational protocol outlined above, and are shown in **Figures S2 – S4** and **Figures S5 – S9** for the clusters Na_13_Cl_12_^+^ and Na_14_Cl_13_^+^, respectively. Minimum energy pathways are illustrated in **Figure S2** and **Figure S5** for the clusters Na_13_Cl_12_^+^ and Na_14_Cl_13_^+^, respectively. Minor rearrangements are labelled with IM_x_Y′ or TS_x_Y′ and framed with a box.


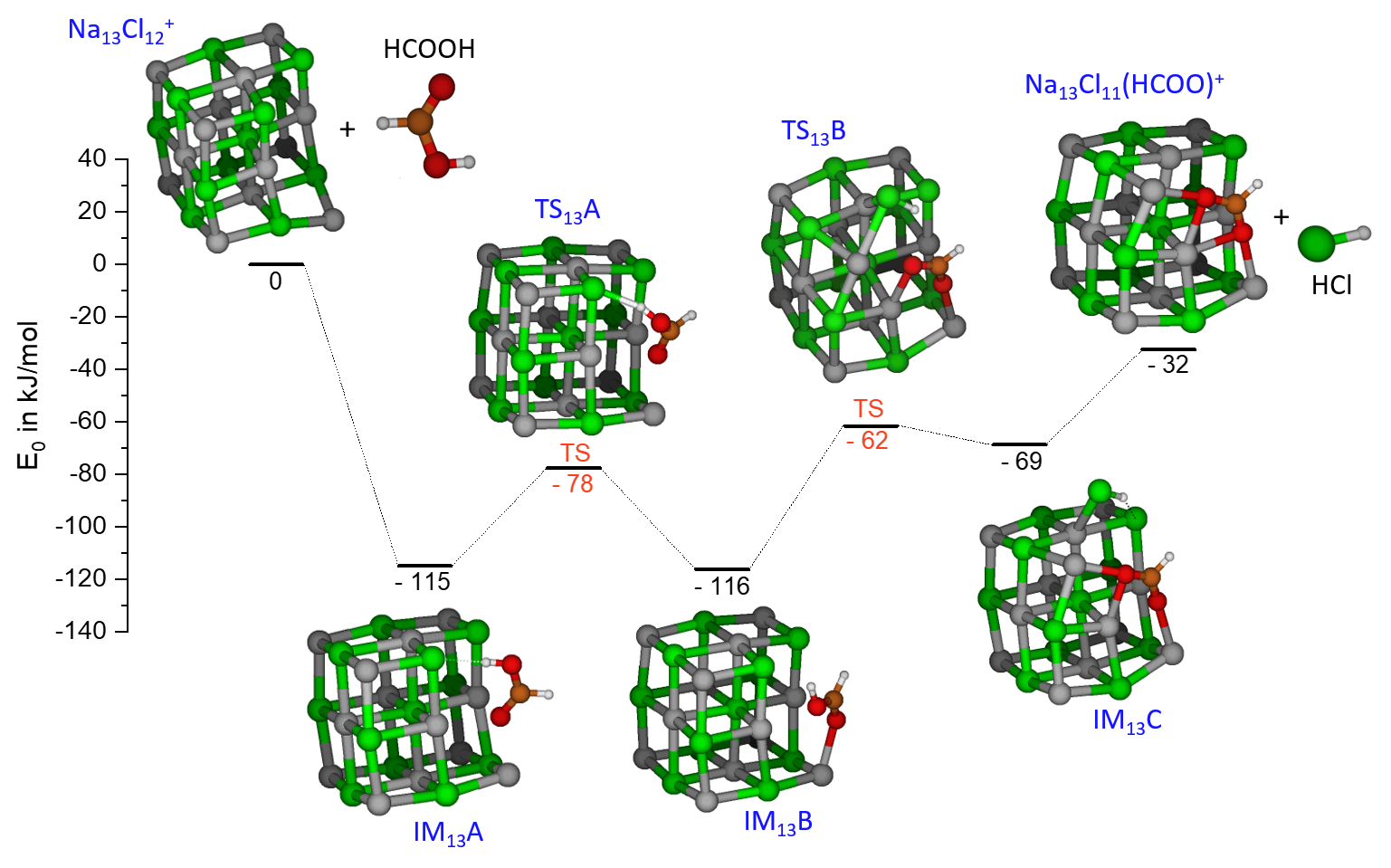


**Figure S2.** Minimum energy pathway for the reaction Na_13_Cl_12_^+^ + HCOOH → Na_13_Cl_11_(HCOO)^+^ + HCl.


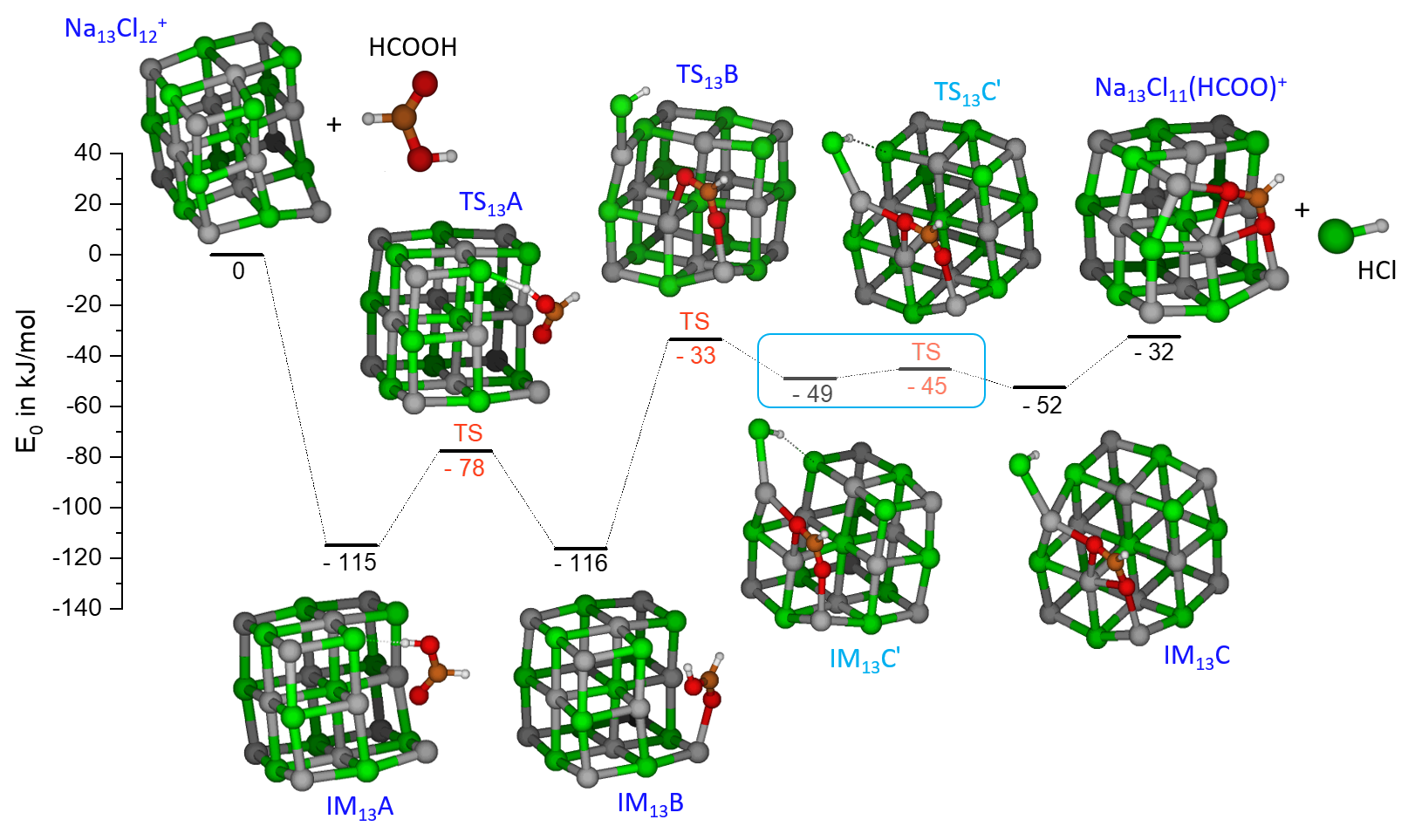


**Figure S3.** Alternative potential energy surface for the reaction Na_13_Cl_12_^+^ + HCOOH → Na_13_Cl_11_(HCOO)^+^ + HCl.


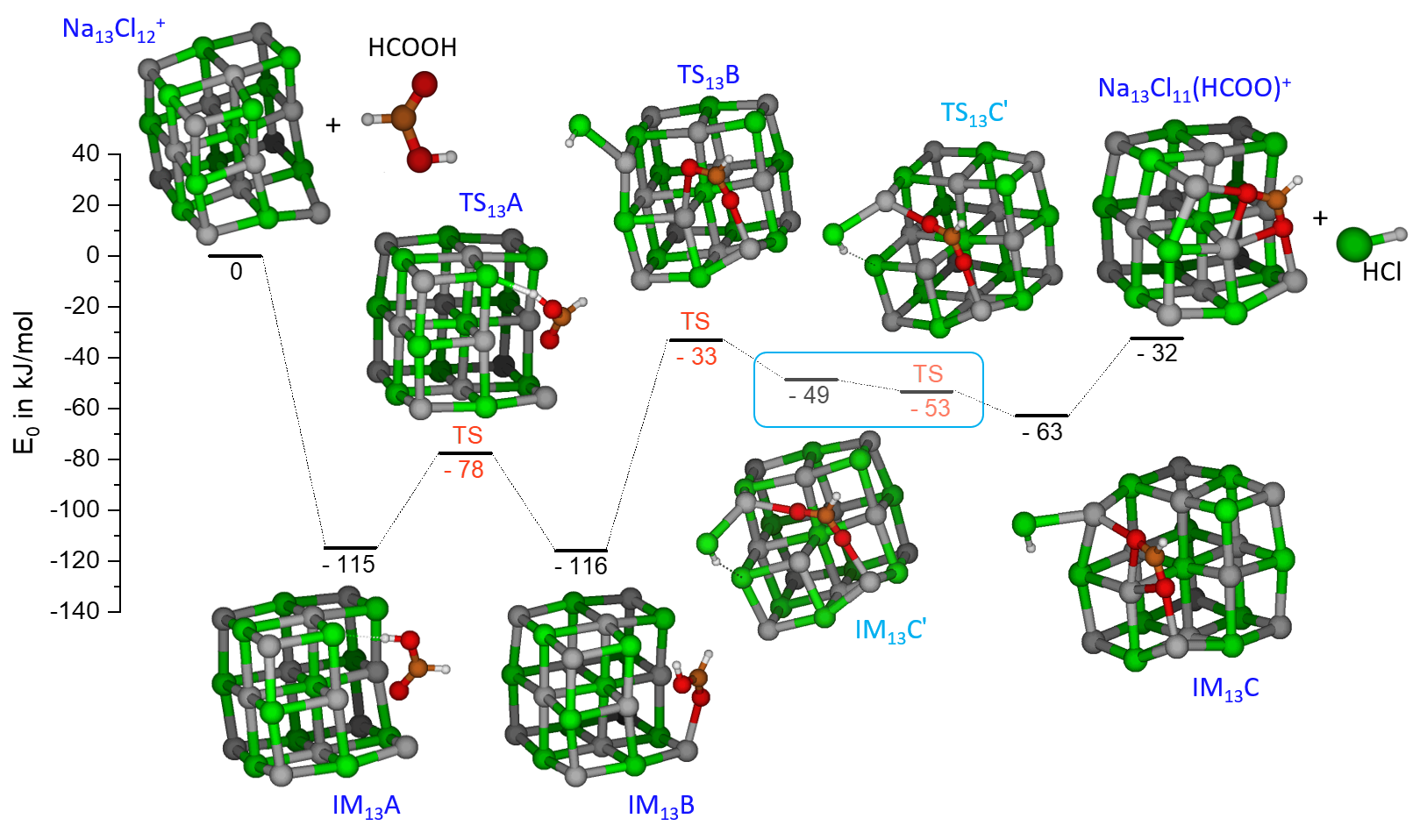


**Figure S4.** Alternative potential energy surface for the reaction Na_13_Cl_12_^+^ + HCOOH → Na_13_Cl_11_(HCOO)^+^ + HCl.


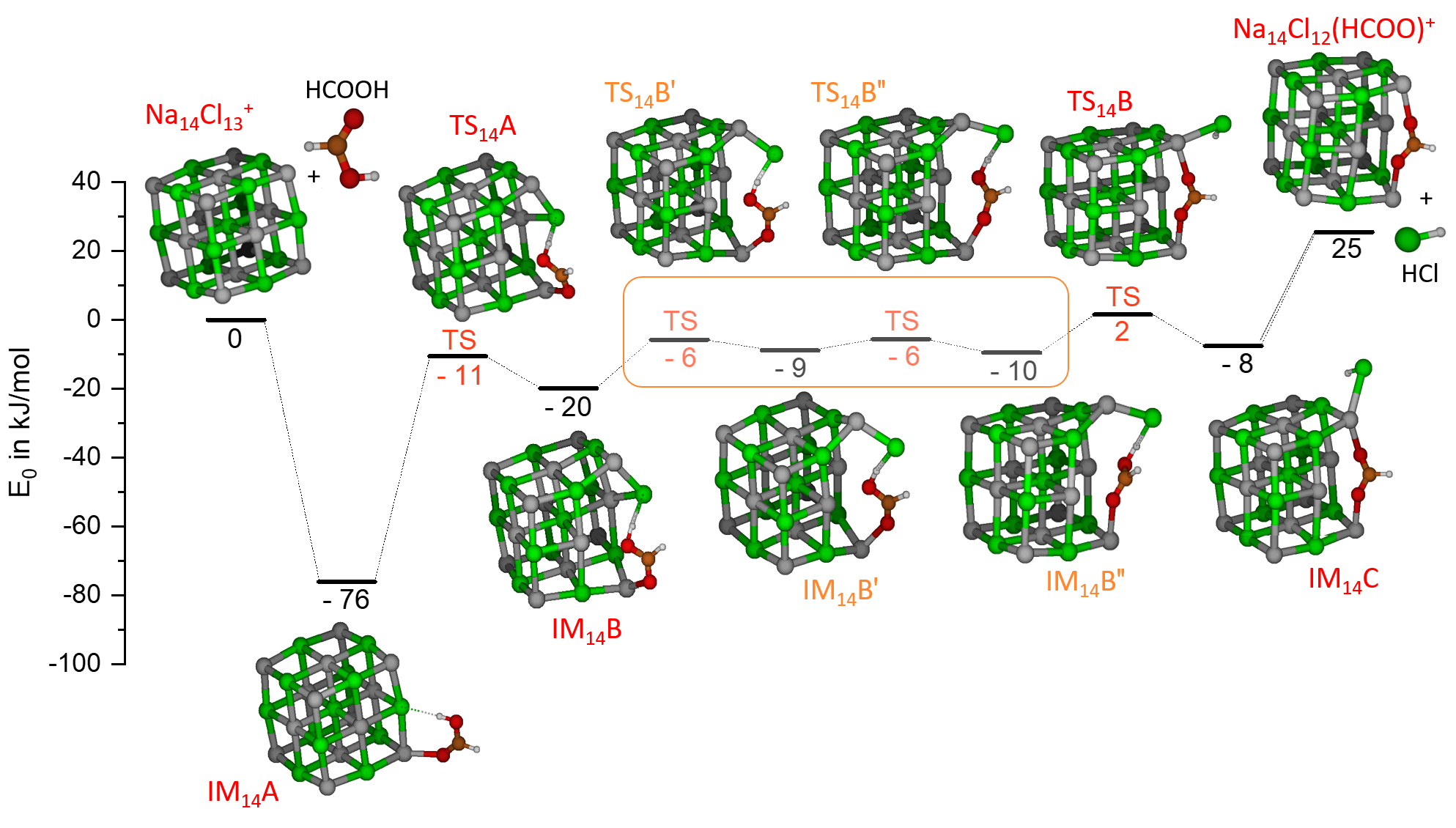


**Figure S5.** Minimum energy pathway for the reaction Na_14_Cl_13_^+^ + HCOOH → Na_14_Cl_12_(HCOO)^+^ + HCl.


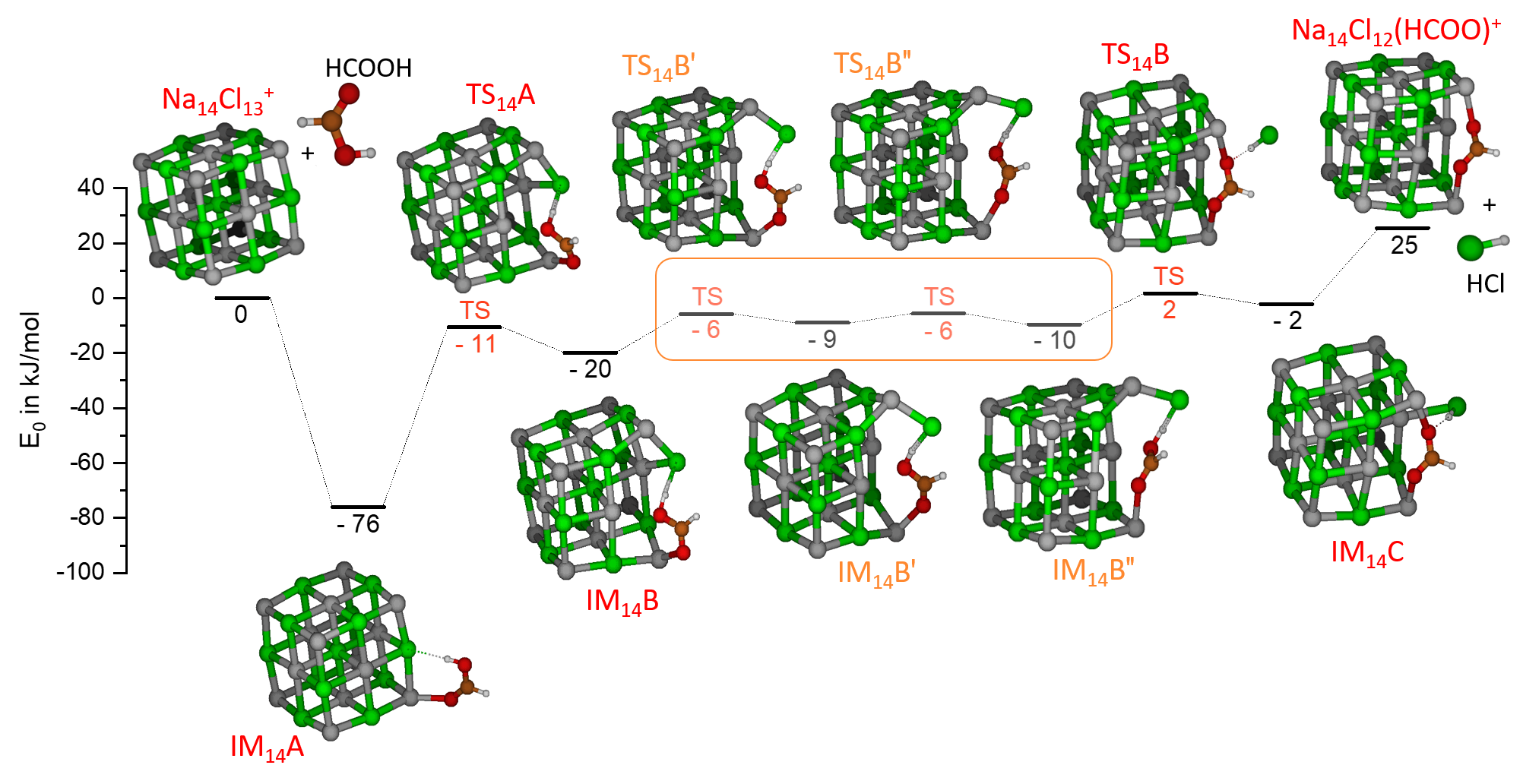


**Figure S6.** Alternative potential energy surface for the reaction Na_14_Cl_13_^+^ + HCOOH → Na_14_Cl_12_(HCOO)^+^ + HCl.


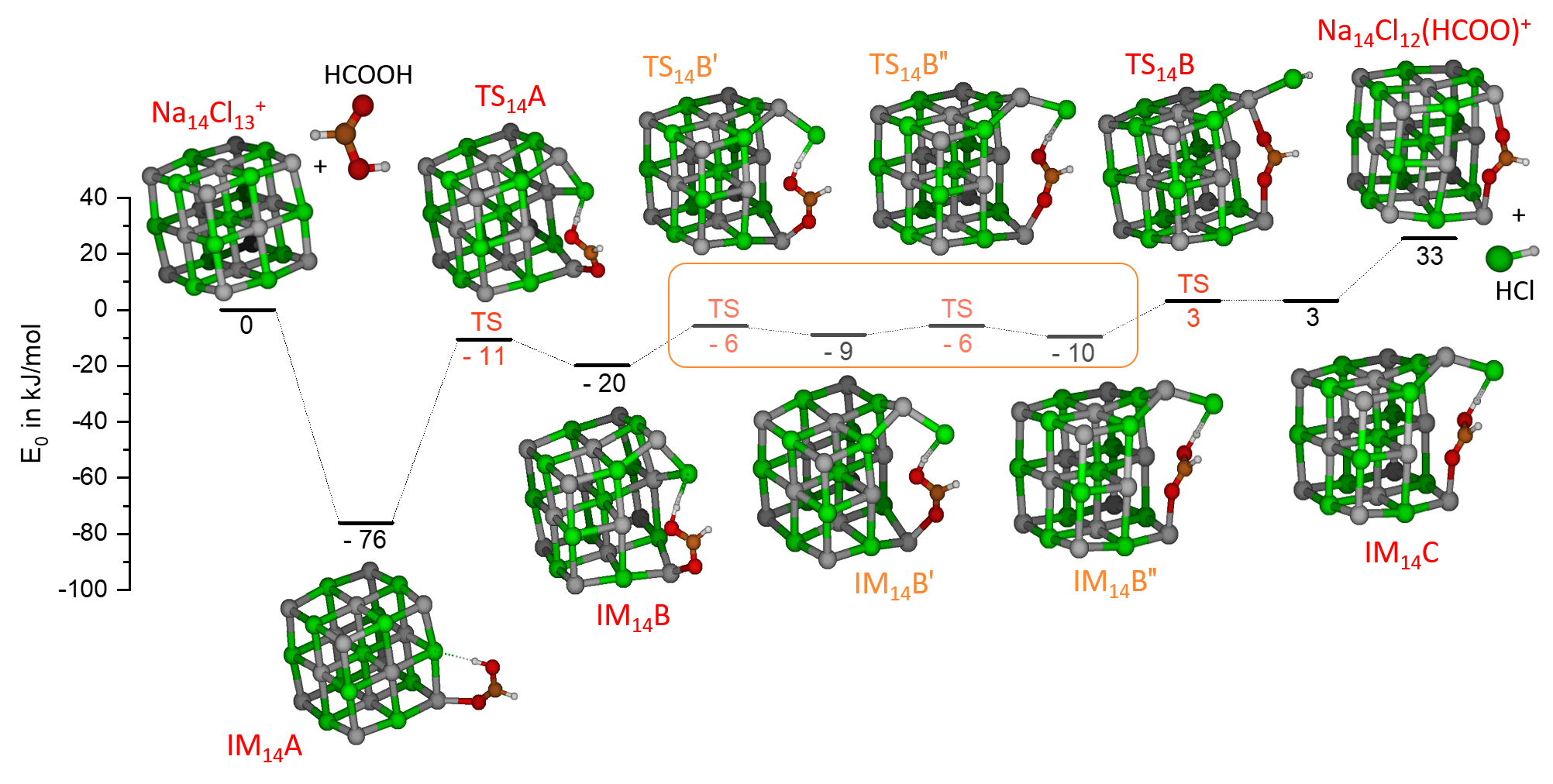


**Figure S7.** Alternative potential energy surface for the reaction Na_14_Cl_13_^+^ + HCOOH → Na_14_Cl_12_(HCOO)^+^ + HCl.


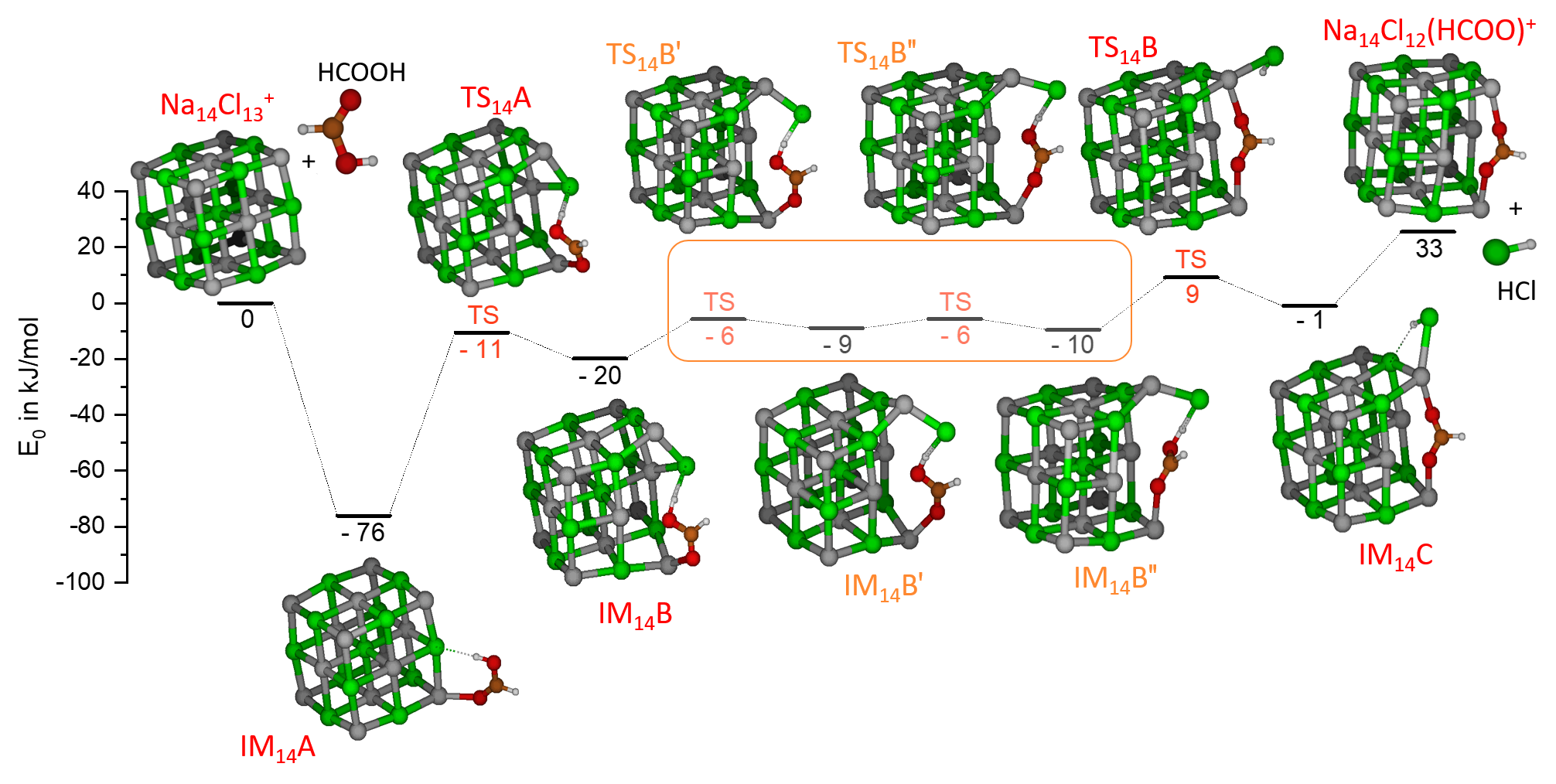


**Figure S8.** Alternative potential energy surface for the reaction Na_14_Cl_13_^+^ + HCOOH → Na_14_Cl_12_(HCOO)^+^ + HCl.


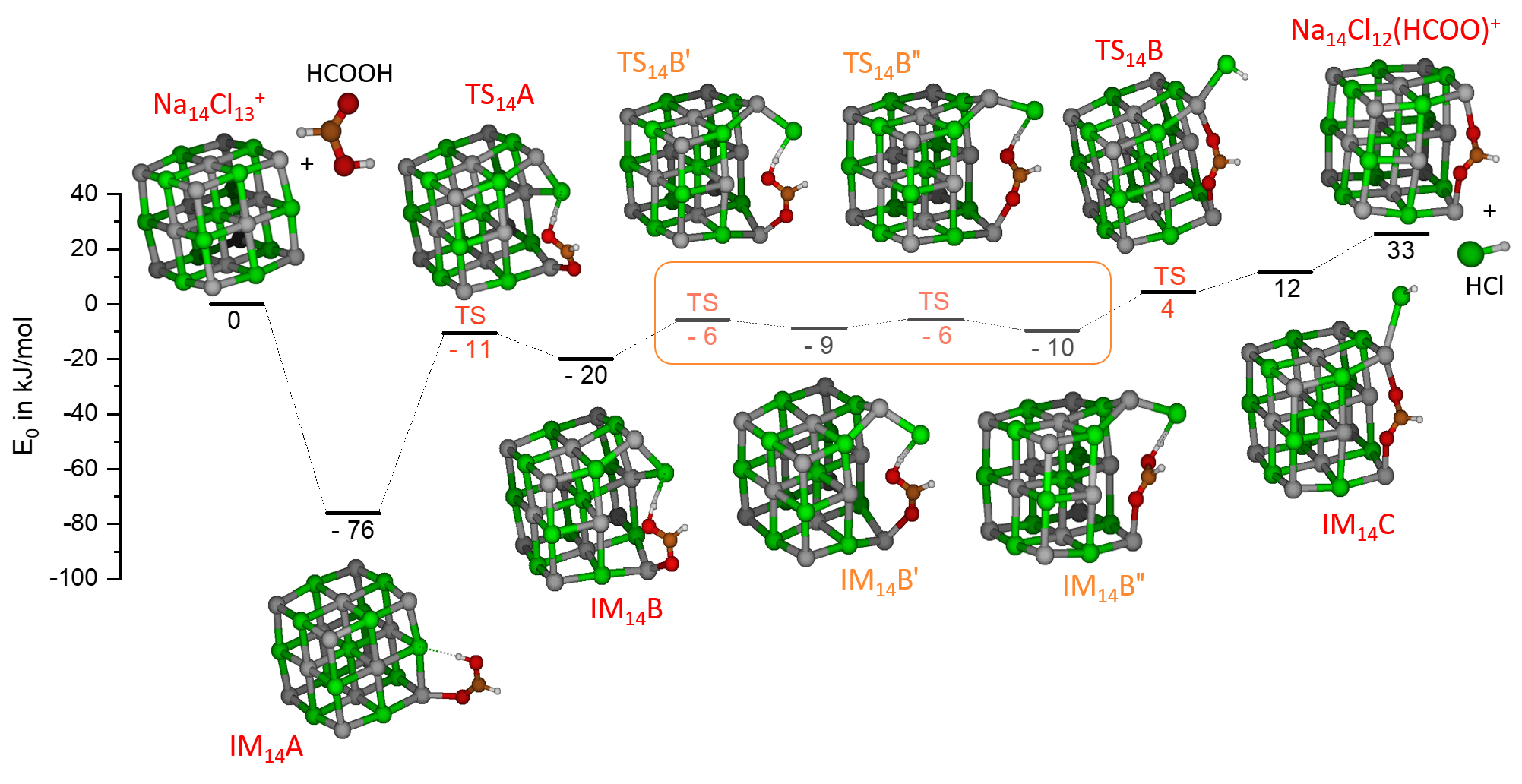


**Figure S9.** Alternative potential energy surface for the reaction Na_14_Cl_13_^+^ + HCOOH → Na_14_Cl_12_(HCOO)^+^ + HCl.

6. Thermochemical Cycle

The energy of **Reaction (3),** here **Reaction (S4),** is calculated from literature values of HCl and HCOOH deprotonation reactions in the gas phase^[27]^ and ΔE_0_ = -32 kJ mol^-1^ of **Reaction (2)**, here **Reaction (S3)**.

HCl → H^+^ + Cl^-^ Δ_r_H = 1396±8.9 kJ mol^-1^ (S1)

HCOO^-^ + H^+^ → HCOOH Δ_r_H = -1449±5 kJ mol^-1^ (S2)

Na_13_Cl_12_^+^ + HCOOH → Na_13_Cl_11_HCOO^+^ + HCl ΔE_0_ = -32±8 kJ mol^-1^ (S3)

**Sum:** Na_13_Cl_12_^+^ + HCOO^-^ → Na_13_Cl_11_HCOO^+^ + Cl^-^ ΔE_0_ = -85±13 kJ mol^-1^ (S4)

7. GOAT for Na_13_Cl_12_^+^ and Na_14_Cl_13_^+^ Clusters

Lowest lying structures of the pure Na_13_Cl_12_^+^ and Na_14_Cl_13_^+^ clusters, verified by GOAT at XTB-GNF1 level and reoptimization at r^2^SCAN-3c level are plotted in **Figure S10**. Relative energies were obtained by the computational protocol outlined above. Strikingly, the minimum energy isomer of the magic Na_14_Cl_13_^+^, which is the perfect cube resembling a section of the crystal lattice, is 67 kJ mol^-1^ more stable than the next higher lying isomer. Even higher lying isomers are at least 112 kJ mol^-1^ higher in energy. Thus, the magic cluster will most likely only be present in form of a perfect cube, representing a section of bulk salt. In contrast, there are several energetically accessible higher lying isomers for the defect-bearing Na_13_Cl_12_^+^.


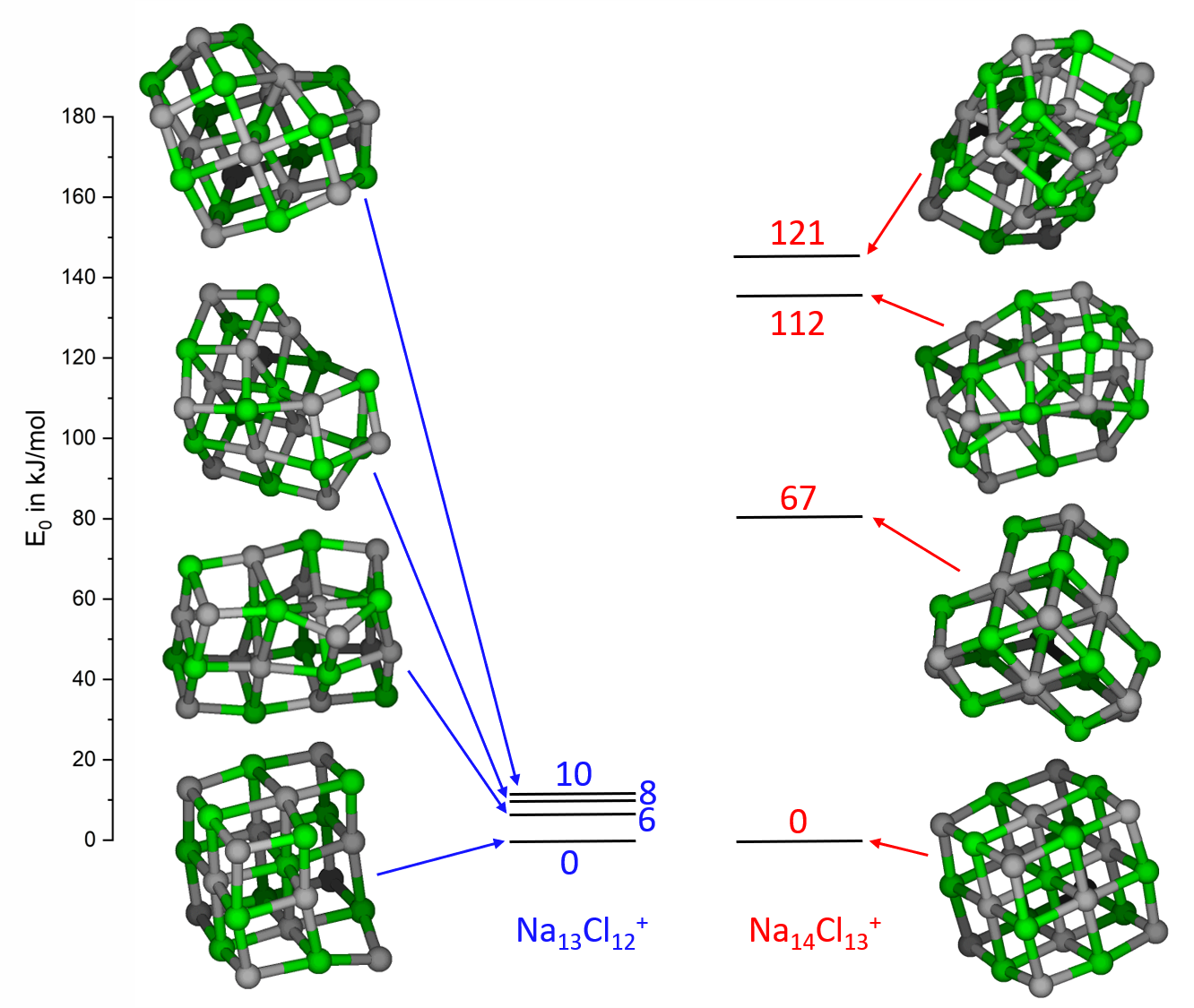


**Figure S10.** Lowest lying structures of the pure Na_13_Cl_12_^+^ and Na_14_Cl_13_^+^ clusters. Energies are obtained at the RI-MP2-F12/cc-pVDZ-F12//r2SCAN-3c level.

8. Analysis of Molecular Dynamic Simulations

The reactivity of sodium chloride cluster ions (NaCl)*_x_*Na^+^ towards formic acid was studied using density functional theory based molecular dynamics (DFT-MD) calculation. Particularly, an enhanced sampling technique, on-the-fly probability enhanced sampling (OPES), was employed to accelerate the sampling of a rare event which was the acid displacement reaction in gas-phase sodium chloride cluster ions by formic acid. Before initiating OPES production runs, well-tempered metadynamics DFT-MD were performed to obtain a preliminary guess of the FES because of unknown reaction barriers. Well-tempered metadynamics is less sensitive to the selection of CVs compared to the OPES method. For example, only two CVs were defined for well-tempered metadynamics and afforded to sample the acid displacement reaction. The barrier estimated were 50 kJ mol^−1^ and 91 kJ mol^−1^ for Na_13_Cl_12_(HCOOH)^+^ cluster (left) and Na_14_Cl_13_(HCOOH)^+^ cluster (right). In contrast, the OPES method required the inclusion of 3^rd^ CV, O−H interaction to have meaningful sampling of the FES.


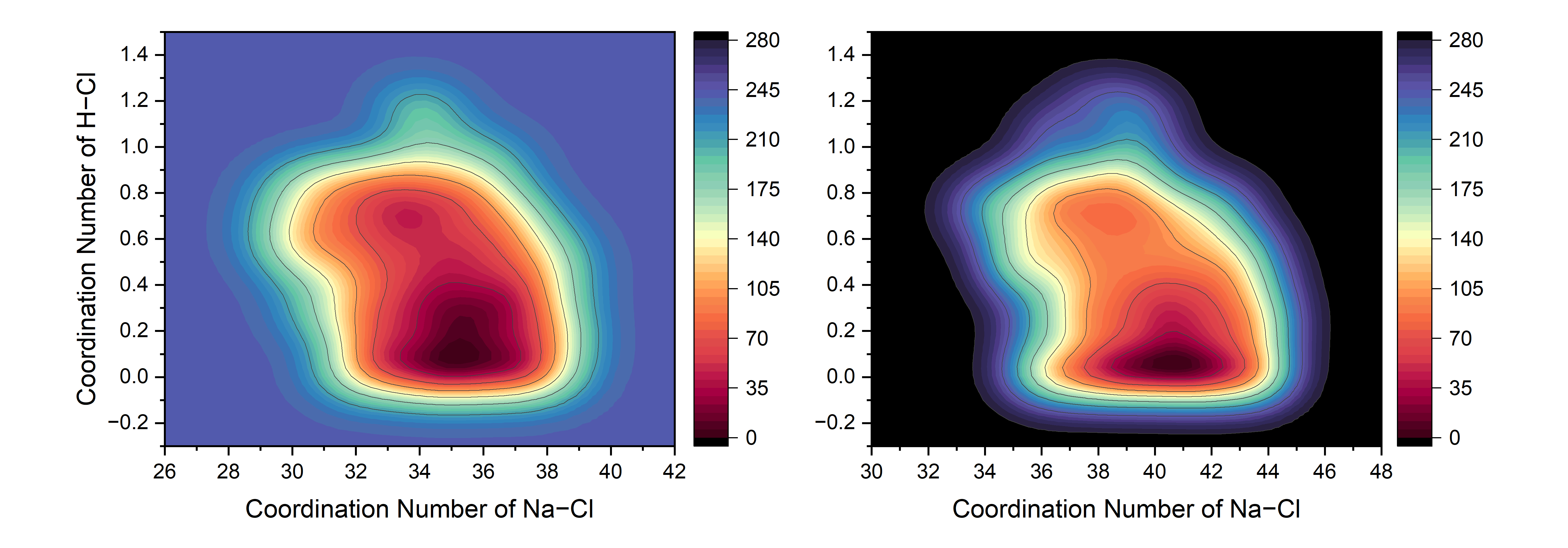


P

R

P

R

**Figure S11.** FES for Na_13_Cl_12_(HCOOH)^+^ cluster (left) and Na_14_Cl_13_(HCOOH)^+^ cluster (right). constructed from well-tempered metadynamics under NVT ensemble at 300 K.

**a0**

**b**

**c**


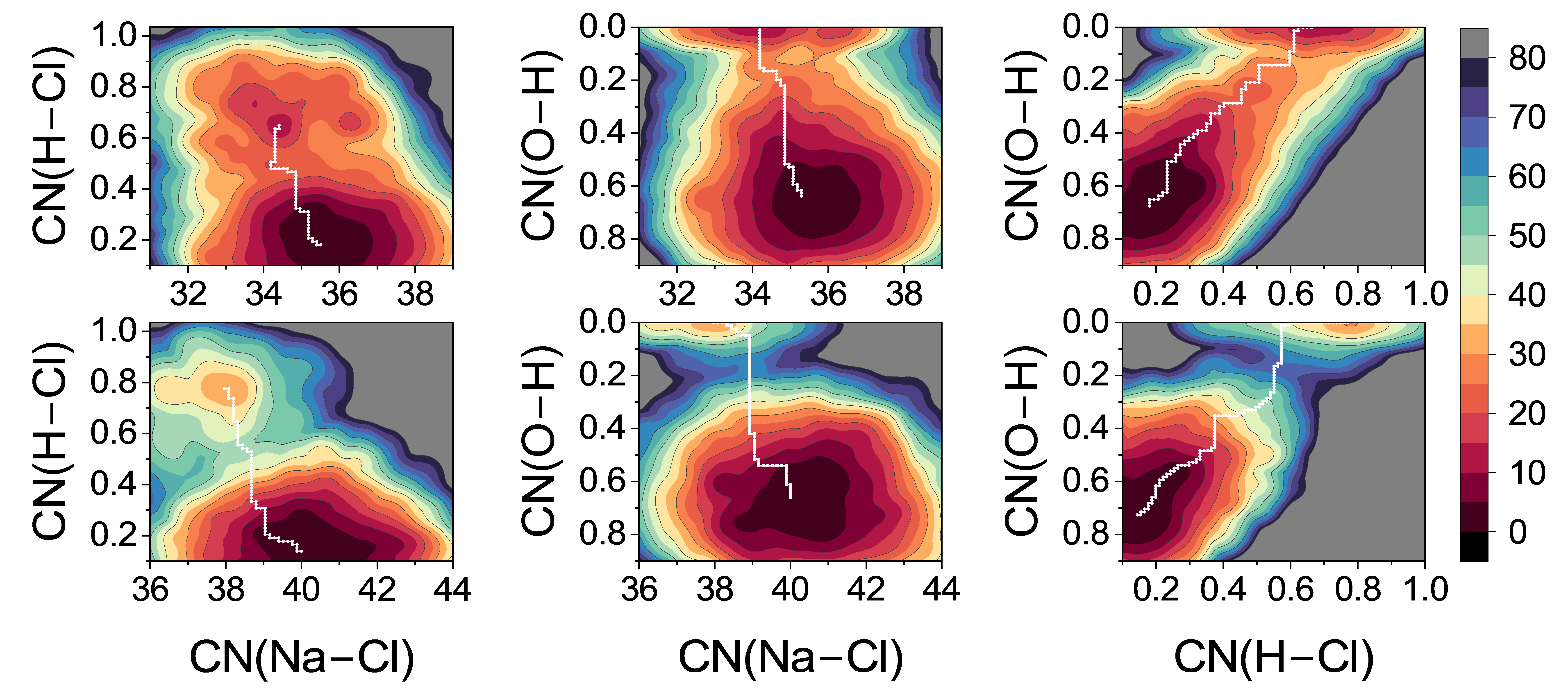


**Figure S12.** Projected 2D free energy surfaces (FES) for Na_13_Cl_12_(HCOOH)^+^ cluster (upper panel) and Na_14_Cl_13_(HCOOH)^+^ cluster (lower panel). CN refers to Coordination Number.

Figure S12 shows the final free energy surface (FES) for the reactions for Na_13_Cl_12_^+^ + HCOOH → Na_13_Cl_11_(HCOO)^+^ + HCl (upper panel) and Na_14_Cl_13_^+^ + HCOOH → Na_14_Cl_12_(HCOO)^+^ + HCl (lower panel) from the OPES production runs. The FES were calculated using the reweighting and block-averaging scheme from an ensemble average of all independent trajectories. As three collective variables were defined in the enhanced DFT-MD simulations, three projected 2D FES were generated by integrating out the thermodynamic contribution of the nonchosen CV. For instance, lower-lying points in Figure S12a has higher probability of accessing greater number of microstates defined by CN (O−H) in the trajectories. Next, minimum free energy pathway was searched on each 2D FES to estimate the barrier for the acid displacement reaction. The highest minimum free energy pathway was identified in Figure S12c.

The evolution of the OPES 3D collective variables over time for the representative trajectories were illustrated in Figure S13. The proton transfer process involves three key chemical interactions. The proton transfer was shown by the evolution of O−H (blue line) and Cl−H (red line) bond distances over time. For Na_13_Cl_12_(HCOOH)^+^ cluster, the proton transfer was observed three times in 40 ps. For Na_14_Cl_13_(HCOOH)^+^ cluster, the proton transfer was observed three times in 30 ps. During the proton transfer process, the loss of Na−Cl interactions (black lines) were concomitantly observed for both cluster. Notably, the formation of the Cl−H covalent bond in Na_13_Cl_12_(HCOOH)^+^ cluster did not necessarily sacrifice existing Na−Cl interactions (see the shaded regions in panel b and d in Figure S13). To complete the reaction, three Na−Cl interactions and four Na−Cl interactions must be overcome for Na_13_Cl_12_(HCOOH)^+^ and Na_14_Cl_13_(HCOOH)^+^ cluster, respectively.


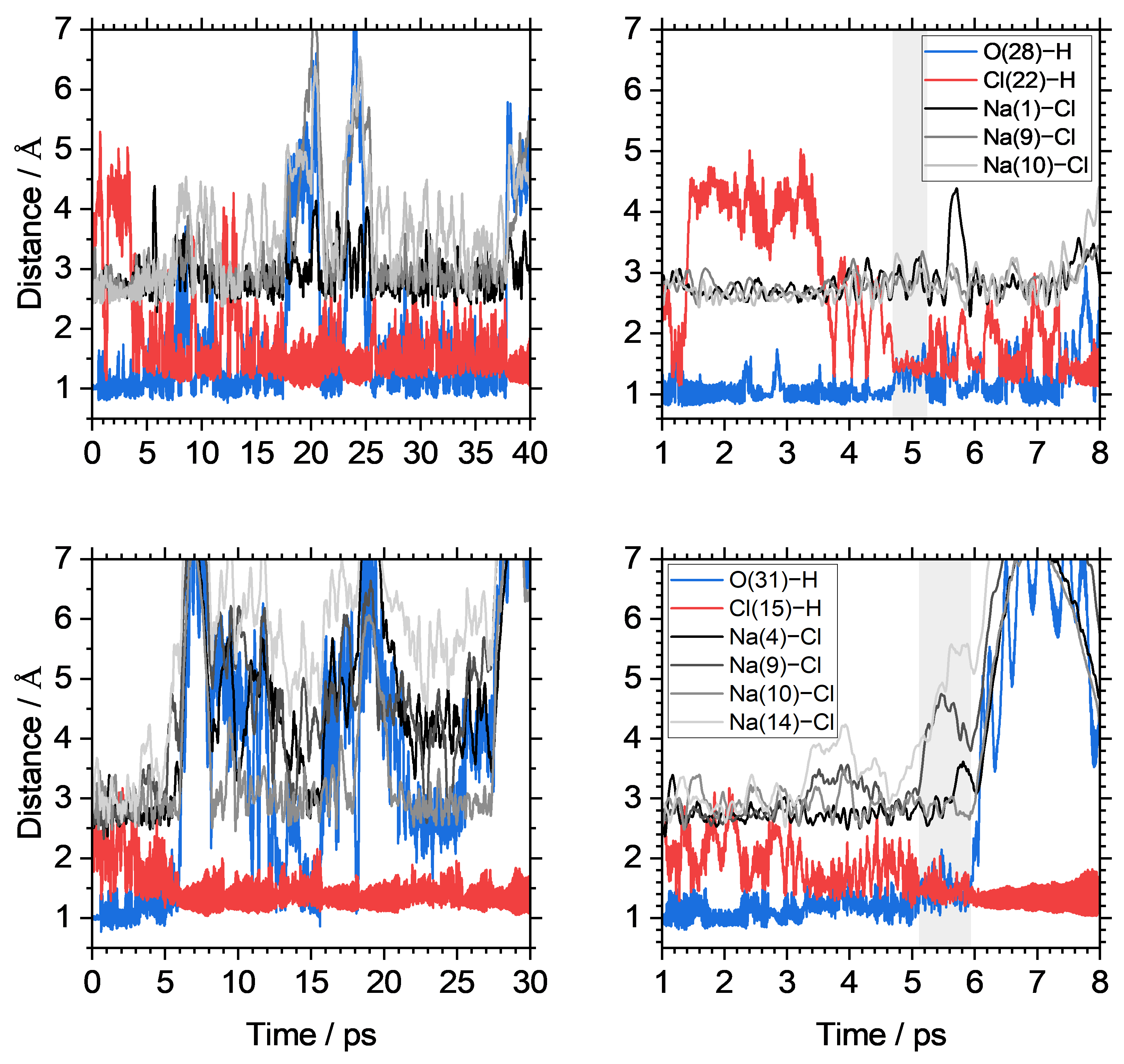


**a**

**b**

**c**

**d**

**Figure S13.** Distance correlation extracted from representative OPES DFT-MD trajectory for Na_13_Cl_12_(HCOOH)^+^ cluster (panel a and b) and Na_14_Cl_13_(HCOOH)^+^ cluster (panel c and d).

The block-averaging scheme used for reconstruction of free energy surface (FES) can be useful to assess the convergence of the FES and its range of error. The final FES was based on an ensemble average of fully independent trajectories: a total of 9 trajectories for Na_13_Cl_12_(HCOOH)^+^ cluster and a total of 14 trajectories for Na_14_Cl_13_(HCOOH)^+^ cluster. The evolution of CVs and deposited bias in each frame were concatenated into a combined dataset before processing. The block-averaging scheme divides the combined dataset into a number of blocks and subsequently calculates free energy of each block using the $F\left( s \right)=-k_{B}T\log\left( p\left( s \right) \right)$ relation. The final FES was the average of each block. The error estimation was calculated from averaging the blocks. Figure S14a shows that the estimated errors were below 10% of the barriers of the FES. Block size of 15 was used for calculating the error estimation for the FES of CN (H−Cl) vs. CN (O−H) in Figure S14b.


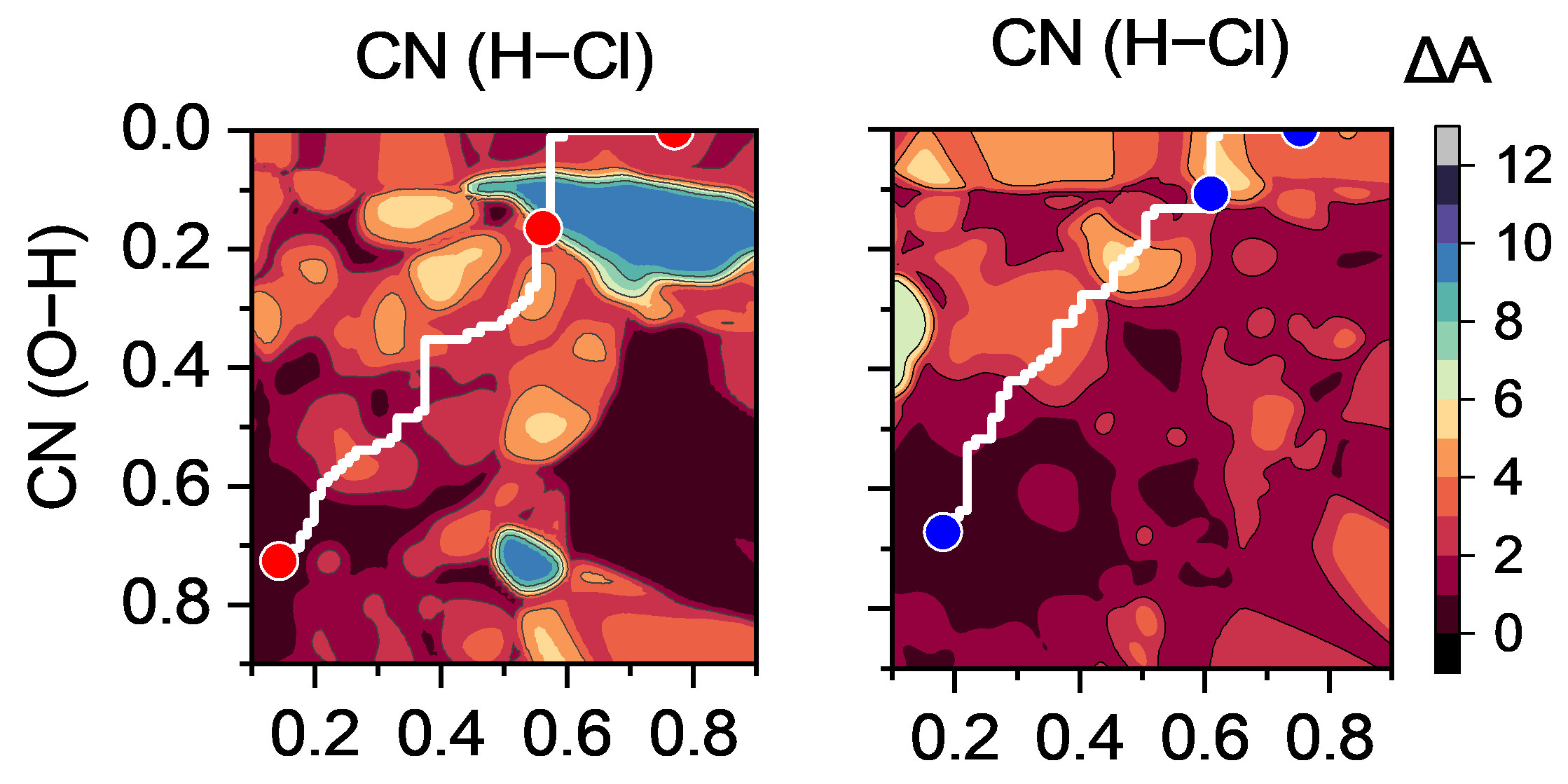

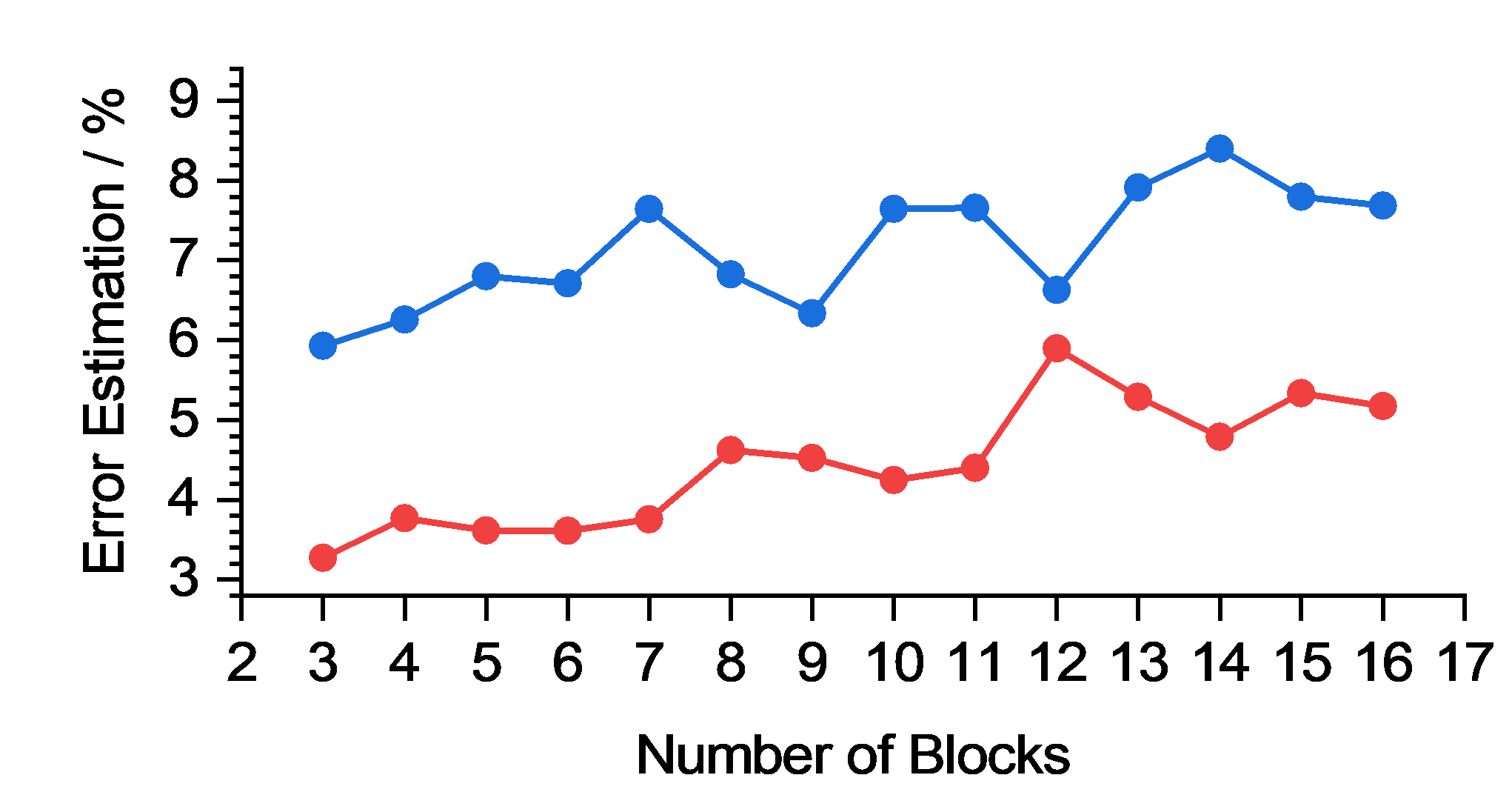


**a**

**b**

**Figure S14.** a) Error Estimation for FES of Na_13_Cl_12_(HCOOH)^+^ cluster (blue line) and Na_14_Cl_13_(HCOOH)^+^ (red line). Error is averaged from all grid points of the 2D FES for CV(H−Cl) vs. CV(O−H) and calculated as percentage of barrier in each cluster. b) Error estimation for the FES of CN (H−Cl) vs. CN (O−H).

As all reactive trajectories were independent, the error of FES could also be estimated from the variance between individual FES profiles. Individual FES profiles were calculated (see Chapter 7.1). Figure S15 shows the standard error of the mean at each point of CN (H−Cl) vs. CN (O−H).


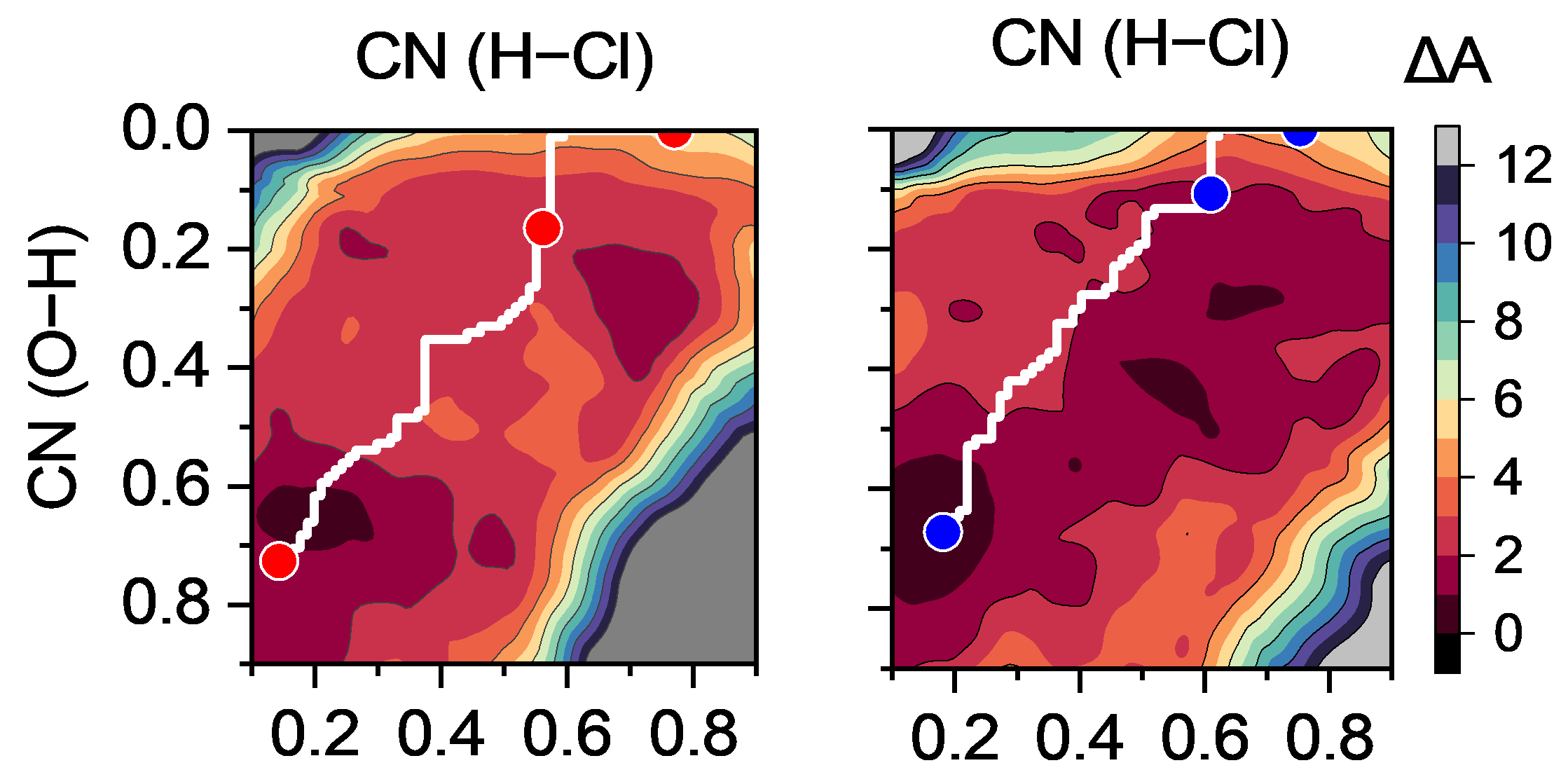


**Figure S15.** Standard error of the mean for FES of Na_13_Cl_12_(HCOOH)^+^ cluster (left panel) and Na_14_Cl_13_(HCOOH)^+^ cluster (right panel).

8.1 Individual FES for Na_13_Cl_12_(HCOOH)^+^

A total of 9 trajectories were obtained from the OPES production runs. Two different geometry inputs were employed to initiate DFT-MD simulations (see Chapter 11 for XYZ coordinates). All OPES production runs had the same parameters except for the OPES BARRIER hyperparameter which ranged from 80 to 90 kJ mol^−1^.

Input 1: Formic acid was arbitrarily positioned at a distance from the defect site.

Input 2: Formic acid was inserted into the defect site.

Input 1 - BARRIER 80 kJ mol^−1^ (60 ps) – without product state


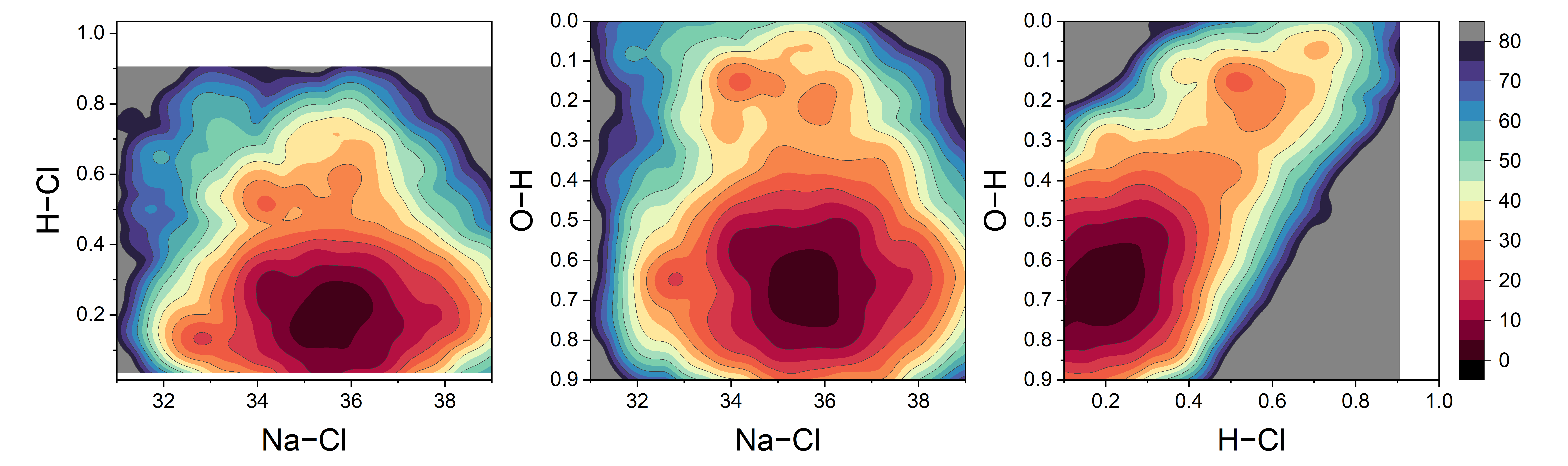


Input 1 – BARRIER 80 kJ mol^−1^ (53 ps)


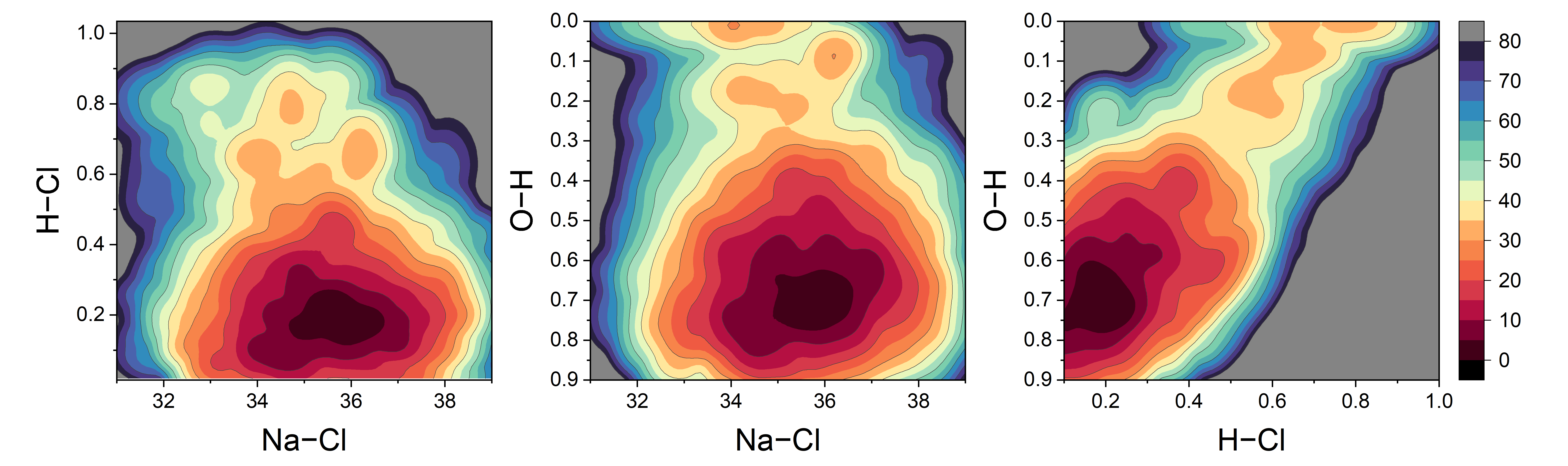


Input 2 - BARRIER 80 kJ mol^−1^ (40 ps)


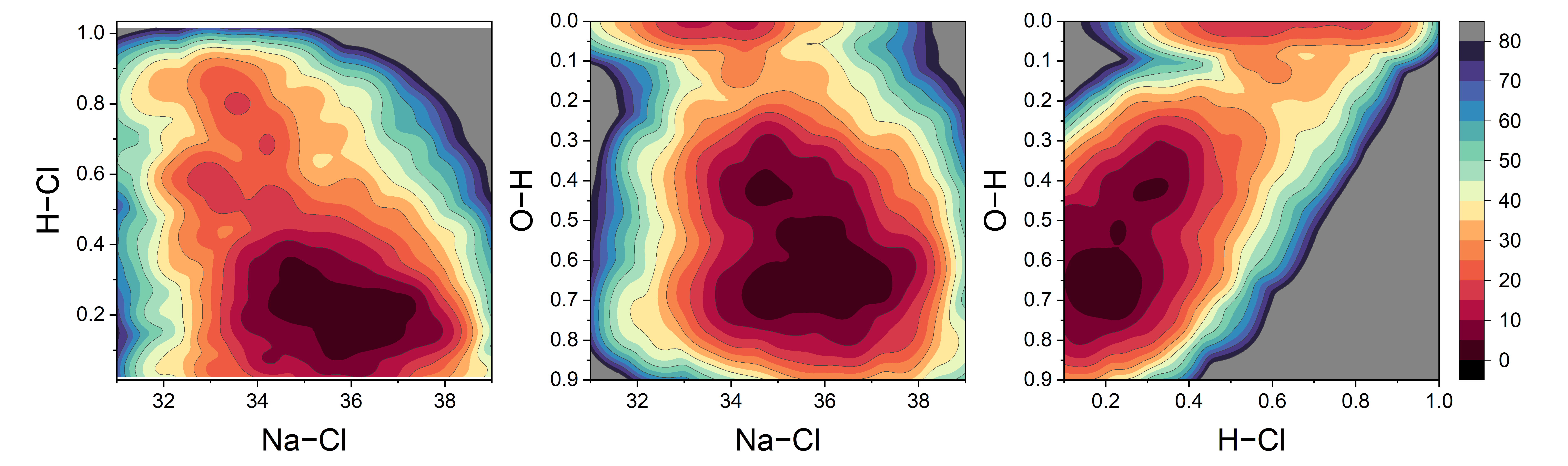


Input 2 - BARRIER 80 kJ mol^−1^ (38 ps)


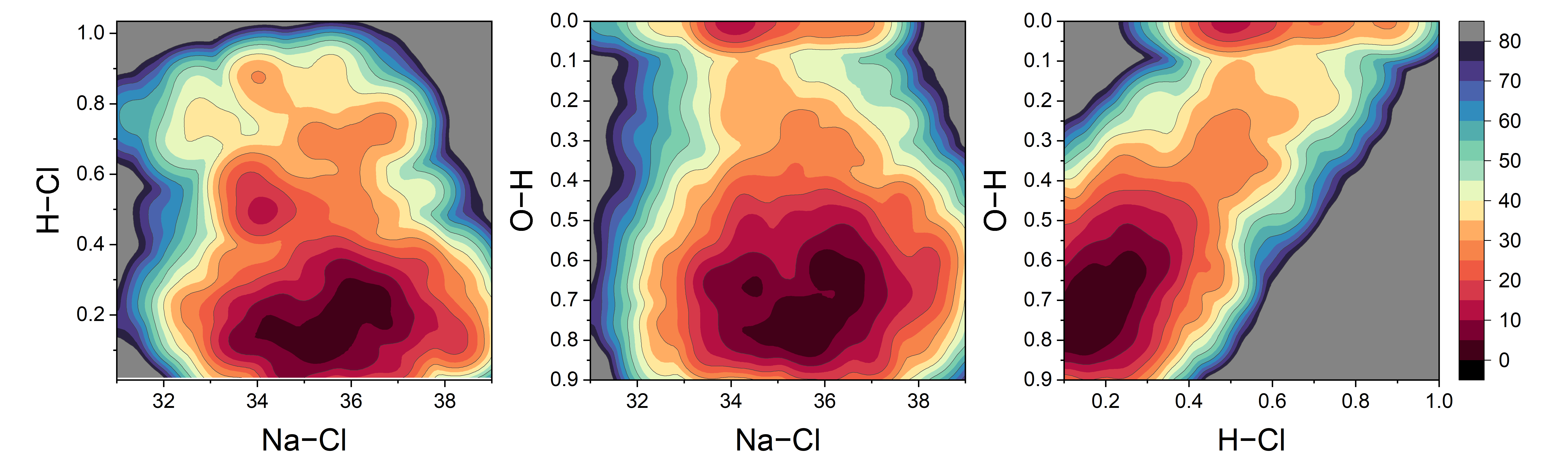


Input 2 - BARRIER 85 kJ mol^−1^ (55 ps)


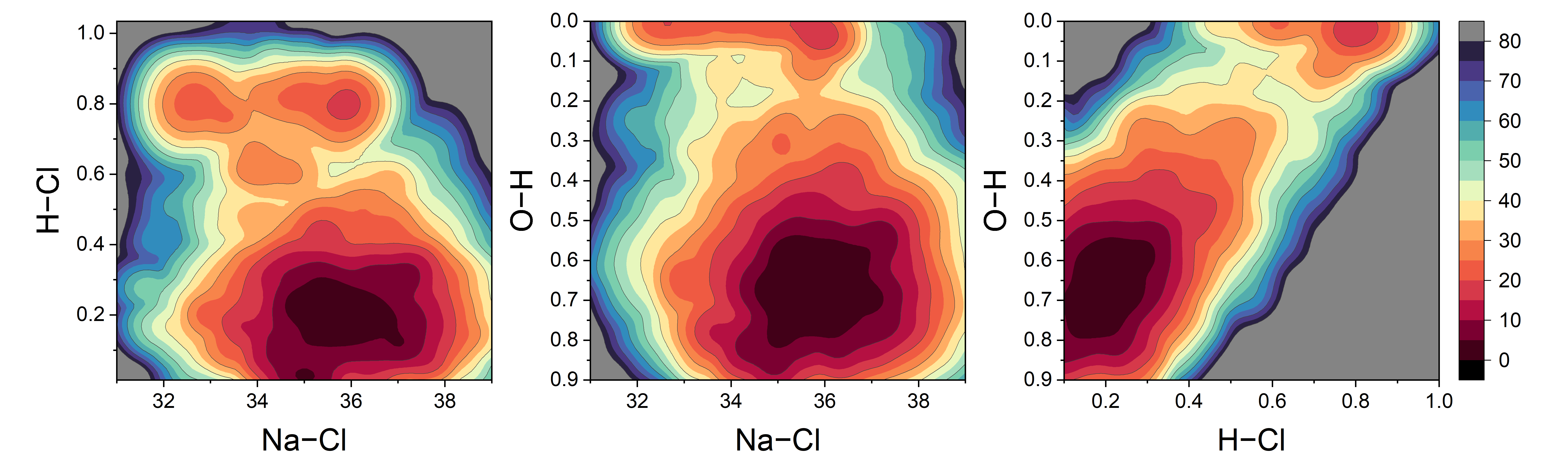


Input 1 - BARRIER 90 kJ mol^−1^ (70 ps)


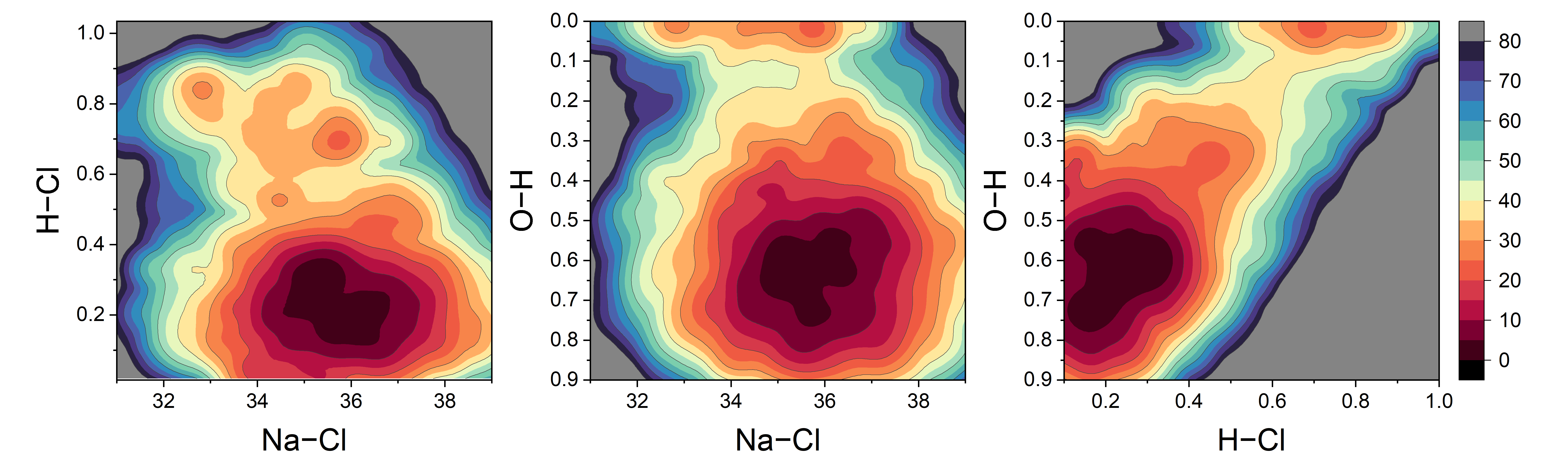


Input 1 - BARRIER 90 kJ mol^−1^ (65 ps)


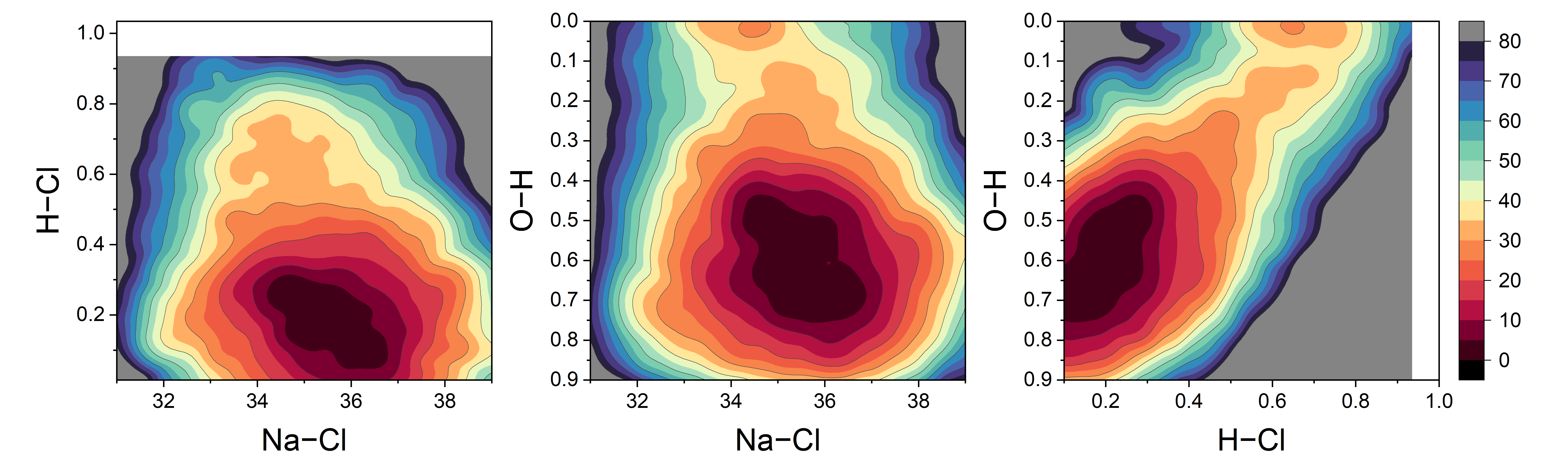


Input 2 - BARRIER 90 kJ mol^−1^ (33 ps)


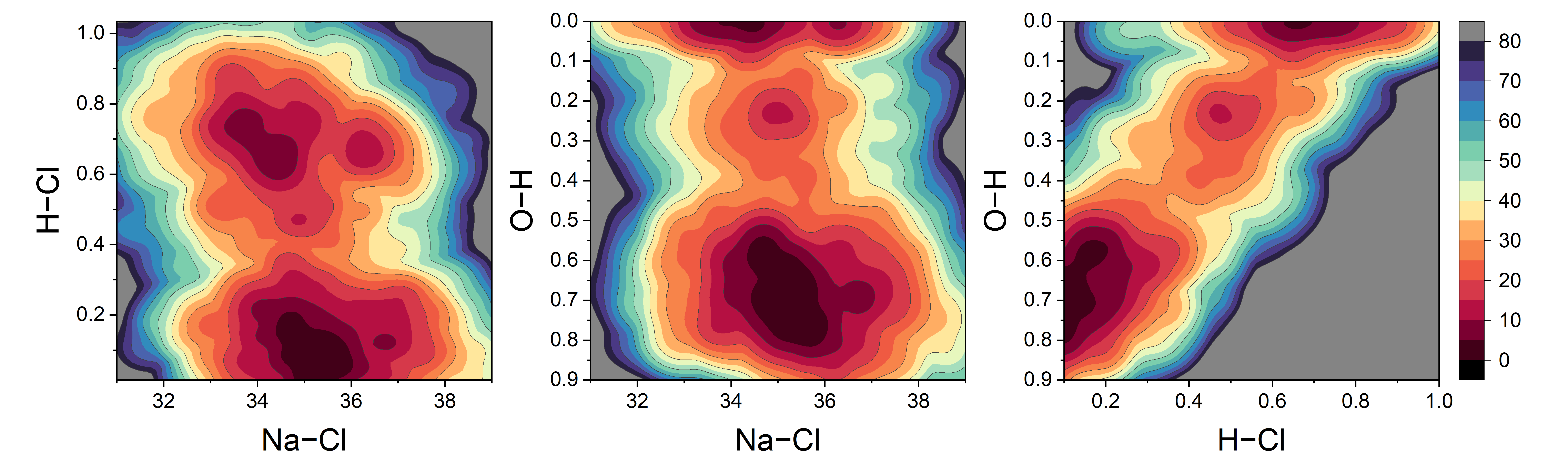


Input 2 - BARRIER 90 kJ mol^−1^ (61 ps)


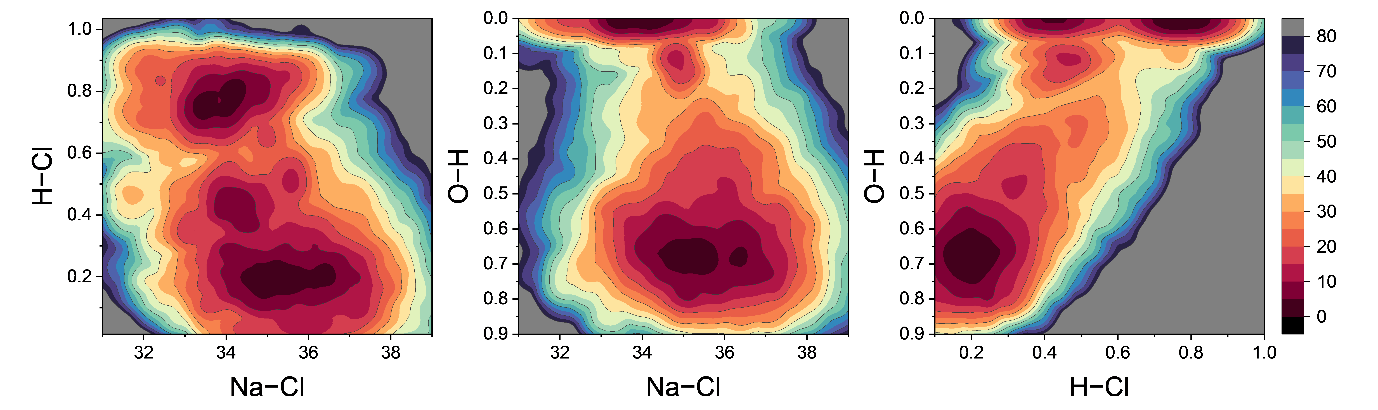


8.2 Individual FES for Na_14_Cl_13_(HCOOH)^+^

A total of 14 trajectories were obtained from the OPES production runs. Three different geometry inputs were employed to initiate DFT-MD simulations (see Chapter 11 for XYZ coordinates). All OPES production runs had the same parameters except for the OPES BARRIER hyperparameter which ranged from 80 to 90 kJ mol^−1^.

Unlike the defect-bearing cluster, the magic cluster of Na_13_Cl_12_(HCOOH)^+^ has a perfect cubic structure. Therefore, three different inputs were different atomic positions of formic acid relative to the sodium chloride cluster ions.

Input 1 - BARRIER 80 kJ mol^−1^ (38 ps) – without product state


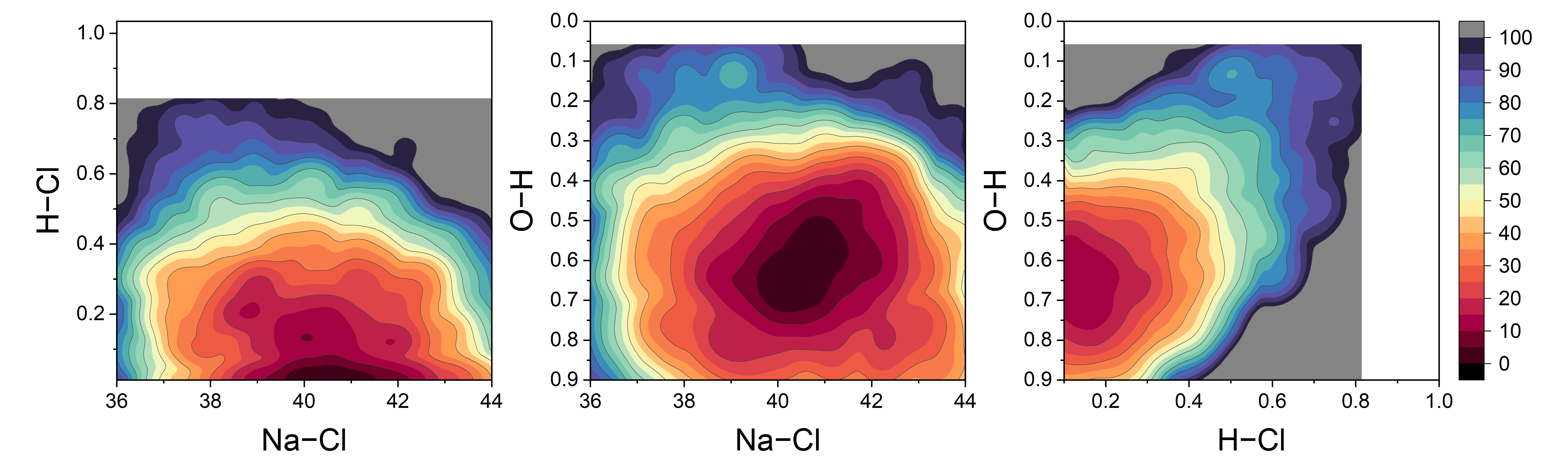


Input 2 - BARRIER 80 kJ mol^−1^ (13 ps)


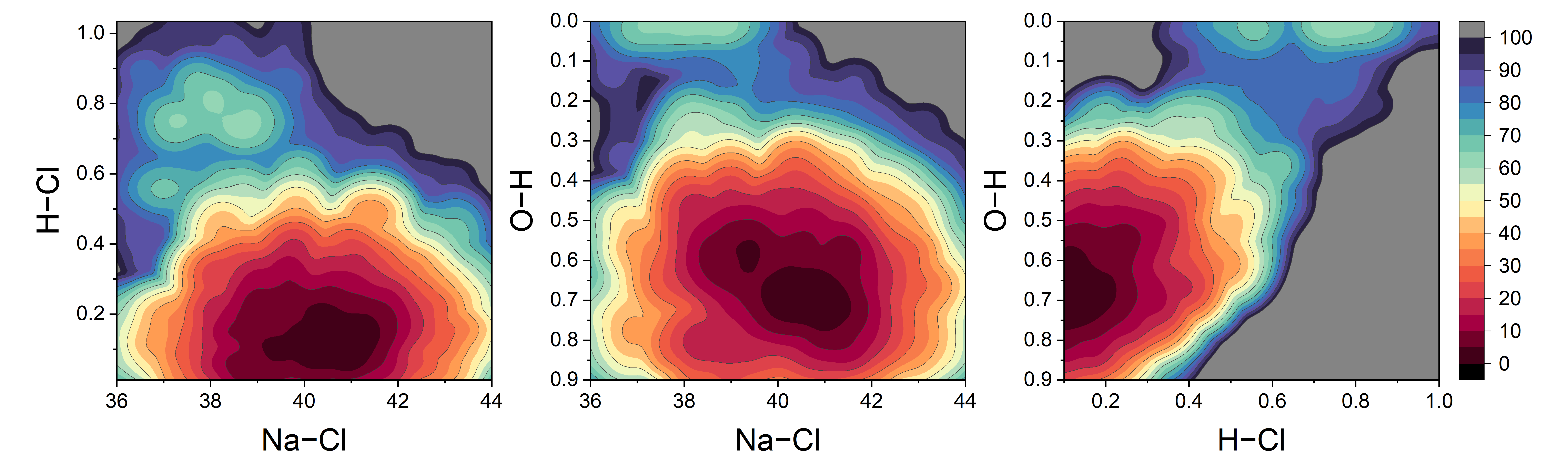


Input 3 - BARRIER 80 kJ mol^−1^ (10 ps)


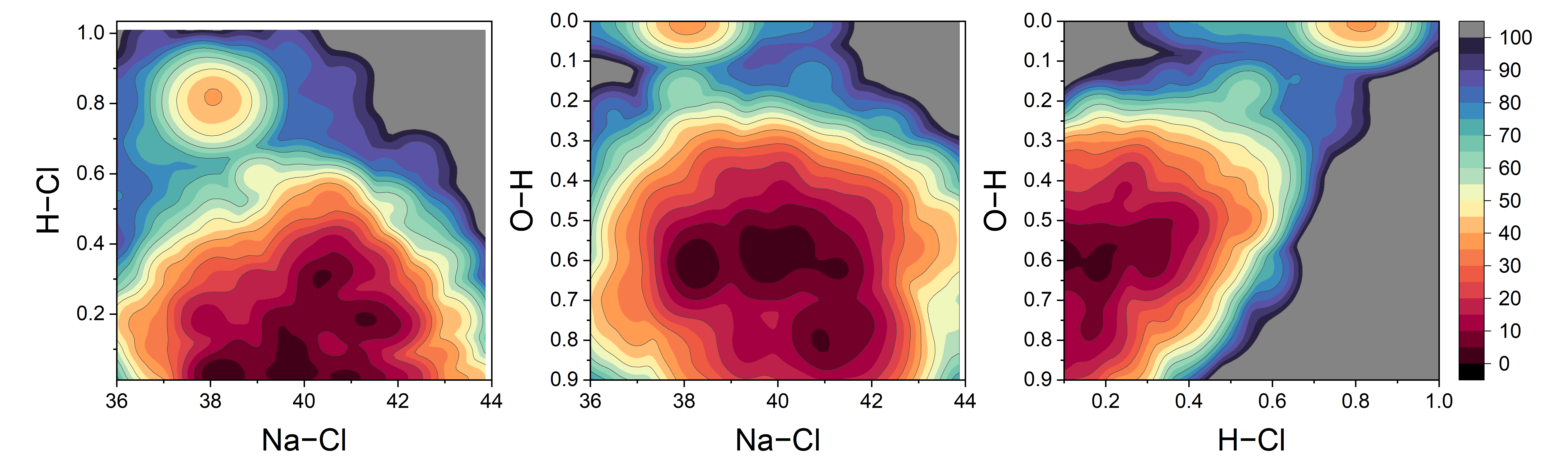


Input 1 - BARRIER 85 kJ mol^−1^ (10 ps)


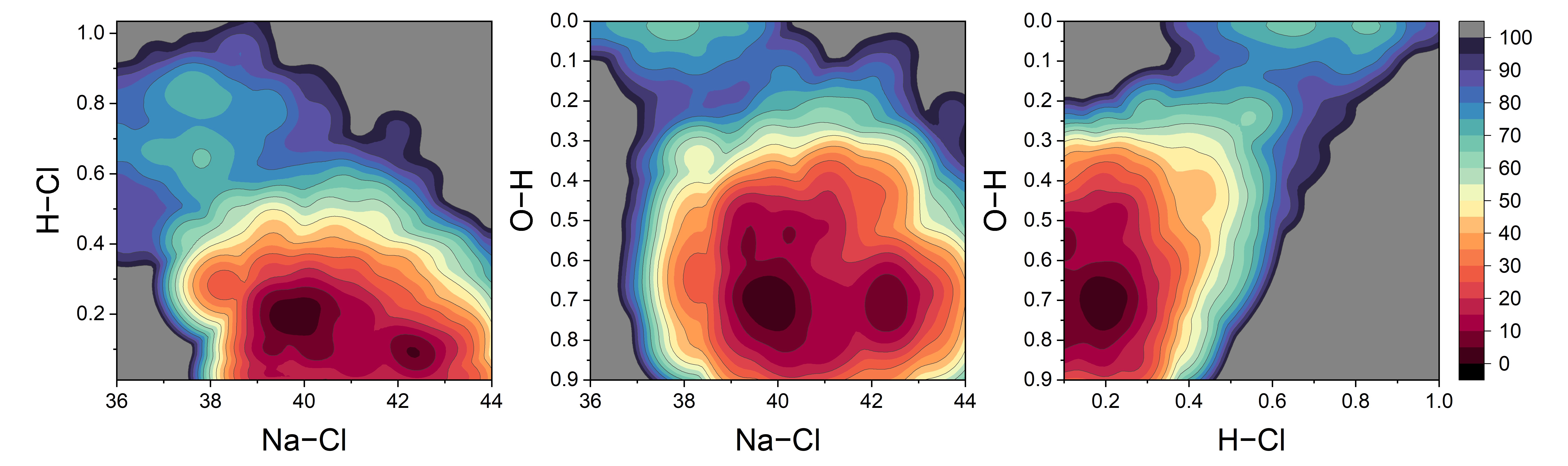


Input 1 - BARRIER 85 kJ mol^−1^ (7.5 ps)


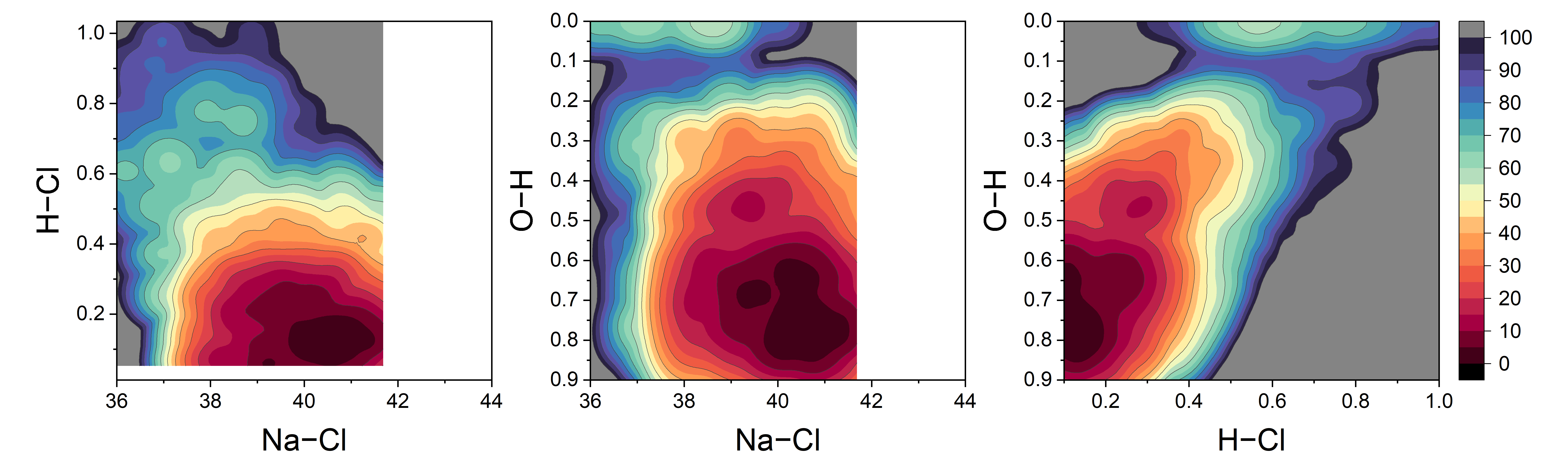


Input 1 - BARRIER 85 kJ mol^−1^ (14 ps)


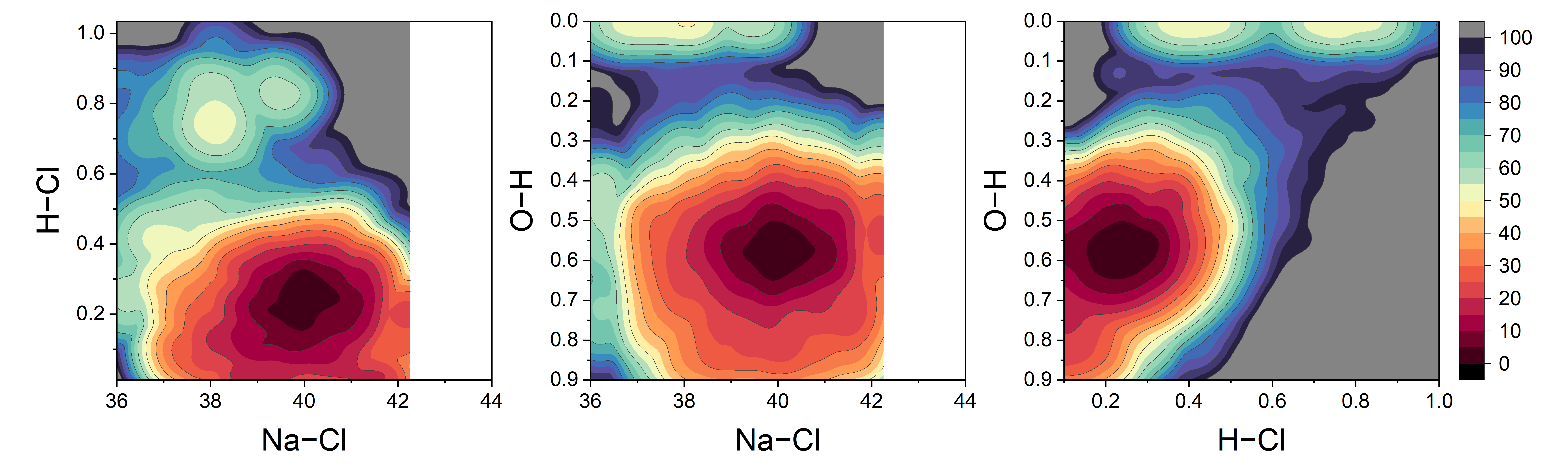


Input 2 - BARRIER 85 kJ mol^−1^ (6 ps)


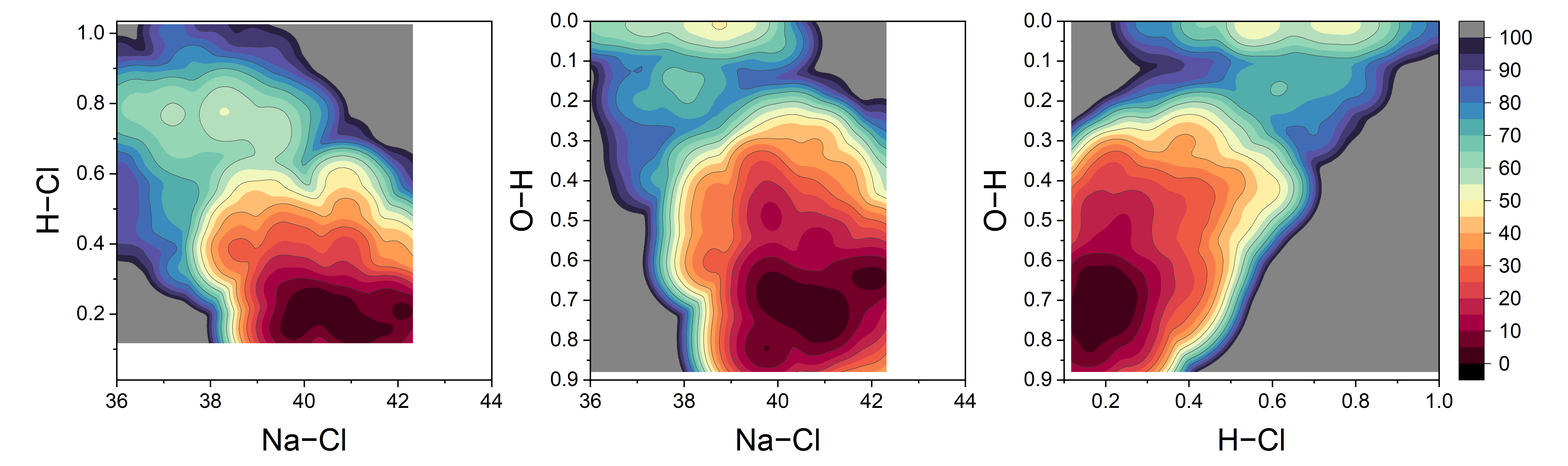


Input 2 - BARRIER 85 kJ mol^−1^ (7.5 ps)


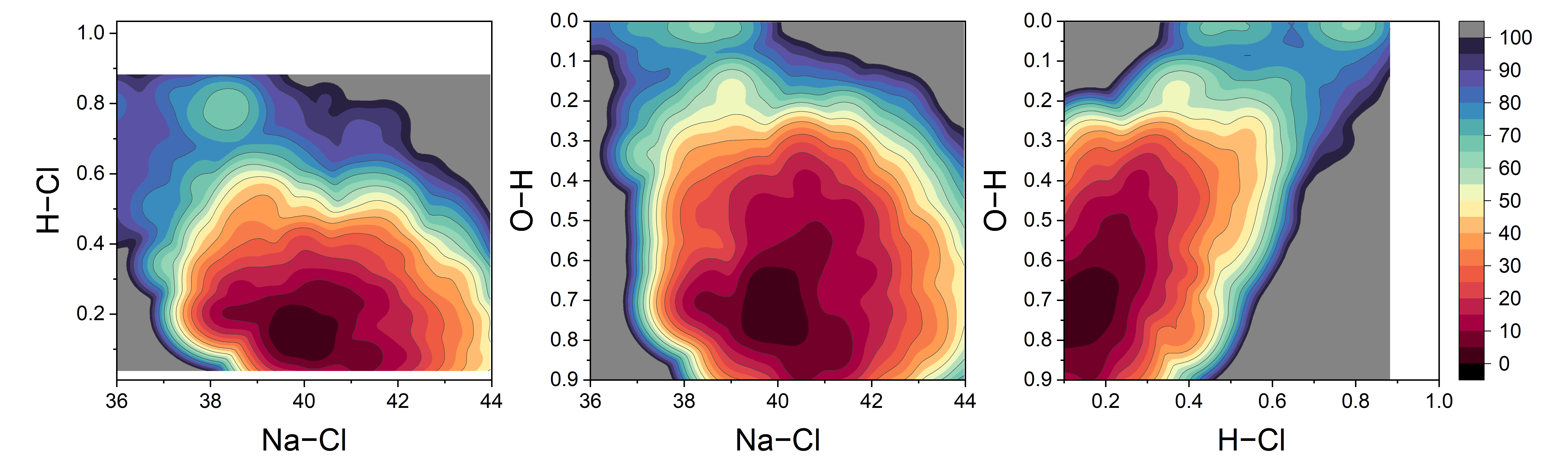


Input 3 - BARRIER 85 kJ mol^−1^ (36.5 ps)


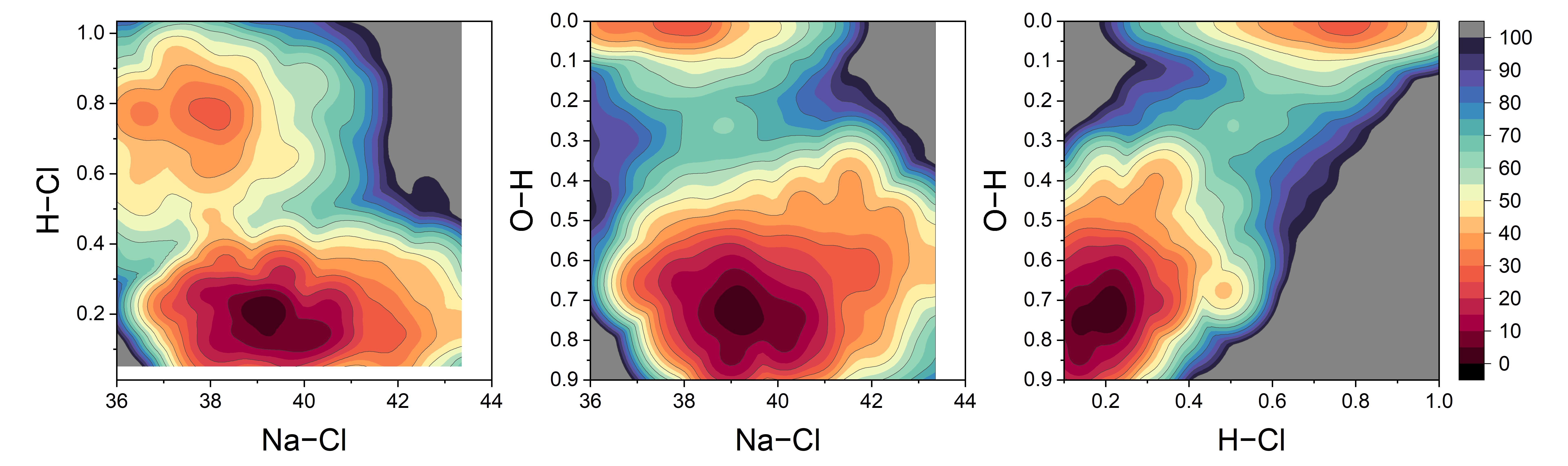


Input 3 - BARRIER 85 kJ mol^−1^ (25 ps) – without product state


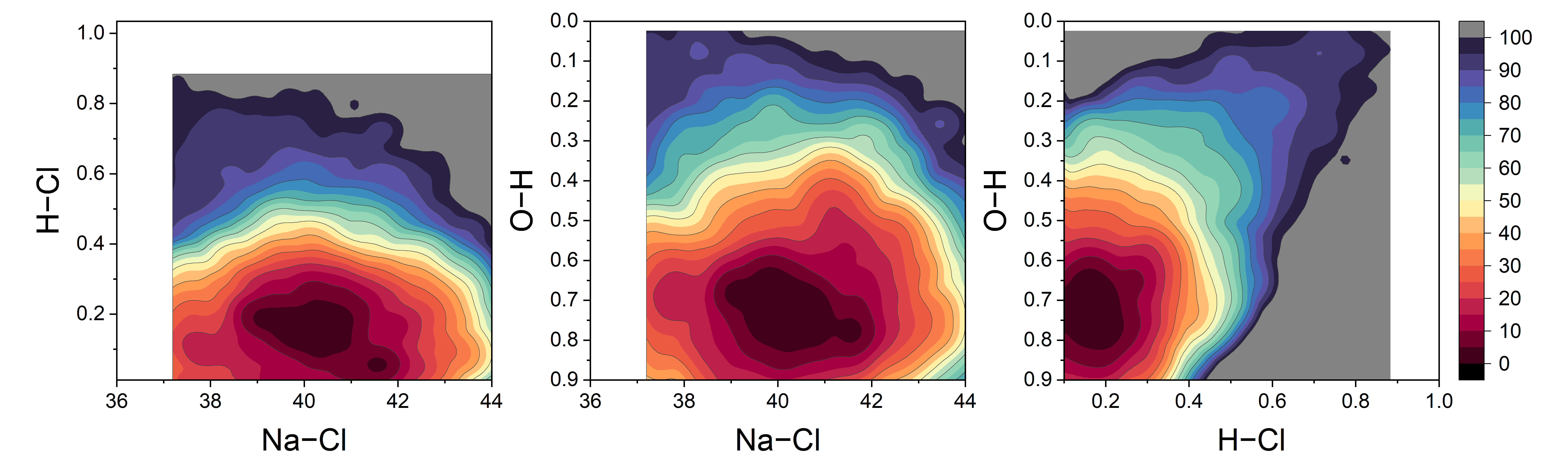


Input 3 - BARRIER 85 kJ mol^−1^ (30 ps)


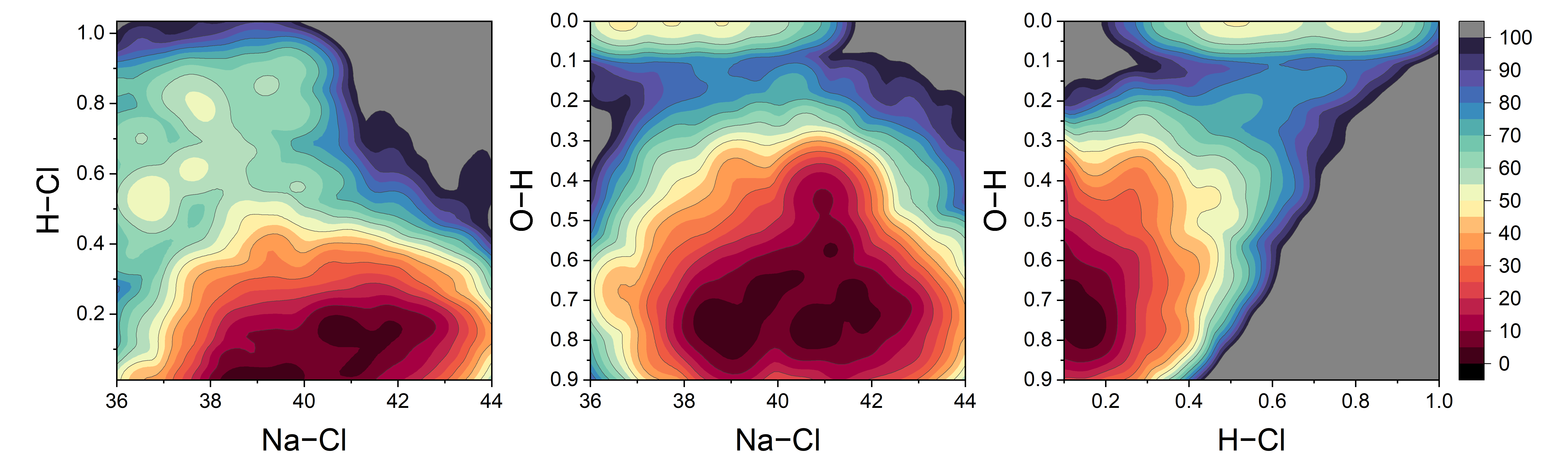


Input 1 - BARRIER 90 kJ mol^−1^ (15 ps)


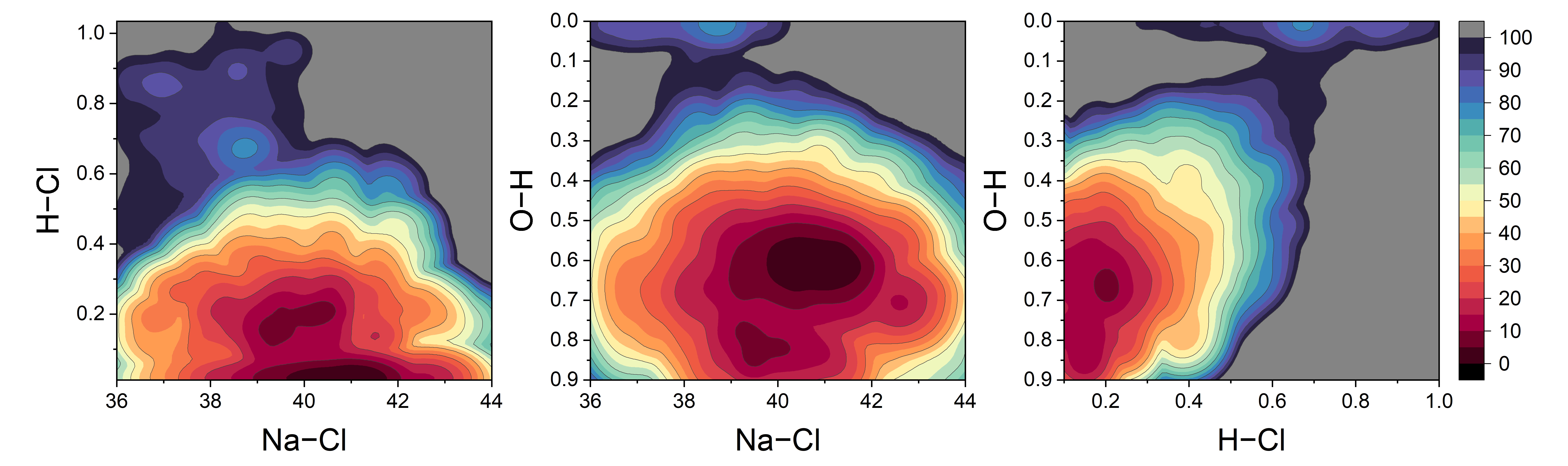


Input 1 - BARRIER 90 kJ mol^−1^ (29 ps)


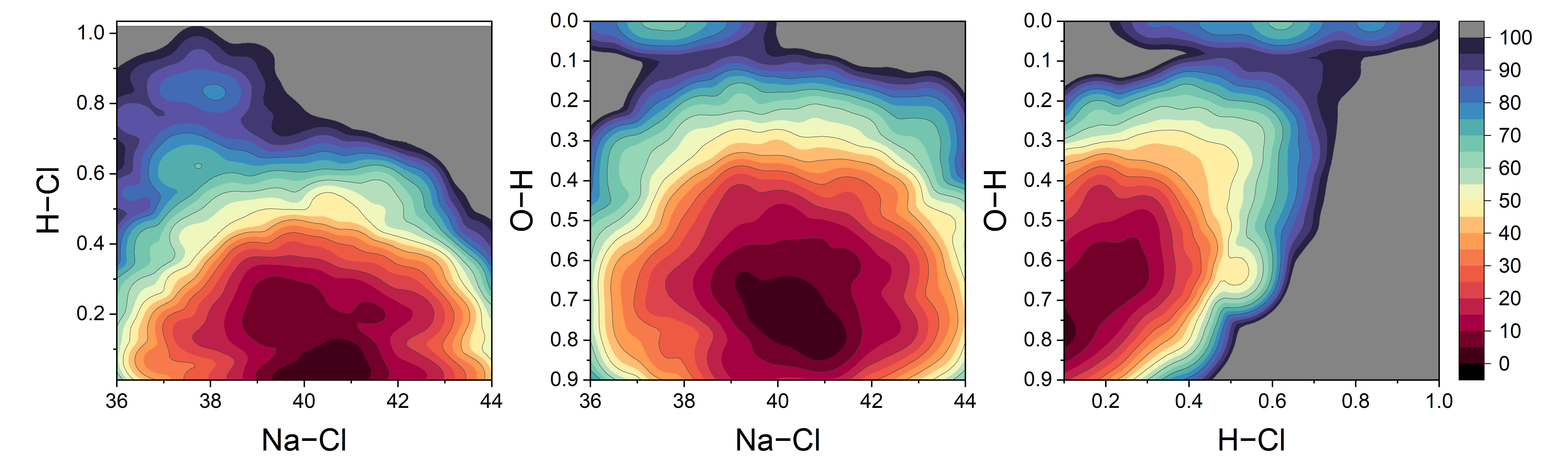


Input 2 - BARRIER 90 kJ mol^−1^ (17 ps)


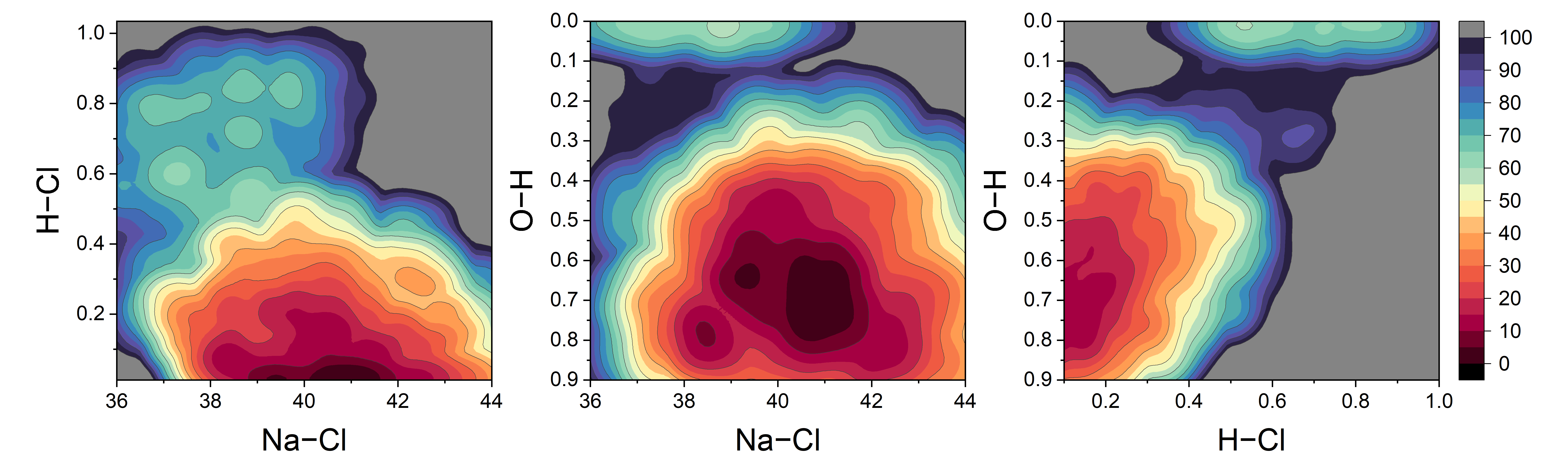


9. PES for larger NaCl Clusters

Potential energy surface for the reaction Na_62_Cl_61_^+^ + HCOOH → Na_62_Cl_60_(HCOO)^+^ + HCl, calculated at the r^2^SCAN-3c level of theory, is plotted in **Figure S16**. The reaction path we found is very similar to the one for Na_13_Cl_12_^-^ in **Figure 3**.


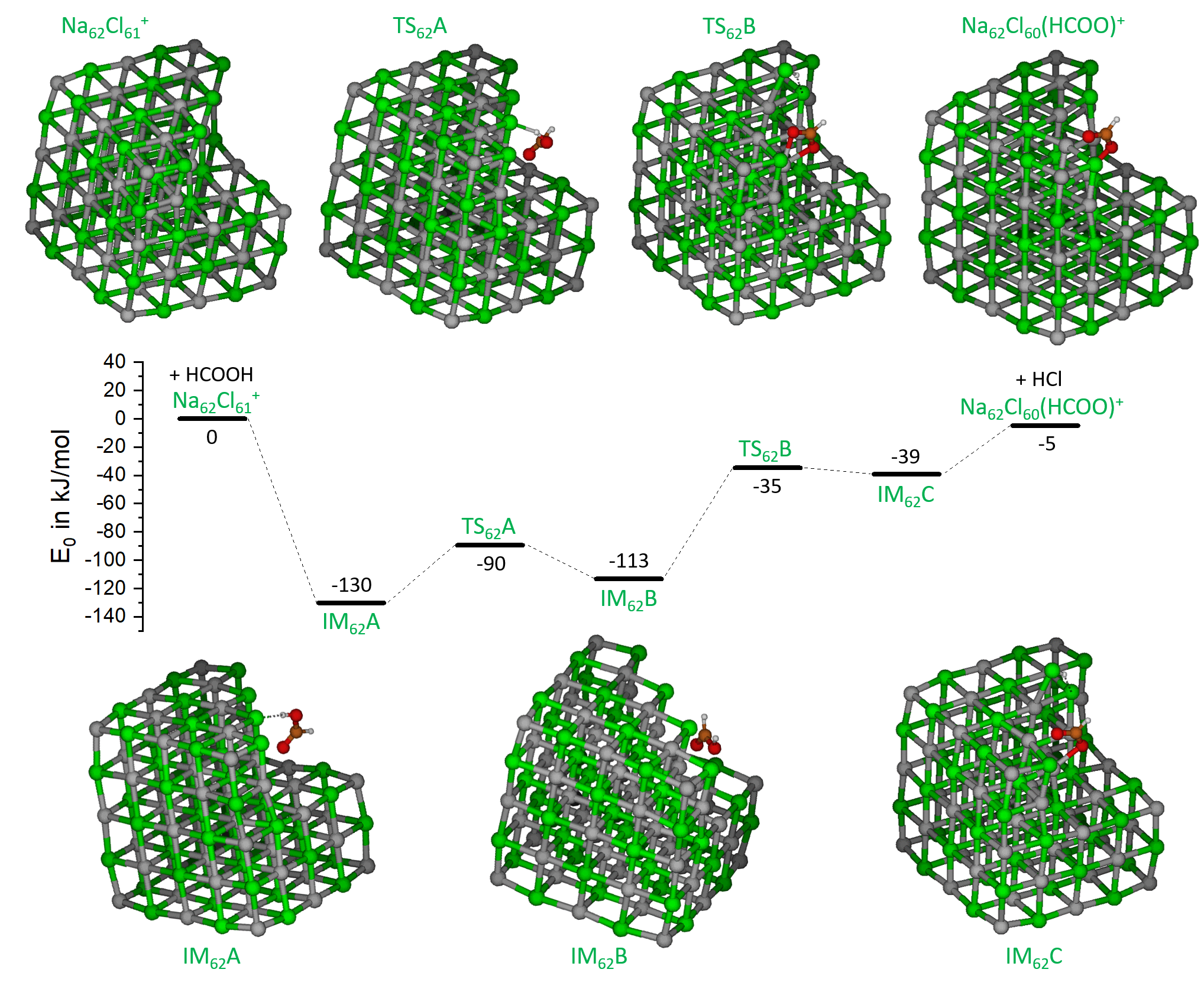


**Figure S16.** Potential energy surface for the reaction Na_62_Cl_61_^+^ + HCOOH → Na_62_Cl_60_(HCOO)^+^ + HCl, calculated at the r^2^SCAN-3c level of theory.

10. ADO, HSA and SCC rates

Input-parameters as well as the output ADO, HSA and SCC rates for **Reaction (2)** with *x* = 1-3 of the cluster Na_13_Cl_12_^+^, using the program “HSA collision rate constants vs. 2”,^[7]^ are plotted in **Figures S15 – S17**, respectively.


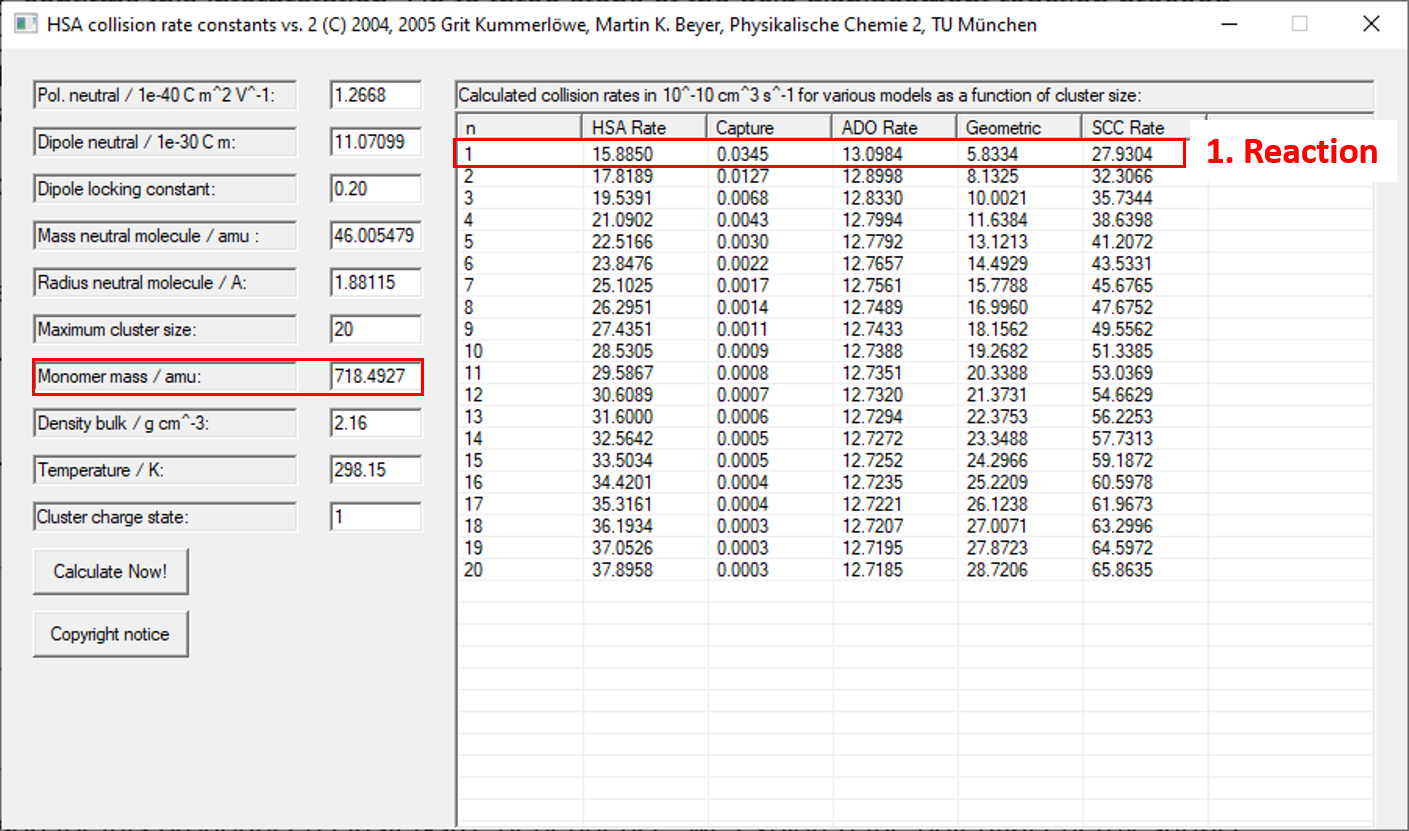


**Figure S17.** Input-parameters as well as the output ADO, HSA and SCC rates for reaction (2) with ***x* = 1** of the cluster Na_13_Cl_12_^+^, using the program “HSA collision rate constants vs. 2”. ^[7]^


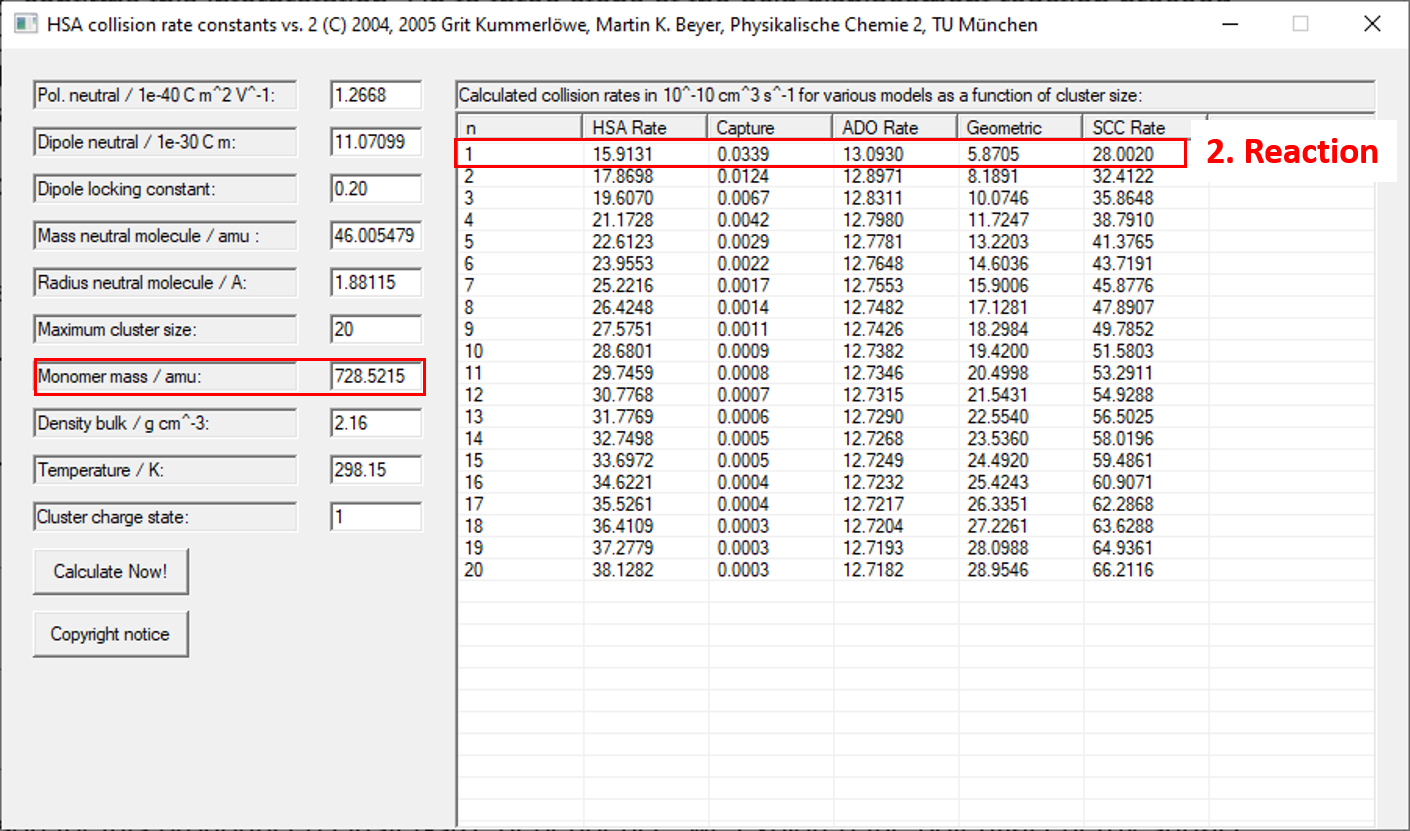


**Figure S18.** Input-parameters as well as the output ADO, HSA and SCC rates for reaction (2) with ***x* = 2** of the cluster Na_13_Cl_12_^+^, using the program “HSA collision rate constants vs. 2”. ^[7]^


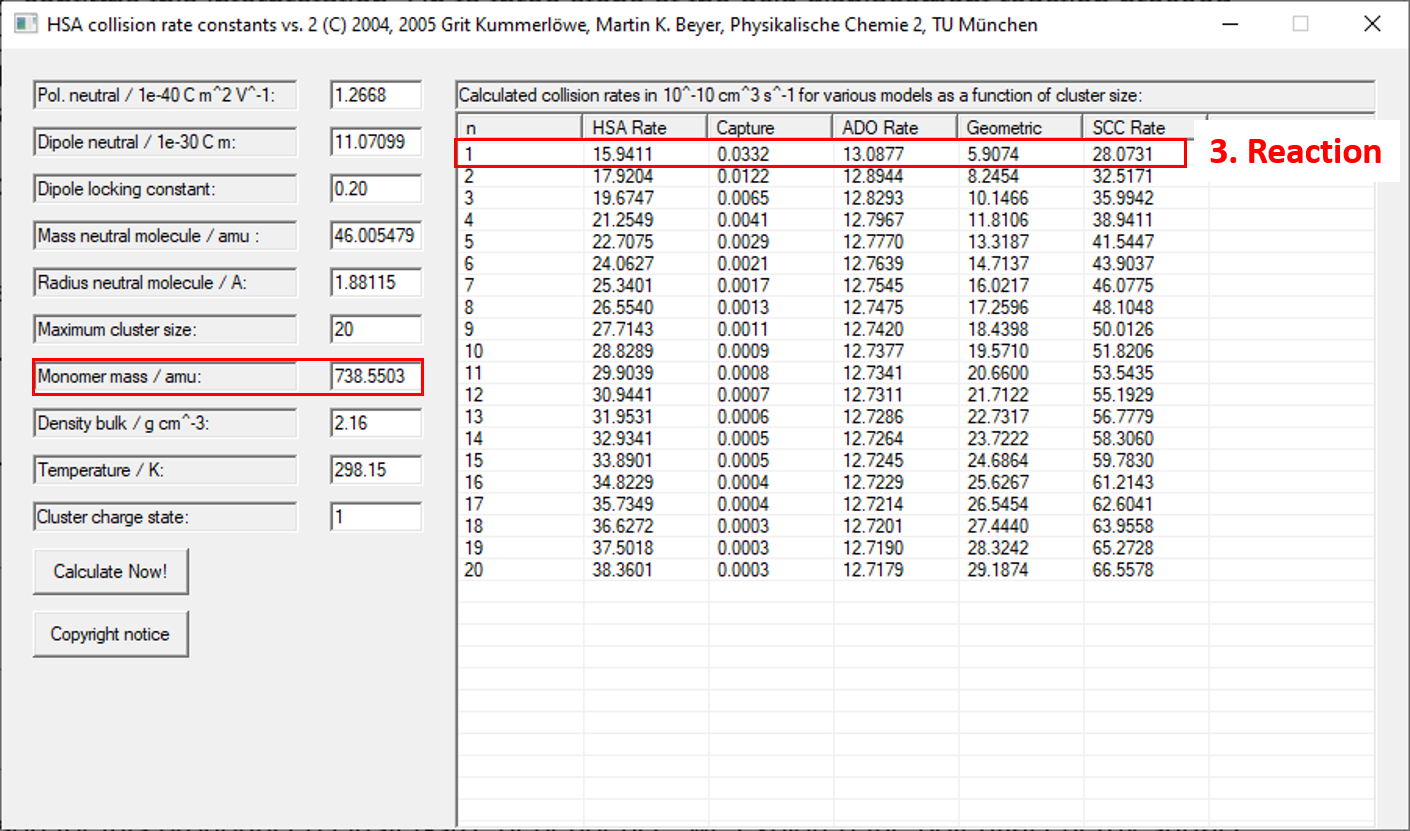


**Figure S19.** Input-parameters as well as the output ADO, HSA and SCC rates for reaction (2) with ***x* = 3** of the cluster Na_13_Cl_12_^+^, using the program “HSA collision rate constants vs. 2”. ^[7]^

11. Structures of the PES

**Figure 3 / S1 – S9: HCOOH**

C -0.13267054930527 0.40090442839708 -0.00004262574224

O 1.12048908262756 -0.09974792341178 -0.00013259867090

O -1.13946791593443 -0.25506591538158 -0.00003005283137

H -0.08848235673378 1.50189255049222 0.00001983313027

H 1.04160973934592 -1.06871214009594 -0.00018455588577

**Figure 3 / S1 – S9: HCl**

H 0.00000000000000 0.00000000000000 -1.21790647797240

Cl 0.00000000000000 0.00000000000000 0.06095447797240

**Figure S1: Na_3_Cl_2_^+^**

Na -0.82044287524557 -2.44651571528540 -0.75125926338307

Cl 1.43620505302489 -1.07625922074571 -0.51270786053555

Na 1.04598158629399 -1.54965217637048 2.06495250425804

Cl -1.44173186612048 -0.97077078500525 1.35862744522506

Na -0.23560489795283 0.92615789740684 -0.04282182556448

**Figure S1: IM_3_A**

Na -1.14151183246373 -2.19397283518940 -0.98095809487165

Cl 1.16465574507466 -0.99045747480517 -0.57128820464222

Na 0.99288077717107 -2.20067628754301 1.76426086976816

Cl -1.53093409417169 -1.47479192838915 1.52520801675767

Na -0.41450462203387 0.85241377764415 0.66313775946855

C -0.97751007693764 4.18618866940972 1.06972177277876

O -0.47211253903477 5.30318277572354 0.58024113827051

O -0.59942262101335 3.06465692902209 0.78183612944305

H -1.79029196883728 4.40049060076363 1.77724850926974

H 0.24661023224661 5.09489777336356 -0.04337689624259

**Figure S1: TS_3_A**

Na -0.49995291691322 -3.53925224062502 -1.79324954624079

Cl 1.40423390915460 -1.84072468165855 -1.12642883816330

Na 1.70079795895901 -3.50104438337664 0.90057538855486

Cl -0.93403126688213 -3.48342779671294 0.80793364384929

Na -0.57229372872441 -0.82452417764658 0.47359542996054

C -0.54443091793508 2.59956874333000 0.40415032537928

O 0.34352978846612 3.27168798662040 -0.30691448009536

O -0.60892113111570 1.38620839423403 0.47530929169591

H -1.22603184724940 3.28195956292088 0.92991163946056

H 0.93710015224022 2.64954859291440 -0.76488285440100

**Figure S1: IM_3_B**

Na -0.06903917540975 -2.57706436209711 -0.65775773275738

Cl 2.25358634925708 -1.38709710136576 -0.16427095049337

Na 1.43757019663052 -1.93437117459132 2.50204946522541

Cl -0.81919488022004 -1.07256664340352 1.36465435268165

Na 0.73411669397642 0.74825416177130 0.27401722626979

C 4.63496437715765 -2.70475072919304 2.56467317917404

O 5.02701565072245 -2.46125651616046 1.33034057175546

O 3.50263736824581 -2.55202413762380 2.99475951659638

H 5.46602778972154 -3.07178177374254 3.18277060373441

H 4.27159162991827 -2.13387472359371 0.79248076781359

**Figure S1: TS_3_B**

Na -1.69873726452358 2.59605114830354 -1.11377677855788

Cl -0.26633919195543 0.55600019667654 -1.85071747901227

Na -2.53611638643104 -0.69820983562043 -2.05504419142379

Cl -3.28072192466179 0.78877493876339 0.00616488369641

Na -1.36028789742440 -0.12368643757222 1.51498712401638

C 1.89198008845213 -0.74371031876668 1.08862458418549

O 2.26008385320783 -0.49307670269890 -0.14474416007674

O 0.76237605080264 -0.60825880807997 1.53879230960898

H 2.73459906967234 -1.10073443445726 1.69732477308593

H 1.49316360286128 -0.17314974654803 -0.68161106552252

**Figure S1: IM_3_C**

Na 3.63785710313548 -1.07635997887218 3.60637840964510

Cl 1.44976903996131 -0.24008527767872 2.57068776435348

Na 1.25545563515245 -0.02468813107320 -0.06119980390784

Na 2.74994665716373 2.07375821520889 2.33159031176738

Cl 3.00399424629921 1.87687812338679 -0.23123601327100

H 4.89629646207731 1.41953942295646 0.38638270960662

O 5.82081814283769 1.21443149599867 0.74269432972289

C 5.79787023018296 0.79638514895968 1.96857908437670

O 4.79084283602796 0.68575144836788 2.68158590902432

H 6.80208364716184 0.53837753274571 2.33637029868234

**Figure S1: TS_3_C**

Na -1.17041874515282 1.73327737348484 0.23649059147429

Cl -0.88118313366248 0.83271204774715 -2.24240940656981

Na -2.72946082841158 -0.86076477652192 -1.64623844800531

Cl -2.42882094144698 -0.50605107154747 0.95212964725833

Na -0.50843929961080 -1.78669282242978 2.10135204193670

C 1.80112293500433 -0.08238047975081 0.40836760837193

O 1.12531885663895 0.93857778681193 -0.13398353885096

O 1.30036835988201 -0.91135669531701 1.13946692583016

H 2.87942071767860 -0.08336666624378 0.18196556273820

H 0.61209207908074 0.72604530376686 -0.99714098418352

**Figure S1: IM_3_D**

Na -0.10134192030757 1.54970161535701 1.61232174162494

Cl -0.44293125912226 0.69293443487345 -2.62938178283949

Na -2.59517259936473 0.14307345221229 -1.38421803287050

Cl -2.03612089321647 -0.20533837009300 1.20263194278526

Na -0.36058841668132 -2.04169059599802 2.02797230436017

C 1.29949135348143 -0.30847127384793 0.09784667259888

O 1.04486895311003 0.91473440788317 -0.27454299876339

O 1.27023575045179 -0.63756009059072 1.28685242918114

H 1.52123515286244 -1.02410394943839 -0.71108498857948

H 0.61482487878666 0.91515636964222 -1.27782128749746

**Figure S1: TS_3_D**

Na 0.02262148516315 1.14194462809790 2.26276202278432

Cl -1.04840636702962 0.67941188745308 -3.83112390511750

Na -0.61625652897735 0.64583407388248 -1.02582384276013

Cl -2.10057483010544 0.06436323354454 1.08152688104690

Na -0.97311049538176 -2.07269958640917 2.08861512465009

C 1.47504379247681 -0.60187249666216 0.89532587809547

O 1.18009501633557 0.48919989171623 0.32813515272326

O 0.98612544543154 -0.99628961674816 1.99468928852739

H 2.21827502589938 -1.25740628956389 0.39803769049129

H -1.14381354381229 1.90751527468916 -4.19214529044109

**Figure S1: IM_3_E**

Na 2.86833274692697 -2.34531020138720 2.66137422861001

Na -1.13959945775688 -1.75528469626512 3.90762884260270

Cl 1.23979441496798 -0.48888894649843 3.56621483380757

Na 0.37191828172389 -0.68843823979540 1.04436879745289

O 1.28877835655337 -2.85951154195994 1.16816280324244

C 0.15047159793636 -3.28553150882985 1.52255225223800

O -0.82791441904091 -2.54581597712366 1.83039675458522

H -0.00036376470238 -4.38315634891565 1.55952005502781

H -0.16168116704738 0.40023614266847 5.62403530193389

Cl -1.32996358956104 0.03364631810686 6.03553013049934

**Figure S1: Na_3_Cl(HCOO)^+^**

Na 2.97843960400844 -2.44665207908375 2.47977036464772

Na -0.31970269008736 0.07614931673840 2.74842296883147

Cl 2.11962489235905 -0.06554167462430 1.79241208748826

Na 0.81079127201355 -1.58931782303860 0.03821015147935

O 1.06553695171109 -3.27777928239542 1.66329088842662

C -0.05913389914782 -2.95207538317965 2.14319985917995

O -0.71015569455340 -1.91926906522052 1.81039490439274

H -0.50831443630356 -3.62160200919613 2.90409277555388

**Figure 3 / S2 – S4: Na_13_Cl_12_^+^**

Cl 7.52066202508652 2.11914610018686 1.20904200233119

Na 7.10370235385657 2.64044512321810 3.98431067399033

Cl 4.78387322337501 0.83204243056793 3.74840346303279

Na 3.30133457600634 2.83191260759992 2.91088089146148

Cl 5.16528738056196 4.67461592645607 3.08689616111505

Na 7.23122434157836 6.32710687107144 3.07730369889343

Cl 9.00465390008318 4.60177474835389 3.83781697407950

Na 9.37512512772382 4.14897831406621 1.31105311367830

Cl 10.17903658661423 3.43928708598789 -1.28055607603248

Na 8.33140584972642 5.34178884945042 -2.04077224591094

Cl 7.70922316962676 6.09466656757642 0.40347747948924

Na 5.72199207231218 4.15668935018330 0.45485432826259

Cl 6.33846984152534 3.62278230864509 -2.28721582265113

Na 7.98014572531952 1.62743784659505 -1.38124635336838

Cl 5.95577164680159 -0.20124497758676 -1.62936646206496

Na 7.58864196522977 -2.09004895646011 -0.76285026980884

Cl 6.91722415671421 -1.85662765102388 1.77242623315109

Na 5.34201159279104 0.33308134704989 1.11269754587868

Cl 3.62347685263233 2.34952401440611 0.28754562381019

Na 4.34373625625731 1.84838505801184 -2.21156729210129

Cl 9.79171780124129 -0.43516972533134 -0.61494845183565

Na 8.95340100821931 -0.07857147499080 2.03764967429958

Cl 8.58895411620796 0.41469579454843 4.55820224334746

Na 6.49397038682852 -1.09152497934302 4.35484109743559

Na 11.60831504368001 1.27436042076089 -1.33011623048290

**Figure 3 / S2 – S4: Na_13_Cl_11_(HCOO)^+^**

Na 7.30213532327360 2.56954204685875 0.92236392579589

Cl 7.19188346464371 2.45831971614552 3.61634528123635

Na 6.18428516602281 3.11910110555424 6.07357777549270

Cl 4.18074872249348 1.37249744977811 5.85454019844088

Na 5.06257097843043 0.66817847561480 3.26945418748673

Cl 5.85730700728499 0.36618811731864 0.57208767984955

Na 3.83747488276195 -1.47920082216577 0.49417427985599

Cl 2.97797672283643 -1.14573040654691 3.02912071653494

Na 2.12967765895380 -0.29880233682875 5.44118058880347

Cl 0.41280662361742 1.60681942096454 4.61544674490014

Na 2.50729972664715 3.41068577022226 4.84891491983028

Cl 4.63915787429265 5.19689718134719 5.19354428564964

Na 5.36872377056925 4.20209136882473 2.87248963952163

Cl 3.30349967012194 2.59626026652877 2.31419406451810

Na 4.04138674813290 2.17923242462930 -0.32360514927311

Cl 2.07702066514132 0.19804971068464 -0.56119520706851

Na 1.21945881935648 0.83645947380469 2.00190992646433

Cl -0.38971035273662 2.84615069536861 0.96431624765367

Na -0.92545114723230 3.63252190113970 3.43654869274672

Cl 1.10064302303363 5.46870001648283 3.51309424925716

Na 3.44113179315064 6.71308681488877 3.30714470116758

Na 1.82152654805461 4.56488691698916 1.05281456145513

Cl 2.52142273821609 4.02417484982608 -1.43121712421305

Na 0.60050296349199 2.24348903886592 -1.44156016365652

O 5.76315874485489 4.23474020184567 0.56551017710702

C 5.00857003007409 5.24984018563899 0.47106891790065

O 4.35125102392341 5.74565372283254 1.43607617183786

H 4.90922481058894 5.72760369338674 -0.51924828929540

**Figure 3 / S5 – S9: Na_14_Cl_13_^+^**

Na 7.40074497810052 2.16941322760635 1.13038846072494

Cl 6.84913764842873 2.70490883633877 3.81800041638889

Na 4.80714181389098 1.00000121699038 3.60023055091022

Cl 5.13420034909934 0.54067719780659 0.99013485672918

Cl 8.27737456045514 -3.84955737382010 2.44191284618826

Na 6.73113307921251 -1.63630946245652 1.71750734900914

Cl 11.06139469888153 -2.70540071584841 -0.12258602865064

Na 8.81754091921483 -4.15284490889049 -0.15456922286949

Cl 8.95540219520609 -0.00103919429125 1.84730162094855

Na 13.10312130639982 -0.99863887635146 0.09642420310754

Na 12.08576289304367 -0.02667540190298 5.23455328182918

Cl 12.77547071981919 -0.54047573854346 2.70650132915311

Na 10.10838276542662 3.18169680197381 -1.28587679059655

Cl 9.63204019254945 3.85020839937587 1.25460971386927

Cl 7.91706187993776 1.68481845844270 -1.57486875149744

Na 5.82343376208430 0.02811653548348 -1.53814626652513

Cl 7.23920884652182 -2.16509702206442 -0.98107388133845

Na 9.48252923528185 -0.50471141922969 -0.81862826988321

Cl 11.73769062266061 1.14531790001030 -0.71709011405499

Na 10.51088321751184 -2.17185991255283 2.56621704226380

Na 8.42803431773082 0.50374586937721 4.51191201616643

Cl 6.17129680978725 -1.14525279773405 4.41209184064216

Na 7.80086937267706 -3.18047143967905 4.98260978010469

Cl 9.99364115941909 -1.68544774424896 5.27033800562489

Na 11.17766402593246 1.63638377694022 1.97810023672090

Cl 10.67002674606990 2.16560884379947 4.67689095110298

Na 9.09146088465654 4.15288494346851 3.85094982393172

**Figure 3 / S5 – S9: Na_14_Cl_12_(HCOO)^+^**

Na -2.12100960960926 -3.85241931272532 1.59705932700031

Na -1.13706928086393 -1.98556387274877 -1.57303968812814

Na -4.40919890891888 -0.24785105430208 -1.52988076559528

Na 4.40894945368511 -0.25395604088189 1.52969194948161

Na 2.26371367093528 -0.10893711496656 -1.57652566514316

Na 2.11583092977332 -3.85507250504132 -1.59806101797382

Na -0.03642474958989 0.07182766858385 -4.49725240898973

Na -1.29024227861188 2.00438648525712 -1.42021689417354

Na 1.13496137436640 -1.98746432425774 1.57256094092954

Na 1.29292981536401 2.00250335827531 1.42072213610986

Na -2.26369179338538 -0.10638936697712 1.57618885699693

Na 2.60070905704004 3.57530199950350 -1.52790922994127

Na -2.59635768143742 3.57824484648882 1.52864618888293

Na 0.03635590844310 0.07061644178042 4.49709843647801

Cl -3.40374575754143 -2.15200863397836 0.01801313613154

Cl 1.10805590905494 -1.99801374233888 -3.24305052435105

Cl -3.70866697740268 1.69654869358831 0.09133144243915

Cl 2.32217041061894 -0.01092501626125 3.14994197017138

Cl -2.32204145975808 -0.00711875677701 -3.14989729519900

Cl 0.00009858557452 -0.00429093950970 -0.00001689655680

Cl 3.40106162380458 -2.15650061940514 -0.01876195652774

Cl -1.11058301152213 -1.99738638318285 3.24246137572725

Cl 1.07007268538662 2.01553215439907 -3.05156792644516

Cl -1.06748388066667 2.01642167914002 3.05208830801838

Cl -0.00260813531919 -4.00242661684806 -0.00042728160815

Cl 3.71077991399960 1.69181605355306 -0.09103668867385

C 0.00282210618670 4.55580872250144 0.00078147000282

H 0.00343200229090 5.66832616162414 0.00139451668312

O 1.11977280860300 3.98129168521401 0.09546818173784

O -1.11477873050024 3.98262635029298 -0.09451599748395

**Figure 3 / S2 - S4: IM_13_A**

C 9.07642565759862 3.02937332680421 2.89578193511801

O 8.10677597448039 3.62520002925106 2.41068727161452

O 9.21910085944191 1.74764319261290 3.01682976214338

Cl 6.76811003110253 0.15468601990650 1.98340287533428

Na 4.73360073885777 -0.77200964622776 0.55108170114707

Cl 2.94581987948440 -0.44839356614520 2.48641118291680

Na 5.04885213961040 0.62596174414218 4.12747082564860

Cl 3.04265901723842 1.09627240194881 6.08926694523453

Na 1.22264960902978 0.17674412496236 4.40218127298459

Cl 0.52720035574460 2.57131522282552 3.40613840068822

Na 2.52720194147337 3.60770566644279 5.04513931996138

Cl 4.41010366624702 4.74476041149359 6.73990995024498

Na 5.01556639492290 2.21932396460674 7.46809314777521

Cl 6.91859459442324 1.81426105254674 5.72041739628480

Na 6.22791726021964 4.14495034158190 4.78875048703108

Cl 4.41173792012059 3.14686805816830 3.03281526259640

Na 2.42410559607512 2.06774263833482 1.42827505336205

Cl 4.22442695219600 1.57120222787478 -0.64190184553976

Na 6.18486701327278 2.58968781760311 1.04206429627115

Cl 5.86732176651707 5.10509959943355 -0.04198987394409

Na 3.82777005297961 4.05754793912740 -1.46898497598522

Cl 1.94987309236547 4.62288916071169 0.32799103383246

Na 0.26955231240128 5.01676002467729 2.36842360001982

Cl 2.09597373172024 6.18762014677127 3.93422764828683

Na 4.08802576711001 7.05997916892517 5.47408042751815

Cl 6.05628599447502 6.61056694024487 3.63232562332735

Na 3.95212799094124 5.62585279056963 1.96269740179169

Na 7.68786303471323 5.87410046700396 1.69010006104441

H 9.95588486234763 3.56651454518604 3.28262778698247

H 8.41183879288883 1.23574318861529 2.72037202630834

**Figure 3 / S2 - S4: TS_13_A**

C 3.88999794898276 -0.10139622844538 0.12479105951904

O 4.09589723155240 -1.09168311408221 -0.72417740648627

O 3.16276547121369 0.85543765264024 -0.13305182783948

Na 0.19460429747699 -2.45371457438044 1.12343836306725

Cl -1.92697818775793 -3.55186974008601 -0.47347993895207

Na -0.19943272963293 -3.90956409887628 -2.44745803836262

Cl -0.66024639090531 -1.55995663747044 -3.64168379790615

Na 1.32988925592644 -0.57054543408793 -2.01050330908557

Cl -0.41131175273106 0.05507593735804 0.01047838504772

Na -2.28496916899569 0.52214879925822 2.03840458780676

Cl -1.73498499767934 -1.97375057257335 3.11444311334346

Na -3.60243565683999 -2.91859482015701 1.47838357786538

Cl -4.30982011121409 -0.54956940661358 0.44930593044046

Na -2.43320832522752 -1.04379541626921 -1.54324387421546

Cl -2.92880129497440 1.49363575865023 -2.66687173865776

Na -4.61972735917437 1.87191416576000 -0.62502747209463

Cl -2.82099788628328 3.06421493332378 0.93890033046561

Na -0.94812243173622 2.53617940929259 -1.04056541046690

Cl 1.02052874120106 1.96809759388801 -3.02355337386765

Na -1.03508267324289 0.94403676497398 -4.46008668212421

Na -0.85013317093921 4.01019375963236 2.45499224883347

Cl -0.38101609189276 1.69812164564080 3.68779671895149

Na 0.27275388714938 -0.80303595457055 4.40485411733638

Cl 2.13951260202255 -1.20601872448075 2.59993241704134

Na 1.43520654376628 1.18677365775676 1.69835237237819

Cl 1.05294779299606 3.67751386695329 0.57223788323691

Na 2.77401888893803 2.89778110838291 -1.27222453124977

H 4.47966424550885 -0.14283767643451 1.05251056968556

Cl 1.85284911298106 -3.04433365552992 -1.00010735521461

H 3.44663220951142 -1.86045899945362 -0.68678691849592

**Figure 3 / S2 - S4: IM_13_B**

C 3.78885863370702 -0.00850373091805 0.17935741370509

O 3.45864332009509 -0.69283719886227 -0.90889974352988

O 3.58066772019634 1.19307465939675 0.23817728983709

Na 0.21616170593058 -2.49105953859520 1.10097436768479

Cl -1.95021327828401 -3.54782869099609 -0.47208082049157

Na -0.24097821054305 -4.02516310564089 -2.43577442272024

Cl -0.69468478022382 -1.66081964160658 -3.67278477972932

Na 1.29112860698768 -0.73600320865433 -2.08692276682975

Cl -0.30436197447015 0.04413576003659 0.00003714014877

Na -2.24510346934183 0.53417503517141 1.97970458034742

Cl -1.74880481254226 -1.95551099046273 3.08481416352756

Na -3.61907355899698 -2.88040112959584 1.45714940107667

Cl -4.28221459621728 -0.51607122892984 0.40315763059952

Na -2.39423226396736 -1.04096417418440 -1.55499209208963

Cl -2.84249869708691 1.48700480905206 -2.69484675879200

Na -4.56386157482306 1.89123695615742 -0.68959233616078

Cl -2.77460744800666 3.06740669321253 0.87396965519704

Na -0.85895804449131 2.50967829609066 -1.07340519559465

Cl 1.13534976930189 1.85343916330980 -2.99674012804960

Na -0.93651836344434 0.87309873696964 -4.41811722753477

Na -0.85751582247899 4.03639647976255 2.42582873710354

Cl -0.40234197317227 1.73349484538159 3.68739754300340

Na 0.20943070838997 -0.77610181534919 4.41851780681955

Cl 2.09539327141575 -1.19189867462713 2.64131596945497

Na 1.49186529647463 1.26124100425051 1.78099158736880

Cl 1.05958169591421 3.69359353511690 0.56600347055404

Na 2.82067087520577 2.85446146837304 -1.20426066336056

H 4.24956399321633 -0.58341491736333 0.99315245407713

Cl 1.84605594579521 -3.22281652776808 -1.03667033846659

H 3.43829432545973 -1.67014086872755 -0.75342693715606

**Figure 3 / Figure S2: TS_13_B**

C 3.21247818251582 -0.48099290434186 -0.48441770747589

O 2.56068480088719 -1.39381967745941 -1.06385368460259

O 2.94220687137140 0.75854280010091 -0.55410247496456

Na 0.19094295222405 -2.19722765151019 1.27889798488515

Cl -1.31876025955673 -3.37215141659940 -0.61889051819544

Na 1.01601314262414 -2.95966448281936 -1.97778782584527

Cl -0.24556090315547 -1.24050227312708 -3.68316558783075

Na 1.23941772104316 0.23099262781137 -2.09845214353794

Cl -0.44007193718570 0.22004933109965 0.00749512516334

Na -2.32156963080393 0.54626636448667 2.01873236164788

Cl -1.74398554255147 -1.93321123880411 3.13843478454957

Na -3.24789642121467 -2.98487067101915 1.21280277664385

Cl -4.16204902355597 -0.67492985233060 0.27666017757962

Na -2.23713342632674 -0.99148086872470 -1.71757663388445

Cl -2.85719734812959 1.55041454169145 -2.71096668220984

Na -4.52454336531120 1.77839254088975 -0.66252194282419

Cl -2.87963489683644 3.09468909312005 0.96661328116324

Na -0.99488830154224 2.77998370993216 -1.02597669755495

Cl 0.88252448744853 2.63559358417954 -3.09510740570925

Na -0.94379900251620 1.23191531741328 -4.50743140243057

Na -0.86589170647728 4.00913547259352 2.49030730899847

Cl -0.36278623301967 1.68834588971371 3.64573595291393

Na 0.28605598077415 -0.75098314482097 4.45754638838172

Cl 2.18291989735355 -1.16010794842343 2.66201686109188

Na 1.54371463203921 1.22023919204677 1.62690274408496

Cl 1.07398366402592 3.73677207188596 0.64501427036111

Na 2.77507617233950 2.94293364533364 -1.20488014332777

H 4.08231346160389 -0.77394794480931 0.13311183628776

Cl 2.21490841919001 -4.28695319936748 0.38832096707719

H 2.94252761274275 -3.22342290814135 0.45653802956377

**Figure 3 / Figure S2: IM_13_C**

C 1.32088434785773 -1.88563634321300 -2.68569496735505

O 1.65636667870361 -2.22349521841325 -1.50373987611792

Na 2.05340631312827 0.09942991474930 -1.10234738573077

O 1.25499387852403 -0.69861207078125 -3.09731886165961

Na 0.72934262557253 1.44678596428242 -3.70324769405098

Cl -1.53461907830632 2.60128639955005 -2.69077966239171

Na -2.06180458690842 0.00919819576751 -2.30502313783040

Cl -0.43713404806424 0.13196622523147 -0.06555287400692

Na 1.08891061605209 0.03602580519437 2.30352664460973

Cl 3.57276930219752 -0.10956339636751 1.01865962158324

Na 3.46120286771034 2.57945636574001 0.69904491626245

Cl 1.25073901510371 2.82336311119186 2.13270758317004

Na -1.09466542057154 2.82208204620590 3.36015023795170

Cl -1.28323731782013 0.18414650042914 3.71065351708245

Na -1.33326709424735 -2.46973961615101 3.43801814075442

Cl 0.94430571567567 -2.77323323224180 2.08132298380712

Na -0.54023029551886 -2.61001273614691 -0.21548509022869

Cl -2.02467900704992 -2.65960186086124 -2.48043947110723

Na -4.20680614142667 -2.41613424965376 -0.97466485443605

Cl -4.36312013488153 0.26375705745587 -0.94488435733949

Na -3.76183194485363 2.84959662892292 -1.19419940524939

Cl -2.57302656713435 3.05445640854515 1.17139208684486

Na -2.80483822918948 0.25183145862379 1.36344739550713

Cl -2.89851870541300 -2.54017705129365 1.32468073669943

Cl 2.20021844647249 2.65117537924122 -1.68414424929089

Na -0.19042638308231 2.89419051342472 -0.27052859104658

Na 2.92982095406115 -2.64237791450072 0.34862574395107

H 1.06739969563175 -2.69787700171888 -3.39252143666766

Cl -1.55237884878388 -0.41914712719213 -5.17219208459878

H -1.51168665343926 -1.54959115602057 -4.52548060911544

**Figure S3: TS_13_B**

C 3.08048909333201 -0.40884292452359 -0.14670776582384

O 2.33394785634172 -1.32531964114384 -0.59452292090102

O 2.90120591999467 0.83475782273245 -0.35690371626388

Na 0.58247720131230 -1.77248261082986 1.29324909837707

Cl -0.96403862444643 -3.28002939468691 -0.42132585919200

Na 0.98649483225291 -3.24654380226100 -2.26516357851735

Cl -0.17882660809220 -1.21366443930524 -3.60788536094201

Na 1.20948621323463 0.25790667077703 -1.88624124806149

Cl -0.50092519442979 0.49898626224698 0.15071727527739

Na -2.39008960890666 0.70499591409871 2.10609253544629

Cl -1.55655219027413 -1.73902296190829 3.16821007823668

Na -3.00112501795261 -2.98682384010032 1.35408676799244

Cl -4.09840161089883 -0.74134862862823 0.33928160518863

Na -2.13104447217890 -0.91906118910680 -1.57220353644436

Cl -2.83137774299553 1.52897722278761 -2.72144618001341

Na -4.54400881288451 1.67399281856724 -0.70137869740064

Cl -3.05506700008361 3.18783061598061 0.89914948372789

Na -1.06683275315645 2.93224701007901 -1.01615190169079

Cl 0.89230184396153 2.63641033958443 -3.01607116586734

Na -0.88224048225672 1.25290712641528 -4.52217296654472

Na -1.09011553856842 4.19393377215062 2.39589144432899

Cl -0.59815194033006 2.01194446610382 3.76457589081892

Na 0.18729739337007 -0.37916917775541 4.65171869275351

Cl 2.22030650973064 -0.64170906924164 3.04322104256549

Na 1.41364604695233 1.57709089939123 1.85147797016040

Cl 0.95177718000389 3.98067055412324 0.66197705679675

Na 2.71333172745450 2.98161451224277 -1.06552986581128

H 3.94517202225171 -0.69855239550073 0.47736848324261

Cl 2.78012028047367 -5.45903639968049 -1.77146872924289

H 2.69074347678833 -5.44265953260871 -0.49184393219609

**Figure S3: IM_13_C′**

C 3.32463725942449 -0.12412081773957 -0.23170192757100

O 2.79750704491573 -1.07039874902216 -0.88086597995812

O 2.97057796823609 1.09392615670072 -0.32454849483928

Na 0.58390831153999 -1.86804916709818 1.55602617425704

Cl -0.76329717990483 -3.39953611182765 -0.21851944016540

Na 1.92308897495548 -2.77596001948238 -2.10019563284105

Cl 0.12576834664617 -1.29526405438920 -3.50846467527004

Na 1.31260191484684 0.39611518106016 -1.87019181519571

Cl -0.36857088152328 0.34820577987645 0.18468575641930

Na -2.32735989220735 0.56888604270209 2.10709186662149

Cl -1.49465180085524 -1.79037904327031 3.34251826695787

Na -2.85849210041449 -3.17119749629958 1.52443349790449

Cl -3.92372540815378 -1.00879597689884 0.35636132985028

Na -1.90103672873642 -1.18977008114730 -1.54962512679689

Cl -2.74031261434158 1.21672503382347 -2.72388856393452

Na -4.50723397107603 1.33306944671943 -0.75217145935068

Cl -3.12397174954932 2.95335936460074 0.85141918959862

Na -1.12468995089932 2.77616672476392 -1.02975542009591

Cl 0.83318802726731 2.69187825991233 -3.04607391133875

Na -0.77794637792414 1.06542493658442 -4.52684728327926

Na -1.24314713025862 4.12260285432116 2.34909188483196

Cl -0.58326043873850 1.99311440612761 3.72656517594297

Na 0.28887226280301 -0.31658581206924 4.71196851819774

Cl 2.30165872035931 -0.58115831990167 3.04955704378114

Na 1.47328286111752 1.62161292368515 1.81544453866381

Cl 0.79967701401179 3.99051399673407 0.62427771369196

Na 2.64100815364861 3.21452380723534 -1.11099086481773

H 4.13883480606923 -0.36899918300032 0.47349207941201

Cl 1.55422135070451 -5.51376334079314 -1.68457772453091

H 0.49990820803685 -5.03743674190759 -1.07083871614544

**Figure S3: TS_13_C′**

C 3.32983583486023 -0.12402749216969 -0.25275811338262

O 2.79775900501207 -1.07037617804962 -0.89769792391505

O 2.97937915707151 1.09521910685720 -0.34670493464692

Na 0.61694455633548 -1.86915255516094 1.55149793216444

Cl -0.73942467905255 -3.39739766755428 -0.22375410466833

Na 1.88452958766989 -2.77815838685798 -2.09467651533291

Cl 0.10789096049462 -1.28350007847468 -3.52014545286403

Na 1.32184900889709 0.39939923643554 -1.89349861785900

Cl -0.34342614399519 0.34225557830162 0.17910948075831

Na -2.28849186768829 0.55700518342942 2.11612503293878

Cl -1.45875344414321 -1.79622280176000 3.34301555616824

Na -2.84432434548164 -3.16384114629518 1.52542742919203

Cl -3.91429104578034 -0.99581262905742 0.36679556029680

Na -1.89116675518772 -1.18792744867626 -1.54526594169689

Cl -2.76592548141079 1.22617140950190 -2.69756171682655

Na -4.57711284355104 1.36345312588111 -0.73303125241918

Cl -3.11036549164488 2.95280753723336 0.87066243503282

Na -1.10954426474283 2.77358448434358 -1.02303519903091

Cl 0.82031432155885 2.69927696555963 -3.05231420595984

Na -0.82141004895557 1.08137388437689 -4.52264385740411

Na -1.24948997801632 4.18132995937575 2.39335240076590

Cl -0.55322766628499 2.00718259737449 3.72115706388669

Na 0.31962191215661 -0.30283209062827 4.70490058997041

Cl 2.33277983819952 -0.57911904039697 3.04410541508777

Na 1.50553444339447 1.61593356111197 1.80069579482521

Cl 0.80320334024765 3.99571753834527 0.63493642443086

Na 2.63077807783344 3.22271318498820 -1.11703876184181

H 4.14574465419825 -0.36969427568122 0.45027400370264

Cl 1.56143092145664 -5.52951739007876 -1.70321782998585

H 0.50935943654900 -5.06584217227466 -1.07871069138691

**Figure S3: IM_13_C**

C 3.20794729856677 -0.15544673633980 -0.19927085455672

O 2.94549201438799 1.07391664753228 -0.37636984653124

Na 2.60212225188692 3.23959372287484 -1.05197760537793

Cl 0.76677815876059 4.03982200939526 0.69546030389847

Na 1.41443554402077 1.66417245418461 1.84839576836331

Cl 2.35505311151568 -0.50544172908660 3.01796287854560

Na 0.32677494064689 -0.34114048647667 4.66207260740347

Cl -0.62768635808234 1.97754919795737 3.75342124626149

Na -1.31075445675136 4.18140494992119 2.45685880802880

Cl -3.15031632725647 2.96947896176502 0.90198574284985

Na -1.14606406237161 2.85900577656968 -1.00722247854630

Cl 0.80763745091509 2.78854569177925 -3.01556454156240

Na -0.85483423862306 1.23749815607435 -4.53565664929972

Cl -2.80565365768438 1.34842000308271 -2.71437472679430

Na -4.60589849556220 1.41978771104783 -0.73911914122186

Cl -3.92392073627935 -0.97522697254310 0.29378420338709

Na -1.91542536862690 -1.08940189610385 -1.61067438599153

Cl -0.39449725583416 0.42903720390592 0.14422396222138

Na -2.31533374308307 0.52260930899436 2.09238138493037

Cl -1.34540320920288 -1.82974432170205 3.19789868030945

Na -2.70705791356552 -3.09073201455296 1.34711704295446

Cl -0.64989465264099 -3.32784396391212 -0.40452669952796

Na 1.56242881975411 -2.74394274882213 -1.99440913392890

Cl 1.64696851606192 -5.56586499596472 -1.91427593011151

O 2.55465865722645 -1.10783116999671 -0.72155642897726

Na 1.29333815148125 0.44793789211302 -1.92169284251212

Cl 0.05115514979621 -1.15718878334737 -3.59107141935111

Na 0.75813066164063 -1.77995639544955 1.35288908275980

H 4.05472288461510 -0.41141160255403 0.46115782378989

H 0.56291886428789 -5.29510387034602 -1.25846285141256

**Figure S4: TS_13_B**

C 3.13134093085438 -0.23424021949237 0.20233984311985

O 2.46775264387397 -1.17424879960200 -0.32045865492106

O 2.90840518793679 1.00414864303916 0.00208805993022

Na 0.61068624886374 -1.75314465452992 1.48949378369845

Cl -0.77163976729143 -3.27017560094979 -0.36587119655155

Na 1.31897726036980 -3.13737213034087 -2.03859604324878

Cl 0.16012283390344 -1.14000655870026 -3.46753689031443

Na 1.34484045669052 0.37937826636765 -1.63963345935004

Cl -0.49649721743005 0.49982190714778 0.28814109928986

Na -2.51866868322824 0.59928613502379 2.11598290684105

Cl -1.65298202861847 -1.81707554363251 3.20829713254823

Na -2.91852032124781 -3.10279330468469 1.28735546047659

Cl -4.04608792411238 -0.89184687698162 0.22577535857040

Na -1.94904193662461 -0.96019172162794 -1.55538503744945

Cl -2.67853968959494 1.46588545085346 -2.72524063505653

Na -4.52402985327566 1.51293584050293 -0.81860678857007

Cl -3.21739460106654 3.06863231257275 0.89159487173172

Na -1.09777074055071 2.92304776991237 -0.88740448246056

Cl 0.99674546255577 2.75520530825862 -2.76261854412623

Na -0.60397816155974 1.30352739772072 -4.39569546921567

Na -1.40424138445730 4.14104022191889 2.52687141353320

Cl -0.90383547892597 1.96654229666856 3.90239478249100

Na -0.06792182460294 -0.40052928872394 4.81368484248219

Cl 2.07380832388820 -0.54439109007906 3.33731214242827

Na 1.24498359567598 1.64542019811901 2.11900381234897

Cl 0.75261949224370 4.04401357731420 0.92804270333513

Na 2.66798538485491 3.15035520908912 -0.68776413560784

H 3.95707264740579 -0.49717237436410 0.88791905929866

Cl 2.24805733431830 -5.82874954314761 -2.75340782619922

H 2.96775180915148 -5.70730282765237 -3.80807810905237

**Figure S4: IM_13_C′**

C 3.11887348434426 -0.68258561341504 0.34046675065411

O 2.42280142344619 -1.61925541369260 -0.15879072607099

O 3.01688932638389 0.53542583832189 0.01032090950902

Na 0.35189229611394 -1.80427499543307 1.75031959379650

Cl -0.86479222708053 -3.39040386062780 -0.08962916544898

Na 1.63165332676594 -3.57171874347504 -1.09676420798375

Cl 0.09398050990908 -1.43119282982963 -3.47151523447746

Na 1.45375098303610 -0.05398436988270 -1.65464785409313

Cl -0.40291302988319 0.36914189391635 0.26825668488143

Na -2.51541824997014 0.72723028180767 2.00480666459474

Cl -1.90903842814708 -1.64697493689640 3.33690541029253

Na -3.12143047327826 -3.02444797757335 1.41963181228171

Cl -4.02907528598812 -0.81264734882968 0.16084131069492

Na -1.89497955422411 -1.12895633485882 -1.55653999807928

Cl -2.48055719031705 1.32565847464552 -2.83457612933690

Na -4.37684165729033 1.56054318079141 -1.01100565215484

Cl -2.99871076578231 3.13661917328473 0.63740222710152

Na -0.84011310436435 2.74592343623535 -1.05794479675052

Cl 1.29011891580184 2.32846428791207 -2.83109873725851

Na -0.41095754164974 1.01395487325136 -4.46766855969634

Na -1.18317203722136 4.21520173144515 2.26273993386128

Cl -0.89991690931154 2.09770695029736 3.79439796393570

Na -0.26996263934874 -0.24248047370829 4.91623416132891

Cl 1.91118043301421 -0.62960163773159 3.51057886275084

Na 1.26708679284402 1.53734726209596 2.15827320249343

Cl 1.03445747114830 3.83977014892475 0.77177034175967

Na 2.91464484940696 2.68686105631261 -0.73201609881552

H 3.85365535249196 -0.94631032581146 1.12177288115018

Cl 2.29913161961687 -4.05976864416749 -3.75171179995323

H 1.43574030953324 -3.08567808330921 -3.93792775096691

**Figure S4: TS_13_C′**

C 3.12026652319771 -0.68043571543024 0.35023347494656

O 2.42205674384748 -1.61573990695073 -0.14877077582003

O 3.01882261533680 0.53815171361383 0.02184591684847

Na 0.35907448205190 -1.81014694959396 1.75471775588653

Cl -0.87237020321572 -3.38902127259534 -0.08272763703666

Na 1.61173821760842 -3.54261616969621 -1.11571445269932

Cl 0.10337849049725 -1.44200182725168 -3.45214342111663

Na 1.45482643206853 -0.05186186924832 -1.64087940191917

Cl -0.40276306362062 0.36839583510798 0.27738174518390

Na -2.51255903436332 0.72910774332480 2.01461001148729

Cl -1.90354308409878 -1.64554832276491 3.34454066652332

Na -3.12404665409047 -3.01969135218490 1.43152274517822

Cl -4.03148294404183 -0.80798186116929 0.17263406662278

Na -1.89808561665165 -1.12927382085716 -1.54439882727331

Cl -2.47676233958138 1.32072299904602 -2.82747686399548

Na -4.37364892271468 1.56310569163983 -1.00381232277311

Cl -2.99434398374606 3.13868356145242 0.64361734070956

Na -0.83695689828460 2.74514102071375 -1.05119661158808

Cl 1.29325243061592 2.32523451641933 -2.82509237350881

Na -0.40560324875936 1.00171230096382 -4.45783628664812

Na -1.17808961797205 4.21658590573263 2.26884265433357

Cl -0.89446245138421 2.09993266269043 3.80132701359164

Na -0.26423571713672 -0.24106786349275 4.92220295256530

Cl 1.91541459860836 -0.63009674326258 3.51587052076748

Na 1.27111646098954 1.53652648727767 2.16324169591085

Cl 1.03808472143666 3.83955132897618 0.77633798940656

Na 2.91904667754224 2.68828285710501 -0.72649720794133

H 3.85644866744658 -0.94606321376211 1.12946876684265

Cl 2.32500483974311 -4.06383138058728 -3.75625674578601

H 1.46042387867096 -3.09575935521623 -3.95559238869863

**Figure S4: IM_13_C**

C 1.59539871254934 -2.00958830871310 -2.50074142832581

O 2.01283222575059 -2.27847340409792 -1.33389342402870

Na 2.28940500370032 0.01538107034911 -0.94682246749177

O 1.39929608473793 -0.83399840269422 -2.93541286339628

Na 1.42859742671874 1.28225856102051 -3.81388432176689

Cl -0.97024854757756 2.16129656288966 -3.04899951444104

Na -1.32005525511949 -0.47722328291930 -2.52142190550339

Cl -0.22357650051338 -0.00183619544129 -0.02212811364282

Na 1.05579764700542 0.09345926590848 2.42995264747807

Cl 3.62546234467836 -0.10799090016934 1.32167401901287

Na 3.57966351163305 2.55644081289943 0.97790815696088

Cl 1.20056624383688 2.87784222026837 2.06493666139321

Na -1.28077541785222 2.92638857310904 2.99829832299931

Cl -1.45462384344786 0.33501701348095 3.56371364937017

Na -1.45938107274221 -2.32897090008311 3.50432667631793

Cl 0.94144423325452 -2.73709270948483 2.35301992479268

Na -0.44658901846675 -2.76729109395331 0.02610122613696

Cl -1.55487862476845 -3.10181022899010 -2.34080344576707

Na -3.85854644407779 -2.69492536382369 -1.16832654033411

Cl -3.87822001937468 -0.04710957970921 -1.47997687505607

Na -3.35282355197480 2.49714879089860 -1.84687995983953

Cl -2.50498276466042 2.98260844860506 0.63758217344255

Na -2.74971004062402 0.19185010426888 1.04626404779044

Cl -2.89980843024879 -2.56858659566368 1.32228343881676

Cl 2.61201769166873 2.56345260704210 -1.55525145710456

Na 0.01017071974539 2.72931221317333 -0.47198003659433

Na 3.01046275925839 -2.65716103985757 0.68469889628671

H 1.37996599949126 -2.85306363085650 -3.17931829044262

Cl 3.67709374777713 2.55589057259743 -4.95017267189380

H 3.73810717964234 2.95959981994622 -3.72124752516973

**Figure 3 / S5 – S9: IM_14_A**

Cl 8.95134643139903 -0.15960177829116 1.90237612327379

Na 11.12937559777832 1.52820018671554 2.17865238630737

Cl 10.65083956768843 1.72096219620992 4.90679313842013

Na 9.08996441564796 3.82577717459084 4.33105216644448

Cl 9.50837433375187 3.72551549621065 1.68106142499384

Na 9.98454684464028 3.36098624382312 -0.91275993430392

Cl 11.67500712573816 1.32558159984855 -0.56694692427003

Na 9.47345878093747 -0.36987789136129 -0.81103779337173

Cl 7.28228926976839 -2.08231724970848 -1.12830366785562

Na 5.80449977391917 0.11183660087640 -1.44857088722179

Cl 7.83224091592537 1.83122701141051 -1.33342172962242

Na 7.34369884603172 2.03495601244523 1.41701749197350

Cl 5.12759463932817 0.34028472882818 1.12523777748955

Na 6.80006512040288 -1.85410757120872 1.61264450821258

Cl 6.27268999524611 -1.66802514723163 4.35653924115334

Na 7.97628924254631 -3.69850824693117 4.68457162356354

Cl 10.11309010653885 -2.17013584033126 5.11092194795795

Na 8.47643783504154 0.04710981343600 4.61834399673642

Cl 6.76070562485240 2.24128078235763 4.16757813444291

Na 4.80539861096920 0.45265681988208 3.75226274971734

Cl 8.42118523318809 -4.07872956595911 2.08358964389713

Na 10.59542500258961 -2.33950671121585 2.36234784707930

Cl 12.81291648334841 -0.67060847537465 2.64507012568149

Na 12.15675561369372 -0.45049170279814 5.22243209625991

Na 8.93634045308876 -4.08982592535084 -0.53559237114411

Cl 11.12603247292192 -2.57919763884036 -0.37128697324879

Na 13.11606960251287 -0.84059911100324 0.00378689373567

O 8.01294126912285 5.35119182984069 5.60255154796014

C 6.91979820650998 5.56837676831649 6.09031531054872

O 5.85746124861326 4.78800846601442 5.97808245563439

H 6.08882809539042 3.99781349735359 5.43472285580830

H 6.69438424086721 6.46073562744599 6.69216779374622

**Figure 3 / S5 – S9: TS_14_A**

Cl 0.19332757614973 -0.46879136546144 -0.54380183823158

Na -1.93375169715238 -2.19369579640726 -0.83442340451804

Cl -0.42333488075859 -4.37056701657680 -0.02388442663008

Na 0.22425813963720 -4.71441307640943 -2.60070459392741

Cl -1.30494830664416 -2.75598249632282 -3.51979485784255

Na -2.74144814330899 -0.62627471717674 -4.19455330689209

Cl -3.63350275319832 -0.27343550724449 -1.73717365345959

Na -1.51167714188186 1.62746051648585 -1.38359876420196

Cl 0.81347482017049 3.23202720180785 -1.03173103243915

Na 1.38206742035978 2.69097682080343 -3.59369182754876

Cl -0.75348812552723 1.14773044313464 -4.02714244159526

Na 0.82444544779727 -1.00940462553524 -3.21933154608740

Cl 3.05701876070014 0.66134994541852 -3.09033556890026

Na 2.48348952424730 1.06118743470557 -0.41017229175489

Cl 4.09728541243781 -1.13632952040635 0.27055002426158

Na 3.47369553189622 -0.69303135632308 2.81349850931517

Cl 1.28641155448828 -2.24517450764358 2.84508760170809

Na 1.79300402199151 -2.67345851406729 0.12282585033972

Cl 2.41077720772516 -3.20391085390755 -2.53602420331710

Na 4.44106979136482 -1.49925782727706 -2.33682192151354

Cl 2.16850647088604 1.54377869020974 2.26478645259497

Na -0.17274993649622 0.01600004655781 2.18025979093087

Cl -2.50266186508231 -1.53519457569804 1.88283918777351

Na -0.96079044661693 -3.65227378448892 2.49550261322092

Na 0.77332375623057 3.72011108414587 1.58602834044232

Na -4.19794533597236 0.21459441240092 0.78643173104756

O -1.17064488283253 2.10423874828098 2.89594047869780

C -1.09399282946307 3.13659903151704 3.76266607980602

O -0.31727269075373 4.05088703757429 3.61598216233297

H -1.77447974136959 3.06950149677004 4.62752244495355

Cl -2.99755738134056 2.54101080624129 0.73305915026816

H -1.93191027768347 2.23374082489223 2.20020526116642

**Figure 3 / S5 – S9: IM_14_B**

Cl 0.20487571387608 -0.48768084814165 -0.45716051070899

Na -1.89426938825627 -2.23449359987708 -0.78090126778496

Cl -0.37936153829643 -4.41915469113533 -0.06221709507479

Na 0.22604134515504 -4.67574544354862 -2.66714522632222

Cl -1.30830495436352 -2.69196008809603 -3.50756265222534

Na -2.79894235953785 -0.56360455179629 -4.05993332636315

Cl -3.69527625056447 -0.41708572128072 -1.59623429364610

Na -1.64554598450104 1.60161924234452 -1.12696169299249

Cl 0.81984856179092 3.20023546081411 -0.69453248261769

Na 1.27540933618307 2.78921541149162 -3.30197776342772

Cl -0.85812758360143 1.25952934386080 -3.78511862878632

Na 0.77979030034977 -0.92385509227983 -3.16179813230889

Cl 3.00392088996204 0.75460691878885 -3.00528992154741

Na 2.51212450314894 0.98419086611955 -0.29740467488268

Cl 4.15875098480988 -1.22169202287690 0.22292975230879

Na 3.62234006664874 -0.92837109526368 2.79873938090775

Cl 1.37974151881508 -2.36346980953995 2.82558037277737

Na 1.82804706158421 -2.71605728537865 0.08990163498195

Cl 2.39328671035139 -3.14083383726673 -2.59953452572028

Na 4.42011249514125 -1.44016353156078 -2.40879215261090

Cl 2.37478305278682 1.36116003480591 2.41431426461381

Na -0.04423094919618 -0.01748630167902 2.27063876038311

Cl -2.39452124149210 -1.58316597469754 1.98515346579207

Na -0.87622808135534 -3.73578162830235 2.48061616627245

Na 1.31337644406897 3.70343210260585 1.88639402941337

Na -4.31295915667925 -0.03954748509835 0.93584503085483

O -1.07895664766483 2.32622900594247 2.58207034914546

C -1.14887001930700 3.45501113789183 3.29504029695113

O -0.15502824697640 4.06683793422167 3.62322480535503

H -2.17286039995197 3.78374161431787 3.53842669122317

Cl -3.38860895940342 2.38065294735881 0.84922325291712

H -1.96636622352467 2.21344198725570 2.08057109312254

**Figure 3 / S5 – S9: TS_14_B′**

Cl 0.31458096340340 -0.46830129182698 -0.45273328692123

Na -1.81898512596712 -2.17132857382388 -0.69210214662148

Cl -0.29404651815021 -4.40660696550822 -0.10280868580911

Na 0.17707342981704 -4.59349161601348 -2.72862087985071

Cl -1.39338488198172 -2.57138781678909 -3.43618518529607

Na -2.85720928099297 -0.38841151386342 -3.86118063479346

Cl -3.50015479238892 -0.12257761809630 -1.31289422545353

Na -1.38485144623205 1.65559770221992 -1.01199449646262

Cl 0.95160423117938 3.25843932164836 -0.78250057646750

Na 1.35652970510020 2.82179579531083 -3.39439140813439

Cl -0.82929386845938 1.35056885708361 -3.73607667909692

Na 0.77864566614778 -0.86812205757290 -3.18468663575601

Cl 3.04070481256491 0.77197932948785 -3.08100729870975

Na 2.60085029314784 1.00282626133314 -0.36091453796578

Cl 4.24040463462988 -1.20300097937781 0.12831671751244

Na 3.70704872076225 -0.88305359771322 2.70886607799606

Cl 1.49818422734424 -2.34853676548983 2.77410191964343

Na 1.91352366928312 -2.71648789994615 0.03866367649893

Cl 2.37445921161096 -3.09720703717460 -2.68960284723726

Na 4.43835922537407 -1.43937509760456 -2.51164738011918

Cl 2.42145580895536 1.39465742679656 2.34609845135530

Na -0.02618564116532 -0.01112517396273 2.27312999348655

Cl -2.29243693820262 -1.70521753446470 2.01903577969152

Na -0.71131511792521 -3.79524828144546 2.46905113827112

Na 1.57027481483555 3.80637203468144 1.74893316274130

Na -4.42303449839051 -0.29333980664247 1.19179438711606

O -1.40229102364514 2.10598501105095 2.30350093577683

C -1.29207753976477 3.29295772682662 2.89474190245475

O -0.23577867286742 3.88117054997684 3.01274237083089

H -2.24833016588631 3.69896835916353 3.26338343329336

Cl -4.24156531201178 2.10544491179931 1.95947574354842

H -2.43275859012456 1.93605633993682 2.20751121447801

**Figure 3 / S5 – S9: IM_14_B′**

Cl 0.38381111999644 -0.47913524275858 -0.41313161924463

Na -1.82806785386554 -2.06549056785125 -0.67576336147853

Cl -0.40433133382327 -4.39791118616854 -0.14360430766221

Na 0.05393889321638 -4.52460342529617 -2.76801951187882

Cl -1.43943554320048 -2.41171832162653 -3.41025094749493

Na -2.78053543924945 -0.12420657701822 -3.72499937037691

Cl -3.36441015589144 0.16811772723250 -1.16034341569322

Na -1.10654212365525 1.79788655271213 -0.85173306047868

Cl 1.29997433795342 3.27980325955580 -0.79338613069541

Na 1.63725657173273 2.77780616810100 -3.37799210258134

Cl -0.65006005805251 1.46705202367695 -3.63415755885662

Na 0.83972789771070 -0.84084947418368 -3.14328516401188

Cl 3.19355347908958 0.64992466739610 -3.01264415048274

Na 2.70515264346949 0.93030548126921 -0.29034294547056

Cl 4.22497029864162 -1.33260319959176 0.25221934106918

Na 3.59293773598452 -0.98237917605576 2.81354024195064

Cl 1.38576832713729 -2.44408234344152 2.80566440477939

Na 1.85818464601369 -2.79839725041090 0.07888607007217

Cl 2.32894559413865 -3.15236075792448 -2.65194753872986

Na 4.47039717966317 -1.60892022734296 -2.38973456838864

Cl 2.32062884234057 1.31661315644426 2.43969824727473

Na -0.12213046108702 -0.09881761589102 2.27041067824269

Cl -2.36228028248832 -1.75841013613912 2.00623808835029

Na -0.82739157536298 -3.87235331889364 2.44039282192770

Na 1.71193599254272 3.79329653296122 1.76165177040500

Na -4.47014529197480 -0.27935799831568 1.24458603006913

O -1.60056848435933 2.18525783104441 1.83386341656571

C -1.41225934017394 3.33961999284433 2.47329916545399

O -0.34995180660527 3.92657459135670 2.49022358750526

H -2.31270594862876 3.71399429274216 2.98709166245616

Cl -4.35940643328669 1.88202228143642 2.53190718836974

H -2.61696042792591 1.94331926013663 2.01166403903320

**Figure 3 / S5 – S9: TS_14_B″**

Cl 0.31458096340340 -0.46830129182698 -0.45273328692123

Na -1.81898512596712 -2.17132857382388 -0.69210214662148

Cl -0.29404651815021 -4.40660696550822 -0.10280868580911

Na 0.17707342981704 -4.59349161601348 -2.72862087985071

Cl -1.39338488198172 -2.57138781678909 -3.43618518529607

Na -2.85720928099297 -0.38841151386342 -3.86118063479346

Cl -3.50015479238892 -0.12257761809630 -1.31289422545353

Na -1.38485144623205 1.65559770221992 -1.01199449646262

Cl 0.95160423117938 3.25843932164836 -0.78250057646750

Na 1.35652970510020 2.82179579531083 -3.39439140813439

Cl -0.82929386845938 1.35056885708361 -3.73607667909692

Na 0.77864566614778 -0.86812205757290 -3.18468663575601

Cl 3.04070481256491 0.77197932948785 -3.08100729870975

Na 2.60085029314784 1.00282626133314 -0.36091453796578

Cl 4.24040463462988 -1.20300097937781 0.12831671751244

Na 3.70704872076225 -0.88305359771322 2.70886607799606

Cl 1.49818422734424 -2.34853676548983 2.77410191964343

Na 1.91352366928312 -2.71648789994615 0.03866367649893

Cl 2.37445921161096 -3.09720703717460 -2.68960284723726

Na 4.43835922537407 -1.43937509760456 -2.51164738011918

Cl 2.42145580895536 1.39465742679656 2.34609845135530

Na -0.02618564116532 -0.01112517396273 2.27312999348655

Cl -2.29243693820262 -1.70521753446470 2.01903577969152

Na -0.71131511792521 -3.79524828144546 2.46905113827112

Na 1.57027481483555 3.80637203468144 1.74893316274130

Na -4.42303449839051 -0.29333980664247 1.19179438711606

O -1.40229102364514 2.10598501105095 2.30350093577683

C -1.29207753976477 3.29295772682662 2.89474190245475

O -0.23577867286742 3.88117054997684 3.01274237083089

H -2.24833016588631 3.69896835916353 3.26338343329336

Cl -4.24156531201178 2.10544491179931 1.95947574354842

H -2.43275859012456 1.93605633993682 2.20751121447801

**Figure 3 / S5 – S9: IM_14_B″**

Cl 0.49541949411132 -0.31455483875261 -0.39420656093957

Na -1.74774460905723 -1.87078584166880 -0.48970649711899

Cl -0.32490062900704 -4.20830452959361 -0.14340141827871

Na -0.12949027842361 -4.31463309468740 -2.81943936928276

Cl -1.65469521208790 -2.19331014409203 -3.27075033477937

Na -3.07074975367404 0.05435782981948 -3.44548652475211

Cl -3.45051162003060 0.19298943064626 -0.83730671997483

Na -1.23210820177972 1.88734273592083 -0.65756907926137

Cl 1.18258401886624 3.39082527906766 -0.60836613107849

Na 1.30103611680352 3.07504392244218 -3.26242945074492

Cl -0.96516337797117 1.69661486742651 -3.44532967716095

Na 0.60481210805497 -0.59145051265087 -3.17994345634128

Cl 2.93312202194317 0.95182246461927 -3.24699602323901

Na 2.85591658208656 1.09416936304460 -0.48661130964726

Cl 4.38875921201101 -1.24778981437424 -0.29614085904153

Na 4.20397798989842 -1.08437297993128 2.34962450857633

Cl 1.93382756462151 -2.48958833253594 2.58402415460073

Na 2.00474748937847 -2.66513600426401 -0.21252118670732

Cl 2.13509860715974 -2.92270794673591 -2.97553934870289

Na 4.27912169927868 -1.34633959088160 -2.95339609202220

Cl 3.01211049249135 1.28554507148523 2.26863632978062

Na 0.57285145314164 -0.10517735687781 2.36057258733667

Cl -1.92618463427695 -1.46052200623030 2.30519941284871

Na -0.45003216422256 -3.71552012949685 2.48784299025668

Na 1.47065231929967 3.39591635579378 2.01037177478558

Na -4.25147057820944 -0.15703152642779 1.68415114239782

O -2.16421278571296 2.50599600694592 1.54795362407904

C -1.52219496461326 2.23185125282493 2.64435854261205

O -0.29502357694848 2.10806212040807 2.68639880108693

H -2.15277105715701 2.11022874252921 3.54138367710430

Cl -4.80863663875781 2.21581954603169 2.46733051281029

H -3.22814408721647 2.49064066019542 1.78728998079782

**Figure 3 / S5: TS_14_B**

Cl 0.52234368597860 -0.42088510254043 -0.37427426340367

Na -1.77598260184892 -1.94824309198810 -0.42680396203467

Cl -0.28254697080164 -4.29034218243198 -0.22953018624533

Na -0.15397318078913 -4.28070001425284 -2.89732658451869

Cl -1.69856524426928 -2.15262238681346 -3.23271398970963

Na -3.07263869321783 0.10903429966886 -3.32444533240390

Cl -3.50666492929183 0.19048114197145 -0.73101143132293

Na -1.16282011917302 1.76307897278752 -0.44289523470152

Cl 1.23087547920834 3.33118948748136 -0.40637625795282

Na 1.28613827357805 3.11511214402152 -3.06361596673034

Cl -0.98670070623378 1.75570105349992 -3.27626086085036

Na 0.57520868330674 -0.56186192577535 -3.14359787932878

Cl 2.90313158874536 0.98956571085818 -3.20884163291193

Na 2.88340899472577 1.00939019901963 -0.43650831597958

Cl 4.43494701012410 -1.33188754485484 -0.39732328067751

Na 4.29788937162591 -1.22837366347324 2.24842646381028

Cl 2.03920904764035 -2.59198176759480 2.50924374337468

Na 2.04121749541270 -2.73671691436108 -0.27958041969473

Cl 2.10854571145823 -2.89866130712806 -3.07580522370900

Na 4.24649727710362 -1.32033492326156 -3.04182302698635

Cl 3.17100964830252 1.14643549013936 2.29223374384816

Na 0.60773309589096 -0.12519568847786 2.39128237192325

Cl -1.78342881560333 -1.67463393066991 2.39008296369565

Na -0.28011534753886 -3.86974573544927 2.40693788784987

Na 1.67662705396709 3.29381718783990 2.22386100792324

Na -3.50281696741284 0.27409475298915 1.91624761491853

O -2.06739490209772 2.01407804737256 1.71758078134294

C -1.33233743016679 2.42295804285823 2.65567502608489

O -0.12458697222755 2.11114055925430 2.83411116985270

H -1.79106897167553 3.11967633986112 3.39177255174011

Cl -5.61568250073870 2.17668960406478 1.98848270701373

H -4.88746006398164 2.60974414538496 1.02279581578370

**Figure 3 / S5: IM_14_C**

Cl 0.51934317770171 -0.31406256758788 -0.35067941387603

Na -1.86114287328471 -1.71515111301443 -0.36144034348676

Cl -0.52284233870925 -4.13641322370085 -0.17341159344242

Na -0.42406716397936 -4.14852022755897 -2.84300257533464

Cl -1.82957652206743 -1.92107683481695 -3.17065195258798

Na -3.02304233086853 0.42805607013806 -3.28470849590245

Cl -3.47707020619684 0.50807118461942 -0.68434516591433

Na -1.01310254452354 1.99958975182954 -0.37811127500237

Cl 1.49920145343235 3.37174790118770 -0.39324394455193

Na 1.50044379836825 3.15018357507633 -3.04656756722007

Cl -0.86372382090391 1.94701001506494 -3.21412662938188

Na 0.53955690235248 -0.47257764433863 -3.11269565446247

Cl 2.96094933685617 0.91743999700126 -3.22136145341584

Na 2.98143514091786 0.94272660771478 -0.44872248738192

Cl 4.37047293891284 -1.49941224368265 -0.42828946294501

Na 4.28686510185692 -1.38798362944636 2.21985256392566

Cl 1.93824025095549 -2.58104315789199 2.51835653477428

Na 1.89043829294250 -2.72949995862848 -0.26778460079240

Cl 1.91094055958537 -2.90625254052027 -3.06368563274533

Na 4.14963605771375 -1.47741854389442 -3.06857051667786

Cl 3.34255915081761 1.06228001494785 2.26664458299275

Na 0.68818264809897 -0.02233173771667 2.40906845003595

Cl -1.81469206500744 -1.39569934889434 2.43808645152335

Na -0.47043515098023 -3.69241318404308 2.46663483751443

Na 2.00911883290532 3.31099199173630 2.22420047215370

Na -3.40577915426913 0.69081151328349 2.01590398464654

O -1.82234947169905 2.25718753831740 1.82231131368183

C -1.04028840809060 2.63144257499204 2.73637240145210

O 0.15331141219129 2.25709446002571 2.88300172733289

H -1.44006771999527 3.35582396112202 3.47991006338478

Cl -6.20758519108152 0.76358392102486 1.53422152468995

H -5.63982609395203 0.68306387765431 0.37006885701349

**Figure S6: TS_14_B**

Cl 0.16988747368562 0.25456519473438 0.63024558764035

Na -2.41749303748730 1.18856263974102 0.85396322231885

Cl -1.61212871940505 3.33538399175401 2.39804403959299

Na -1.95622324709828 1.74539341122245 4.53272283316028

Cl -2.88784517196550 -0.33233159739850 3.17542768669302

Na -3.60753201234624 -2.35840140626918 1.60238718589388

Cl -3.50684867291850 -0.86174169646945 -0.57044818416732

Na -0.88004041204603 -1.80081620792119 -0.93705218822920

Cl 1.84485372898667 -2.61846493426833 -1.16786215741825

Na 1.40263550624197 -4.06580687410897 1.05501188945469

Cl -1.15720404258148 -3.38696184284009 1.33642057584223

Na -0.28913612412450 -1.26787303123947 2.92087202081352

Cl 2.33255615867909 -2.25488712274371 2.80222079452116

Na 2.78254924519688 -0.60677900514426 0.62997500124266

Cl 3.62097088188347 1.45134536570533 2.34170228194478

Na 3.98169748614136 2.94745762218603 0.18869390094656

Cl 1.51213156766501 3.88943365938624 0.02100284797481

Na 0.99308293365305 2.33613628942873 2.28352815151657

Cl 0.53504662341964 0.81332853292606 4.58483318583311

Na 2.99356341653305 -0.16783075602645 4.34882546212437

Cl 3.58712838173189 0.95347614372438 -1.48241554157513

Na 0.82672473092261 1.69512721837789 -1.65434548505533

Cl -1.85874588888622 2.62531053766214 -1.55247581026345

Na -1.05124343549129 4.56624968693737 0.09016339833494

Na 2.84415481665969 -0.99807846336650 -3.02949532447806

Na -2.99537203464880 0.53738160462161 -2.71419067368433

O -1.15679909371076 -0.74443734029065 -3.10704041167499

C -0.09736387683476 -0.59765732128923 -3.79907351088650

O 0.89103328836669 0.08690913111845 -3.46373716135831

H -0.06387694457688 -1.13678573030436 -4.76976326873471

Cl -2.74383137944809 -3.15571015999619 -3.91587175877635

H -2.03633214619700 -2.07149553984957 -3.63226558954686

**Figure S6: IM_14_C**

Cl 0.13494634823101 0.26565593818283 0.57836364974350

Na -2.42620560775081 1.25326517632542 0.83665573564760

Cl -1.58674876928862 3.34664324677337 2.43534853604790

Na -1.95680367922060 1.71236800998267 4.52997036378995

Cl -2.92566353894785 -0.31269115134173 3.12007906197704

Na -3.67139900892772 -2.29799223709024 1.49819900189282

Cl -3.54881669696602 -0.74550511995558 -0.64375423840732

Na -0.93472660432212 -1.79489221343753 -1.01289959373509

Cl 1.80315110216631 -2.58892901681861 -1.24582927600390

Na 1.31261030145323 -4.08579711200032 0.93760494481298

Cl -1.23615390051231 -3.37068009616703 1.26496302230317

Na -0.33541585645497 -1.28557726318502 2.85573488650044

Cl 2.27269904088231 -2.30954319657852 2.71544881900619

Na 2.74310139015343 -0.62566130858985 0.57178413763338

Cl 3.61415406137895 1.38319221183077 2.32136829899132

Na 3.99352910831657 2.91773913665219 0.19909251531862

Cl 1.54228567765930 3.90748802288651 0.06512761045301

Na 0.99687390120015 2.30033401367839 2.28063147546972

Cl 0.52194109714468 0.74700879783507 4.55712984361866

Na 2.96717692455353 -0.26600873099471 4.29801667419083

Cl 3.55283721294749 0.97628208117286 -1.51999924314516

Na 0.80884817479161 1.76424406579171 -1.66215727695310

Cl -1.84603272151051 2.75620282281635 -1.52508248917309

Na -1.00350896513585 4.63305838885936 0.16831451332076

Na 2.77880481265334 -0.94318917012023 -3.09524764599820

Na -2.97592185580719 0.71013231813456 -2.73810642872373

O -1.26132098209165 -0.65730992979115 -3.26303851257904

C -0.14341087724524 -0.54362038913491 -3.85893445180350

O 0.81237211871086 0.16828961578197 -3.48276728642672

H -0.01586281160447 -1.14790339474724 -4.78324315062234

Cl -2.06899587235108 -3.53430067642499 -3.15513447096297

H -1.86808052410572 -2.25783784032635 -3.44178202618372

**Figure S7: TS_14_B**

Cl 0.53694924316278 -0.39014017274227 -0.49336878040441

Na -1.78774821723136 -1.87922958855927 -0.59205634730811

Cl -0.36803137703373 -4.23643495844863 -0.19315081720704

Na -0.10146455704553 -4.37089583422490 -2.85024884614666

Cl -1.56955034521393 -2.23144144672038 -3.38423069460742

Na -2.88105878082961 0.05560760210043 -3.65134565610633

Cl -3.45201246809846 0.23896808850603 -1.09622152126740

Na -1.10830992442518 1.83908776314731 -0.74030861971470

Cl 1.35122294922961 3.33183897570236 -0.67496650596348

Na 1.52089226777382 2.97693330266781 -3.30759737311346

Cl -0.76983469555538 1.66193032422924 -3.56594257393552

Na 0.72552621749041 -0.67813156198677 -3.24408467251825

Cl 3.09388212421836 0.81218931686093 -3.27561254212992

Na 2.93768996425969 0.97303259311939 -0.51303184315959

Cl 4.42729827489083 -1.40212127873376 -0.27860486615043

Na 4.16418716659108 -1.16301254305368 2.34910918006378

Cl 1.85872565221299 -2.45836590525935 2.56629270317909

Na 1.99112721974041 -2.73353480626941 -0.21186941874997

Cl 2.19736575060411 -3.04627816196515 -2.98596243758315

Na 4.36765397545391 -1.51565500558749 -2.92728536581436

Cl 3.10987116041413 1.23999679248069 2.21587921829660

Na 0.50820639054364 0.03035018104898 2.25486554988200

Cl -1.93435552750266 -1.43345409812361 2.20653847609617

Na -0.50017549034509 -3.66704133701494 2.41732063364904

Na 1.66087233380544 3.40805040306924 1.97029290422835

Na -3.56670965284678 0.54671225340415 1.50133992539369

O -2.07291255315818 2.21541918871696 1.38604672126381

C -1.38396062015909 2.58323886524344 2.37323364820535

O -0.17589523864525 2.29059001303372 2.58160035670263

H -1.88658337867173 3.22237457601264 3.13280238256386

Cl -5.19739755113842 1.33677449081117 3.72363295127104

H -5.69546831249084 2.44264096853512 3.30693323108477

**Figure S7: IM_14_C**

Cl 0.50458594820175 -0.35373290079800 -0.52120934859628

Na -1.85025365272549 -1.78959217520776 -0.67412538771748

Cl -0.50368733944403 -4.17074102669478 -0.17394252025522

Na -0.14262271078391 -4.36772951658267 -2.81563811317696

Cl -1.53766357842031 -2.20657169787183 -3.44945674901015

Na -2.78277954431977 0.10319291131458 -3.81354806840090

Cl -3.44572175191247 0.35194796504173 -1.28591695601639

Na -1.07533013295728 1.90746506002705 -0.87591227059054

Cl 1.41297776253028 3.34386376332124 -0.74921680942715

Na 1.67150260204014 2.92917356998138 -3.36638265250425

Cl -0.63859114351955 1.66086164490620 -3.68234856321915

Na 0.78644380132720 -0.70415958856352 -3.25630075981244

Cl 3.19031592744194 0.72888383286004 -3.23142446771915

Na 2.93659240294879 0.95428903587210 -0.47937166026870

Cl 4.35806298332809 -1.45021401427545 -0.13969587529576

Na 4.00363404964830 -1.14622100658294 2.47098459299663

Cl 1.66143514360164 -2.38220926909183 2.62889995376825

Na 1.89031348893172 -2.72299370738502 -0.13566449057916

Cl 2.19023245413792 -3.10110041051644 -2.89406664606093

Na 4.39275214087987 -1.62132956972303 -2.78512729895143

Cl 3.01607083897241 1.27738330304672 2.24693649569707

Na 0.38350638410285 0.13003931729567 2.21409521037065

Cl -2.09008582733884 -1.27942614252472 2.10619965165936

Na -0.71781617046269 -3.54010617267278 2.41718155981880

Na 1.62527574619958 3.47200018504372 1.90541385114881

Na -3.65780156982952 0.73399979885607 1.29315055365537

O -2.09923017143085 2.33931480182124 1.20771150522453

C -1.44672685381622 2.71850349008932 2.21515598484244

O -0.25391290697195 2.41059352329868 2.48135245162806

H -1.96970450735347 3.38342737035208 2.93758087598794

Cl -4.51666626984458 0.79373941965363 4.03915592308531

H -5.39918354316151 1.70032920570925 4.25025902771884

**Figure S8: TS_14_B**

Cl 0.54305959349177 -0.42529641529448 -0.37549249594412

Na -1.74297591360878 -1.96250375951334 -0.45222914512343

Cl -0.26490089262237 -4.29841454698194 -0.24231189811434

Na -0.14294186314563 -4.27775671353795 -2.91685073137098

Cl -1.68940726363711 -2.14198049634164 -3.24270449934605

Na -3.08375223613112 0.11813254033508 -3.27475746596213

Cl -3.50343521373767 0.17768288210057 -0.67947087149411

Na -1.12799114762094 1.76510545540691 -0.40899322568140

Cl 1.24842342185704 3.33998639341556 -0.40085425898361

Na 1.28566978204132 3.12353686021839 -3.05733889337081

Cl -0.98980393563146 1.76239805308530 -3.24488275985815

Na 0.58372763299528 -0.54913952078363 -3.14539493542701

Cl 2.91014683517406 1.00120167992433 -3.20546050098250

Na 2.89581720117337 1.01681745222120 -0.43037942755687

Cl 4.44608117834879 -1.32707816470901 -0.39145870856604

Na 4.29654832769892 -1.23056323450387 2.25718302034821

Cl 2.03524258000585 -2.59861782006967 2.50755682334265

Na 2.05784557766590 -2.74139037677663 -0.28357718427152

Cl 2.11902496541068 -2.88902399849862 -3.08192268244760

Na 4.25674502226647 -1.30875419380668 -3.03867376699270

Cl 3.16647617837763 1.14485783711497 2.30341690669671

Na 0.61082208719780 -0.13479764358680 2.38770759771626

Cl -1.80607500993543 -1.67644768751583 2.38659326163230

Na -0.29464361258028 -3.86761341136392 2.40029186638260

Na 1.66356279319849 3.29140971115658 2.23325611196990

Na -3.66937277178412 0.22875155980883 2.02816055345847

O -2.12255448986193 1.96266579379199 1.76353865828226

C -1.36514388274341 2.37041419933437 2.68305188173243

O -0.13841019971446 2.10069283272081 2.79949098068247

H -1.81637959895348 3.02263924763300 3.46362395561042

Cl -5.63168089024387 2.38786912338769 1.77324781154091

H -4.72972625495132 2.61521636162844 0.88563102209776

**Figure S8: IM_14_C**

Cl 0.54302228114481 -0.30556687429022 -0.35426566127300

Na -1.84361455769289 -1.69300473613839 -0.39640003013774

Cl -0.54160579194988 -4.12018308779472 -0.19317598079451

Na -0.43783220564909 -4.12350518463986 -2.86819652995076

Cl -1.81865380436430 -1.87497526456596 -3.19410938821521

Na -3.00111986903458 0.48812906264681 -3.25720394400894

Cl -3.45899640350621 0.54468476532929 -0.66002306350143

Na -0.95153836433865 2.03233660505966 -0.34365268888795

Cl 1.56384104179058 3.38271573285048 -0.38849710649930

Na 1.55357707857262 3.15890063543616 -3.03952602681672

Cl -0.82386761310123 1.98312916923924 -3.18789822204259

Na 0.56211215464052 -0.44770702156981 -3.11636948260206

Cl 2.99786239309430 0.91479519305099 -3.21431538527238

Na 3.01251424180806 0.93558069751713 -0.43980308270557

Cl 4.37600651727614 -1.52198461391898 -0.41521292887435

Na 4.27523345439392 -1.41919286340144 2.23552216823027

Cl 1.91157002035051 -2.59460697064756 2.51585382216490

Na 1.88622801690018 -2.73548807184197 -0.27272391727370

Cl 1.91046154182200 -2.89887530033195 -3.06886101412238

Na 4.16393553472483 -1.49182047327544 -3.05824114906207

Cl 3.35172609829687 1.04141060082846 2.28353363849034

Na 0.69709484810501 -0.02863662718213 2.40304138238584

Cl -1.84398964028636 -1.35648953973694 2.42537375249234

Na -0.52250795429536 -3.66697500357315 2.45370348336064

Na 2.03308614488136 3.30375804361599 2.23310093461298

Na -3.54422814928242 0.73046879083286 2.12126505249584

O -1.86449034061254 2.25541958409733 1.87383925948841

C -1.04931042246229 2.60122669049935 2.76867181232908

O 0.16169169433921 2.25642042993860 2.83420799027289

H -1.42965761736520 3.26711368133413 3.57494299667006

Cl -6.34088338987888 0.66657418199360 1.34835333074608

H -5.63967993832104 0.60921576863845 0.25473797830103

**Figure S9: TS_14_B**

Cl 0.52234368597860 -0.42088510254043 -0.37427426340367

Na -1.77598260184892 -1.94824309198810 -0.42680396203467

Cl -0.28254697080164 -4.29034218243198 -0.22953018624533

Na -0.15397318078913 -4.28070001425284 -2.89732658451869

Cl -1.69856524426928 -2.15262238681346 -3.23271398970963

Na -3.07263869321783 0.10903429966886 -3.32444533240390

Cl -3.50666492929183 0.19048114197145 -0.73101143132293

Na -1.16282011917302 1.76307897278752 -0.44289523470152

Cl 1.23087547920834 3.33118948748136 -0.40637625795282

Na 1.28613827357805 3.11511214402152 -3.06361596673034

Cl -0.98670070623378 1.75570105349992 -3.27626086085036

Na 0.57520868330674 -0.56186192577535 -3.14359787932878

Cl 2.90313158874536 0.98956571085818 -3.20884163291193

Na 2.88340899472577 1.00939019901963 -0.43650831597958

Cl 4.43494701012410 -1.33188754485484 -0.39732328067751

Na 4.29788937162591 -1.22837366347324 2.24842646381028

Cl 2.03920904764035 -2.59198176759480 2.50924374337468

Na 2.04121749541270 -2.73671691436108 -0.27958041969473

Cl 2.10854571145823 -2.89866130712806 -3.07580522370900

Na 4.24649727710362 -1.32033492326156 -3.04182302698635

Cl 3.17100964830252 1.14643549013936 2.29223374384816

Na 0.60773309589096 -0.12519568847786 2.39128237192325

Cl -1.78342881560333 -1.67463393066991 2.39008296369565

Na -0.28011534753886 -3.86974573544927 2.40693788784987

Na 1.67662705396709 3.29381718783990 2.22386100792324

Na -3.50281696741284 0.27409475298915 1.91624761491853

O -2.06739490209772 2.01407804737256 1.71758078134294

C -1.33233743016679 2.42295804285823 2.65567502608489

O -0.12458697222755 2.11114055925430 2.83411116985270

H -1.79106897167553 3.11967633986112 3.39177255174011

Cl -5.61568250073870 2.17668960406478 1.98848270701373

H -4.88746006398164 2.60974414538496 1.02279581578370

**Figure S9: IM_14_C**

Cl 0.60456899075926 -0.33021088189225 -0.43048913073441

Na -1.77379179956707 -1.72501379434660 -0.27239784996232

Cl -0.43862246080826 -4.12988605800877 0.03572497759273

Na -0.46995054562409 -4.30647707600261 -2.64022737707270

Cl -1.88878652306283 -2.10270039257625 -3.05227830402175

Na -3.13159911515026 0.24412491887683 -3.16747175732278

Cl -3.40507667424341 0.46940237451797 -0.57063865714922

Na -0.94126019997383 1.97938014341920 -0.51031157179614

Cl 1.55957738846725 3.35873241684518 -0.77006312355396

Na 1.41334763065383 2.95268418074596 -3.39672218282184

Cl -0.94699047898716 1.74606435207988 -3.35661281590249

Na 0.47402250966027 -0.65307907119482 -3.18039799041708

Cl 2.89159261670564 0.72163706616181 -3.48911586758109

Na 3.04050518164029 0.92875759917296 -0.72630749042255

Cl 4.43886094917219 -1.51368343022075 -0.60173009382459

Na 4.45646572709991 -1.22678935512099 2.03597379330052

Cl 2.12634133574121 -2.40372958191482 2.51798286134231

Na 1.96948877823511 -2.73338296637896 -0.25587981325151

Cl 1.85834556072276 -3.08692751724107 -3.03255229599847

Na 4.09035321092158 -1.65891719461907 -3.22729951570372

Cl 3.51091645946434 1.22474443485184 1.96483761472124

Na 0.87100979270027 0.14068729609729 2.29237336975215

Cl -1.65232469216009 -1.20250947841842 2.54338067381688

Na -0.29877910888085 -3.49207268654737 2.64042789361335

Na 2.15053467667485 3.46143030233014 1.82340652496003

Na -3.40884694676893 0.84067112220025 2.08202895512470

O -1.73632424536687 2.36733195262165 1.74832818130067

C -0.88562927943227 2.76214410256765 2.58663709171827

O 0.33400104261551 2.44082144916607 2.60730057565339

H -1.23783275268302 3.45412472507744 3.38406381846989

Cl -6.41050636809144 0.62253404551693 1.72013665474544

H -7.08300166043389 0.69789900223373 2.80929685142504

**Figure S16: Na_62_Cl_61_^+^**

Na -0.01493871831540 -0.01464386452165 0.04774174449024

Cl -0.10579904671512 -2.88764312843772 0.19514124311796

Na -0.07941998130834 -5.60121004438319 -0.14081210880245

Cl -2.79738588438984 -5.62777724913139 -0.06245168490279

Na -5.47190616801232 -5.47154962941601 -0.17609096542660

Cl -5.62811381893451 -2.79702533725831 -0.06251765214493

Cl -5.57470086123731 -5.57440383186245 -2.85276473764124

Na -5.35396482358704 -5.35373132965261 -5.48768054996776

Cl -2.71601448487946 -5.54807296289162 -5.62769647623954

Cl -5.54827555127060 -2.71578087927526 -5.62775932930337

Na 0.00624689406523 -5.46936272774796 -5.53790953858726

Cl -0.00575612349652 -2.75772882426155 -5.61850878738143

Cl -0.01347162680806 -5.63834862250086 -2.80884454152976

Na -5.46953073905855 0.00648214941090 -5.53802536026765

Cl -2.75789871844985 -0.00555329727529 -5.61856706104314

Cl -5.63856855076355 -0.01317355325438 -2.80895969644699

Cl 2.71406620891633 -5.56772717170271 -5.54705414172137

Na 5.34869674574387 -5.36951196067938 -5.35958930111207

Cl 5.52967644858414 -2.73576495319447 -5.54142325617841

Cl 5.50439931378071 -5.57623679634228 -2.72109912141706

Na 5.51560865431127 -5.45406651663322 -0.02532442630538

Cl 2.66611406567118 -5.68913474575342 0.11936570571409

Cl 5.60904552315944 -2.75222851162663 -0.03553822431748

Na 5.45146268544073 -0.00539723349801 -5.45435542822532

Cl 2.72905176114702 -0.02207476727962 -5.58148704156319

Cl 5.59429978340971 -0.01352371086110 -2.74820280247135

Cl -5.56786383183365 2.71430019324512 -5.54723420826929

Na -5.36962707112274 5.34893241079897 -5.35982110976322

Cl -2.73587246942365 5.52988255264817 -5.54160251077037

Cl -5.57640472896097 5.50469201622949 -2.72133830496286

Na -5.45430015330236 5.51596461823530 -0.02555831194029

Cl -2.75245744399240 5.60938599923820 -0.03572861904560

Cl -5.68941543527525 2.66647321524681 0.11918551249118

Na -0.00550343838970 5.45163844866411 -5.45447694443118

Cl -0.02221211155823 2.72922311508030 -5.58155926915185

Cl -0.01368875292378 5.59453579406443 -2.74833127579379

Cl 2.70737032274910 5.53199339645661 -5.54456975908937

Na 5.34243499192873 5.34254452429890 -5.34154871939051

Cl 5.53185763275463 2.70747245751178 -5.54451042194169

Cl 5.54014366116627 5.54030862161824 -2.71430492904557

Na 5.45418582847571 5.45441558398361 0.02138596204078

Cl 2.74202442402137 5.59892053852344 0.00263922938970

Cl 5.59866436774888 2.74225016199845 0.00270399911199

Cl 0.09520469841819 -2.85107066603527 5.50714005970089

Cl -2.85149128262455 0.09568066244207 5.50706782093247

Cl 3.16051639576633 -5.71995627503526 5.43816011211185

Na 5.70721926340277 -5.32582830428536 5.32143243149948

Cl 5.66802292134324 -2.69152063818889 5.51636308553849

Cl 5.93299218494031 -5.49125253005097 2.61938827174393

Na 5.41258203267336 -0.01674139406580 5.49125682311260

Cl 2.72027197318240 -0.06686276727754 5.61942825257199

Cl 5.64079340814080 0.01632785252618 2.73967821529526

Cl -5.72031095531563 3.16099352773274 5.43797543806181

Na -5.32617537897849 5.70769201919837 5.32119453191954

Cl -2.69186722616117 5.66848848874799 5.51617793577703

Cl -5.49153157074151 5.93339912254369 2.61914462961492

Na -0.01708871323908 5.41301802206587 5.49112584588906

Cl -0.06724785362215 2.72070714995548 5.61936877380642

Cl 0.01604519735733 5.64115928594145 2.73954847611644

Cl 2.69689178378334 5.51316505877606 5.56521617383941

Na 5.31693430497714 5.31728131006625 5.37805900965103

Cl 5.51277771123303 2.69724082145033 5.56528537828597

Cl 5.54707959480393 5.54735987358977 2.73738114508590

Na 0.00077053519789 5.52329566241466 0.01145550903308

Na -5.60148039979603 -0.07906471122751 -0.14091925170328

Na 5.52300497490875 0.00100166964270 0.01157922201601

Na -0.00866024367380 -0.00848821954827 -5.50681809692788

Na -0.17606897474485 -0.17564405576988 5.69923511428721

Cl -0.04301703958018 2.74740286084127 0.01882775492488

Cl -2.88794086516328 -0.10546461220826 0.19509056789759

Cl 2.74710615081896 -0.04276370169921 0.01890427876693

Na 2.82965145917190 -0.02343050849145 2.86505710396526

Cl -0.04879721067927 -0.04856297140412 -2.71819372141526

Cl 0.01622823898132 0.01659630580027 2.76140526469849

Na -2.76589622019217 -2.76569191174065 -5.59889067154952

Cl -2.78268808212739 -2.78242310508706 -2.81613397398386

Na -2.78136081514866 -5.56649554743367 -2.86019407715838

Na -5.56676124278903 -2.78106103491829 -2.86025770867174

Na -2.78420776704581 2.76135528991571 -5.51617392854809

Cl -2.81277164174449 2.72316369680558 -2.72453356241597

Na -2.77436095268309 5.52391434406433 -2.76985317758172

Na -5.57653497442251 2.75892010767811 -2.75591523581626

Na 2.76114994186490 -2.78406776222967 -5.51604189896841

Cl 2.72289437741453 -2.81257631821147 -2.72440257622846

Na 2.75862410822373 -5.57633999971966 -2.75573943979360

Na 5.52364050322826 -2.77419308531452 -2.76967061777358

Na 2.75623977674745 2.75637697175880 -5.51449909821433

Cl 2.74350899156048 2.74370340015160 -2.74963635340904

Na 2.76173472294058 5.52337150678995 -2.75346249777956

Na 5.52317587488263 2.76190089801412 -2.75340393829667

Na -2.99578705114755 2.76859155455261 5.63315767961033

Cl -2.85359419540786 2.89433999383927 2.74066768916618

Na -2.72637905644903 5.68338902940847 2.77261595470816

Na -5.57889853950118 3.23977486484572 2.84531279077039

Na 2.76810958934820 -2.99543409126492 5.63328403457858

Cl 2.89392762871550 -2.85329768599966 2.74083508234348

Na 3.23936318305402 -5.57860618771147 2.84549106713234

Na 5.68297780983187 -2.72609327541419 2.77280058412423

Na 2.69562132140296 2.69600620124982 5.57545216489473

Cl 2.75223952680773 2.75255754087106 2.77560963041906

Na 5.52301757858392 2.76880245159181 2.79646594887984

Na 2.76851515663613 5.52333717436849 2.79639988365527

Na -2.86429634658842 -2.86396500968877 -0.10388987406697

Na -2.81740176689533 2.80896063219934 0.03559805480903

Na 2.80862097249909 -2.81714460744867 0.03574473532616

Na 2.77646600958907 2.77672530587536 0.03252798473718

Na -0.01910358738917 -2.81829970023857 -2.79375580521884

Na -0.01750213097062 2.78401452259674 -2.76142988918511

Na -2.81853077384096 -0.01883637290408 -2.79381346511332

Na 2.78377620529269 -0.01731032344423 -2.76135410379123

Na 0.22896195627714 -2.64977818140783 2.96473709262423

Na -0.02374373547664 2.83001425160054 2.86499689792990

Na -2.65015324892716 0.22937897574071 2.96467168199441

**Figure S16: IM_62_A**

Na -0.03186047752795 0.07977131500245 0.03367613462126

Cl -0.11235239562202 -2.77481422689129 0.14966487502546

Na -0.18796625695540 -5.48999328004393 -0.02395245275426

Cl -2.89755423456848 -5.47163688167081 0.22205610957194

Na -5.56714579269792 -5.27767285423168 0.02711536479438

Cl -5.68417508089389 -2.60916769938561 0.14890686317430

Cl -5.69209915316132 -5.45021356248399 -2.62537980310924

Na -5.49337523010997 -5.28389229803084 -5.26897667229700

Cl -2.86619861300920 -5.51606674641040 -5.41994202156516

Cl -5.64815912197879 -2.65190773889937 -5.46004373840172

Na -0.14419814534173 -5.47133530055792 -5.40311458054224

Cl -0.11085460128702 -2.76088972755653 -5.53340508445383

Cl -0.14850499424434 -5.61305549461562 -2.67916705311223

Na -5.52575252093964 0.07147397858441 -5.47450994505283

Cl -2.81513630127375 0.02339990749455 -5.56808186678223

Cl -5.69772179218552 0.10584955521250 -2.75490178985088

Cl 2.56723856595331 -5.60590758755013 -5.44574396349553

Na 5.20488522086819 -5.44004052911307 -5.26014796895741

Cl 5.43746166358179 -2.81427723905389 -5.47941903346127

Cl 5.37429339020465 -5.62190683025331 -2.62096224795520

Na 5.37479452136252 -5.47645427951908 0.07715671054265

Cl 2.54058271879001 -5.67043840251141 0.20467086862211

Cl 5.51457934764590 -2.77498278898508 0.04065207395365

Na 5.40245718311794 -0.08119748447339 -5.42136767545653

Cl 2.68215424936011 -0.05591703529047 -5.56562832930187

Cl 5.55863657358668 -0.05853990484077 -2.71411427737404

Cl -5.58334046479162 2.78636401046587 -5.54428830309219

Na -5.34497013016173 5.41890179664236 -5.38116004227929

Cl -2.71099134729646 5.57301589085923 -5.57044662594277

Cl -5.55137938148397 5.62102776034762 -2.74477691794512

Na -5.44020918896599 5.64057313995087 -0.04624711466945

Cl -2.73368492517494 5.70350182928514 -0.04790735174418

Cl -5.71262080390077 2.82112194374030 0.11230008292631

Na 0.01950769780931 5.45957816701664 -5.48113324251379

Cl -0.03260681533718 2.73875525233332 -5.59642211322256

Cl 0.01319749014027 5.64676972068978 -2.77466746849287

Cl 2.73695395980602 5.50371769772814 -5.56268116429558

Na 5.36675192623364 5.27287013598740 -5.34860345685368

Cl 5.52366546581266 2.63467103235420 -5.53282860021038

Cl 5.56886268470259 5.49833062388626 -2.72236456323132

Na 5.47865877963910 5.44074169263804 0.01664990393058

Cl 2.76912666054838 5.63499469790530 -0.00902523353578

Cl 5.59765900971525 2.72721741567929 0.02185319726100

Cl -0.13749010080921 -2.73356512788464 5.46581358086019

Cl -2.91648795343886 0.11980354679026 5.46734091465269

Cl 2.90893886439089 -5.66086294395582 5.51610827550273

Na 5.46833613910208 -5.31672922688193 5.42675339437502

Cl 5.47536322997062 -2.68276638901834 5.58857789462616

Cl 5.73323610579533 -5.49925471130762 2.73386456448673

Na 5.33040823239171 -0.00482931384486 5.53636720310518

Cl 2.63352460944100 -0.00867695690022 5.65077216068426

Cl 5.57679132136367 0.00954413847742 2.78779383561811

Cl -5.72643340507883 3.23770314134756 5.42550426875381

Na -5.33577448920064 5.79360065546528 5.30578463213509

Cl -2.70077415938114 5.73910958720586 5.49654235417852

Cl -5.47992385304426 6.02428901357039 2.61101681611690

Na -0.02528665179055 5.49722551928823 5.47600323447036

Cl -0.12933844397309 2.79387502978315 5.62019916313034

Cl 0.01950801038356 5.71510860087600 2.72799754700644

Cl 2.68505215952013 5.56187259425743 5.55971888270864

Na 5.30690516076452 5.33238047195639 5.37884973585483

Cl 5.46876082419141 2.71119370795486 5.59035881706332

Cl 5.55902735298771 5.55069948929109 2.74085390960000

Na 0.02461122631730 5.60185296320553 -0.01309167159797

Na -5.61154654718488 0.08634777066483 -0.09857313406924

Na 5.48960597087280 -0.01689741019895 0.04871024578035

Na -0.06019812422406 -0.00434919853087 -5.49240714568161

Na -0.17928494304614 -0.05140709274795 5.63682690004242

Cl -0.03986229288376 2.84121332494925 0.00541499794313

Cl -2.89542045947527 0.09144878705518 0.11241160998275

Cl 2.72556372626059 -0.00167258746668 0.03626862382582

Na 2.77891663080740 0.01645850457477 2.87932192107613

Cl -0.07495280891177 0.01263258642559 -2.71981274033285

Cl 0.02170844675137 0.16864121978234 2.75714576271141

Na -2.87046430453560 -2.73645910202613 -5.41980203123193

Cl -2.86505756503630 -2.69901555011286 -2.62296033736571

Na -2.90103770936981 -5.45510445241919 -2.63800816761762

Na -5.62284766221608 -2.65275694482359 -2.68151940839447

Na -2.79732024671527 2.79945006446056 -5.51976371051582

Cl -2.82051269299064 2.81453809499218 -2.73847733138120

Na -2.74984483638406 5.60129850081289 -2.79569431963087

Na -5.58324938993795 2.87178210321630 -2.75537389231802

Na 2.66264399688559 -2.82085512687700 -5.45569555371561

Cl 2.64614357809099 -2.81314323254690 -2.67584172436182

Na 2.62412669756329 -5.57782435289195 -2.65677068318471

Na 5.43567342092168 -2.82099881538377 -2.70344788693626

Na 2.74754756626901 2.72472339789427 -5.51430675436510

Cl 2.74002169656092 2.74880908023933 -2.74839889310323

Na 2.78953212669939 5.52317432805461 -2.77098345869638

Na 5.51736915937153 2.71857206362048 -2.74112293559558

Na -3.01521901712713 2.87150159498987 5.57353586820888

Cl -2.87305778971958 2.90847394439956 2.74191124236837

Na -2.72398901788211 5.72858105598836 2.75001628001184

Na -5.58621008822384 3.33206115458567 2.82518117452545

Na 2.55351003326292 -2.93450111702681 5.63699902896072

Cl 2.69603329208142 -2.80395209402676 2.79216443956502

Na 3.03493848450919 -5.52727628205629 2.92257236006856

Na 5.50674902360177 -2.73533808469320 2.84677083410737

Na 2.65665368637255 2.75318159144246 5.57470213347103

Cl 2.73799856517104 2.80597498485909 2.78635512154443

Na 5.50327356598932 2.76937212313453 2.81488141943627

Na 2.77967116700294 5.56767770699406 2.78299366797721

Na -2.87911038872193 -2.67166641536062 0.24292199326250

Na -2.81841855952064 2.91294148621119 0.02228449620667

Na 2.72066757918481 -2.78451685876286 0.08370215048928

Na 2.77384152126047 2.80755561933467 0.03523845310097

Na -0.10539454320070 -2.76790779801123 -2.71611759217300

Na -0.02445985655567 2.83136668283601 -2.77478856748853

Na -2.85369898572622 0.06049756362647 -2.75111147349895

Na 2.74469949235885 -0.01895370345701 -2.74269082113487

Na -0.05925868138275 -2.49718989760696 2.89448235245517

Na -0.04488701625298 2.92171632159754 2.84229799905950

Na -2.63931731864868 0.14551887717579 2.85623377270739

H -3.31860109964963 -1.89270033888539 5.00414518753418

O -3.59569923717763 -2.81398297526589 4.71655630210950

C -3.21421141797364 -3.09279879343635 3.50952109655929

H -3.46509827684144 -4.12704615790003 3.22731427673939

O -2.64082907020585 -2.33116761251378 2.72126085515986

**Figure S16: TS_62_A**

Na -0.23945779996142 -0.24850903285096 0.26196140209695

Cl -0.27911659194286 -3.10136889310735 0.32100315625651

Na -0.35949294804964 -5.81795109719507 0.12637890273365

Cl -3.05297092142856 -5.83876495525667 0.32923573987750

Na -5.72149613332445 -5.63325907286280 0.19645795933606

Cl -5.85760543368080 -2.96473314270036 0.31173399562619

Cl -5.85800878939289 -5.78247823217195 -2.47010538097360

Na -5.65332463199023 -5.59049695646917 -5.10697111613196

Cl -3.02356136733216 -5.81098959849017 -5.27014721235173

Cl -5.81754624075787 -2.95687001521886 -5.27713798631125

Na -0.30020651970108 -5.76016536029032 -5.24603722322147

Cl -0.27583397016084 -3.04860340541663 -5.34895784555841

Cl -0.30159558609845 -5.92178990736997 -2.52834404600621

Na -5.70527253730575 -0.23731930005844 -5.25859658395946

Cl -2.99441569441294 -0.27431582410406 -5.34893041150439

Cl -5.88041442756628 -0.21848318029017 -2.53876877917322

Cl 2.41624778684017 -5.88707795183891 -5.27710286847023

Na 5.05055411969013 -5.71576205515136 -5.07353533063584

Cl 5.27494951069997 -3.08838379629096 -5.27644635200237

Cl 5.21280622635908 -5.91348244327524 -2.43303822612880

Na 5.19932101911719 -5.78609518144744 0.26793705902106

Cl 2.37039475768420 -5.98600043749840 0.38111044475036

Cl 5.33185169496920 -3.08385703670095 0.25020957709187

Na 5.22207844373293 -0.35935399169312 -5.20172849788058

Cl 2.50283332636361 -0.33924616644899 -5.34885090839157

Cl 5.37111791747183 -0.35200808193459 -2.49228444893949

Cl -5.77247979237239 2.47638338183498 -5.31503352624673

Na -5.54118370783319 5.10681249790036 -5.14566655788345

Cl -2.90707006095136 5.26921305450978 -5.33119554477185

Cl -5.74897656253967 5.29504811989153 -2.50889820769343

Na -5.63581986779735 5.30269944958364 0.18358593408464

Cl -2.92892792992213 5.36713196349029 0.18664396007642

Cl -5.90594936231784 2.48252378031054 0.35226497918877

Na -0.17621800476428 5.16368717468709 -5.24441715818808

Cl -0.22100773507684 2.44554861238399 -5.36625421413713

Cl -0.18273737103919 5.33558863691571 -2.53733775772709

Cl 2.54237509877615 5.21644672124357 -5.32554007086789

Na 5.17279456838550 4.99095331739421 -5.11290549939628

Cl 5.33659858332859 2.35474525485788 -5.30389898533615

Cl 5.37437721613591 5.20276302985587 -2.48549510577071

Na 5.28479011836645 5.12923291414754 0.24884602022215

Cl 2.57455831704534 5.31833110330916 0.22556388825541

Cl 5.40111270258015 2.41546091508449 0.24976230046764

Cl -0.39053654818564 -3.03527100973588 5.66305715025116

Cl -3.03815163711283 -0.06626102961520 5.86196990357944

Cl 2.67176610243147 -5.98087455955687 5.69010074415689

Na 5.23823289236051 -5.64892732634756 5.62495094293057

Cl 5.25795443875582 -3.01697023811600 5.80134212232858

Cl 5.53824721513393 -5.82182761277057 2.93463559594947

Na 5.13845560039405 -0.33567294985189 5.75277290429798

Cl 2.44036329154740 -0.33621181543038 5.86902114202229

Cl 5.37339192485005 -0.31369023459453 3.00822355024765

Cl -5.90446741751599 2.96687764789943 5.67297759979611

Na -5.52114365257086 5.52249051596825 5.52816676025245

Cl -2.87922039287644 5.47802950682244 5.71823738809131

Cl -5.67115253825255 5.71214543733901 2.83503266934798

Na -0.19724623845988 5.21039331671963 5.69637741833367

Cl -0.29833650117570 2.51014553506666 5.84990218493630

Cl -0.17097282714685 5.38941573558017 2.95445816759345

Cl 2.51293853775029 5.25416712495682 5.78597822752954

Na 5.13209655396549 5.00794082839579 5.60713274029527

Cl 5.27895556165824 2.38243326420717 5.81528609589086

Cl 5.37103669668627 5.22508543231273 2.97010823854685

Na -0.17091281163745 5.27597924581510 0.21832939242767

Na -5.80202490934745 -0.26706386803585 0.12164560223961

Na 5.29209988440747 -0.32673166339750 0.26782892913231

Na -0.24183413035735 -0.29667931432759 -5.27485035939744

Na -0.33517561181667 -0.34332522747788 5.87060428720670

Cl -0.23574481513583 2.51469496101455 0.24042089844839

Cl -3.09129041079299 -0.24916082865321 0.35885424034675

Cl 2.52541150096764 -0.31656319360628 0.25991045661717

Na 2.56748758260983 -0.30424080857719 3.09277850972957

Cl -0.26054705429316 -0.29518918259318 -2.49997659987585

Cl -0.21555003794844 -0.17588332702150 2.99850798912288

Na -3.03887889576379 -3.03299919096540 -5.23247728788172

Cl -3.03424869831155 -3.01773412136754 -2.43778549226356

Na -3.06257931831433 -5.77789484164430 -2.48956789526353

Na -5.79486486321820 -2.98402205080386 -2.49763653068759

Na -2.98682190173841 2.49481936058399 -5.28574255512710

Cl -3.01207382009064 2.49404146643346 -2.50202185363185

Na -2.94690762903863 5.27892922654243 -2.55852995010575

Na -5.77481153410384 2.54351222870948 -2.52362048985365

Na 2.49716085667245 -3.10364357978729 -5.26371716815000

Cl 2.47793416127091 -3.11225125921029 -2.48357492901001

Na 2.46202686930114 -5.87669199177069 -2.48675604321268

Na 5.26346851401961 -3.11317279531256 -2.49973283286226

Na 2.55827279246125 2.43744180105998 -5.28639024808539

Cl 2.54861730617678 2.44754420499041 -2.51980263993461

Na 2.59363066011911 5.22138192374688 -2.53560724762804

Na 5.32541423801642 2.42301174515360 -2.51343636351543

Na -3.18804486150592 2.65546168664564 5.82485693458155

Cl -3.07609270247500 2.57985922219456 2.99146678889304

Na -2.91494857227909 5.41028261048197 2.97148717877483

Na -5.78820881945936 3.02097825735864 3.06659469362550

Na 2.33225536853199 -3.25730920855770 5.83240320734415

Cl 2.50835595481801 -3.12865277376541 2.99542377827814

Na 2.84313391166342 -5.85641893712941 3.09619136147763

Na 5.31747221468132 -3.05735430043506 3.05613266530166

Na 2.47429413754548 2.44233647701009 5.80024232209255

Cl 2.53888755127987 2.47925482713359 3.01449797142384

Na 5.30624735831956 2.44556840205458 3.03858413836429

Na 2.59046946664077 5.24453537763486 3.01198638698258

Na -3.05603521828268 -3.03351730129818 0.44402693549486

Na -3.01632732260438 2.57138004836595 0.26366138629023

Na 2.53909659072710 -3.09890077465597 0.27516518638425

Na 2.57665995359514 2.49229604909229 0.26141052914444

Na -0.27748438112399 -3.07717769202016 -2.53203960837338

Na -0.21730537580386 2.51877199092569 -2.54349808044220

Na -3.04067434724988 -0.26163182383888 -2.52426444912360

Na 2.55471097261490 -0.32026984444853 -2.52474515556996

Na -0.22049801791347 -2.86439538582260 3.06787143279599

Na -0.24593155301183 2.58939923208528 3.07249172349507

Na -2.90633219932678 -0.19465318825034 3.14396765277091

H -3.85358441767345 -1.71243193074854 5.18590004829587

O -4.17972518402947 -2.39483735695603 4.50376979852768

C -3.22447436424961 -3.12288008501661 3.95200003775478

H -2.99996491679872 -4.08013744771581 4.44619685031904

O -2.66349163280654 -2.78628743033877 2.91176344650807

**Figure S16: IM_62_B**

Na -0.25349492775076 -0.29195701623404 0.28632958103706

Cl -0.28134918050719 -3.13786618975425 0.33283974232398

Na -0.37328060935848 -5.85489935370459 0.10671019566811

Cl -3.06619757061077 -5.93168320872120 0.23185280812462

Na -5.73073196881281 -5.69951321248618 0.14038963667348

Cl -5.88727440300906 -3.02360303505310 0.27416430607913

Cl -5.86617187582561 -5.80701874720170 -2.53434638277604

Na -5.64632658140809 -5.58917115264359 -5.16193440727215

Cl -3.01359056092701 -5.80415728491894 -5.31864170250923

Cl -5.81384545406456 -2.95266705070384 -5.29769461372285

Na -0.28982742697111 -5.75565087173510 -5.26996187847855

Cl -0.27318406676732 -3.04423702797471 -5.34612492854939

Cl -0.29169013717202 -5.93293468240036 -2.55537135851632

Na -5.70643166145410 -0.24015365801582 -5.24201217471393

Cl -2.99706326714686 -0.27323728729844 -5.32658916144592

Cl -5.87995826818761 -0.23393131154914 -2.51796907262451

Cl 2.42587528334137 -5.88108909602752 -5.29450301260523

Na 5.05770166019643 -5.70750073900594 -5.08775378585360

Cl 5.27620335193965 -3.07867661147338 -5.28068898988524

Cl 5.21734675158719 -5.91425409357493 -2.44822450887878

Na 5.20116713859416 -5.79258289564136 0.25111287061269

Cl 2.37310752611340 -6.00402057283601 0.37661008407882

Cl 5.33142280605075 -3.08945637096588 0.24134585503691

Na 5.21549906015167 -0.35269575506115 -5.19631861471712

Cl 2.49653074880565 -0.33564045574463 -5.33701087735956

Cl 5.36241019706738 -0.35203556171205 -2.48719646293071

Cl -5.78112417738513 2.47056490893325 -5.29026856001050

Na -5.55228431874403 5.09942371986817 -5.12165794129609

Cl -2.91810048538084 5.26394336395100 -5.30805700013232

Cl -5.75801775275190 5.27786444237864 -2.48385491680599

Na -5.63386634804960 5.29925046523666 0.20310389028594

Cl -2.92993466629595 5.37394181839047 0.20074053134873

Cl -5.89896523574828 2.46000189135326 0.38533548231711

Na -0.18667504653875 5.15858753101310 -5.22329285261730

Cl -0.22998191204585 2.44261947201450 -5.34506116686205

Cl -0.19064204961918 5.31802708521795 -2.51576836449256

Cl 2.53059001843289 5.21677031545024 -5.30830087996170

Na 5.16192502480163 4.99551616176927 -5.10095036934121

Cl 5.32758842520342 2.36057149160565 -5.29493772872095

Cl 5.36502100350102 5.20069899483963 -2.47328609825347

Na 5.27856903955855 5.11934799104006 0.25867858061037

Cl 2.56781348427647 5.29838842533938 0.24059089460963

Cl 5.39292821752103 2.40675222378599 0.25712762957224

Cl -0.27436723898966 -3.04615209551906 5.72844537360114

Cl -3.10118235771536 -0.05528224800963 5.86207315512769

Cl 2.74261882357148 -5.99619537664575 5.69337663865186

Na 5.30411956909331 -5.65583933409383 5.60412130618286

Cl 5.31152334236485 -3.02030027101645 5.79061278593084

Cl 5.57230084540638 -5.82853407236289 2.91255039121174

Na 5.14724399056912 -0.33866237254630 5.74971515584852

Cl 2.44996978313632 -0.33803426185011 5.87897501178104

Cl 5.36524099828648 -0.32160745005006 3.00736649080845

Cl -5.91125742836021 2.98459652737550 5.67954762218485

Na -5.51279865111379 5.54049272664522 5.54439623000427

Cl -2.87309782861511 5.49808516004255 5.74597621280182

Cl -5.67008571850284 5.75407624494094 2.85014677403136

Na -0.19348522210727 5.19753671574594 5.71020443996831

Cl -0.29922524448702 2.49604841270426 5.85685684002191

Cl -0.16223712982688 5.39083851160134 2.97055134284796

Cl 2.51670939383528 5.23731054386332 5.80025350503025

Na 5.13708681034153 5.00025911169578 5.61647596182546

Cl 5.28843307519448 2.37679161022616 5.81940824927070

Cl 5.36768000432649 5.21350517510466 2.97845823568815

Na -0.17475045109157 5.25230716062323 0.23715226266698

Na -5.80497643134948 -0.32015039216524 0.14686403294866

Na 5.27991141585865 -0.33537046275969 0.27096538773898

Na -0.24697774169349 -0.29854818690417 -5.25548303411886

Na -0.36619699600147 -0.35875098394242 5.90465773767527

Cl -0.24746251001238 2.48464064673247 0.26773038441860

Cl -3.10142744070128 -0.28497798783974 0.42342195142661

Cl 2.51083443887082 -0.33289013432739 0.27582519859862

Na 2.54997659798886 -0.33202704293441 3.10886850381627

Cl -0.26946610125077 -0.30932405866423 -2.47797207099472

Cl -0.26213074147835 -0.24911592991127 3.02835861519390

Na -3.03474443295430 -3.02986715435675 -5.23498189124623

Cl -3.03799704986000 -3.03530207754285 -2.43814953807546

Na -3.06067158472288 -5.80514668090091 -2.54547177082975

Na -5.80020582360669 -3.00821338676289 -2.52081143946443

Na -2.99478508747384 2.48949470361773 -5.26248618026696

Cl -3.01921165774657 2.47962811178786 -2.47633658344887

Na -2.95400048091029 5.26888204486842 -2.53931976571303

Na -5.78208744058888 2.52549096223933 -2.49858663416613

Na 2.49761034469207 -3.09988145587036 -5.26274129628390

Cl 2.47732721015187 -3.11830437489718 -2.48257278041052

Na 2.46583922862379 -5.88341118326040 -2.49906026915337

Na 5.26208604096855 -3.11391365437807 -2.50505822311456

Na 2.54900011699398 2.43757813882345 -5.27217290200306

Cl 2.53989772405922 2.43890915172815 -2.50506453753714

Na 2.58489724487101 5.21241484560523 -2.51952200514295

Na 5.31732443442925 2.42051574009449 -2.50504838558526

Na -3.19025894939894 2.66041245989402 5.86982029092839

Cl -3.06688843175078 2.69344853818691 3.00811932888259

Na -2.90374335252638 5.48684972151420 2.99027683624813

Na -5.79335442037405 3.06736610718324 3.07784021172219

Na 2.41911517035261 -3.26620829883548 5.85015817144659

Cl 2.53400250890349 -3.15301631473419 2.99911437052140

Na 2.87575863874881 -5.88132181957205 3.09518488349487

Na 5.34024702728326 -3.06479317306689 3.04263310857827

Na 2.48100721178837 2.42786691392228 5.81447879577707

Cl 2.53106817067151 2.45697252453144 3.02628821102396

Na 5.30280567166988 2.43332521471213 3.04420308882326

Na 2.59200453598695 5.22919633733639 3.02584549529555

Na -3.07791551423490 -3.11978879870663 0.45888552428829

Na -3.02423803529604 2.56950399756807 0.27863543311928

Na 2.53406019975748 -3.11868111400089 0.27789355500466

Na 2.57013021762419 2.47348231001696 0.27348988691412

Na -0.28126218418158 -3.09516822127548 -2.52440323695834

Na -0.22443955866433 2.50185732364086 -2.52217634406486

Na -3.04669794890745 -0.28126618259878 -2.49637841167080

Na 2.54621325772760 -0.32949262304416 -2.51229407946690

Na -0.19529201633696 -2.98616151209146 3.10838090896450

Na -0.23385740176009 2.57624978162006 3.08856871420071

Na -2.91596360479233 0.03725375076334 3.17253884087588

H -4.25237744482352 -1.66415927988251 4.77656510887145

O -4.15298358858163 -2.04786152200637 3.86933631664618

C -3.14747839232372 -2.92502715542514 3.88896389450356

H -2.80440494170150 -3.24659516101202 4.88498979371990

O -2.62974525194311 -3.29948248094119 2.85136953551664

**Figure S16: TS_62_B**

Na -0.25298522818281 -0.28451830911436 0.15531364035019

Cl -0.44168544851203 -3.11281957905592 0.34624255375057

Na -0.47379433249614 -5.82424202930462 0.07372964951207

Cl -3.18082164163147 -5.83178688479910 0.15656878816914

Na -5.84259480335734 -5.62714159533290 -0.02530885505658

Cl -5.98970707310704 -2.94977172907856 0.05820962416579

Cl -5.91281466722489 -5.76246748876704 -2.70477473873646

Na -5.62871773174611 -5.56894559862422 -5.33375236007964

Cl -2.99785693927099 -5.79843820934402 -5.41911385700055

Cl -5.77499594683930 -2.93176055783679 -5.51870623716469

Na -0.28185674556841 -5.75025920776490 -5.30884234021010

Cl -0.24605029622165 -3.03892803696417 -5.41126171291640

Cl -0.35008418031928 -5.91171430014337 -2.58944926677580

Na -5.65311851486259 -0.20825197925780 -5.50396901258039

Cl -2.94073981264887 -0.24986939780286 -5.52483371479246

Cl -5.89691323897793 -0.20017000239180 -2.79296542387799

Cl 2.42573025395368 -5.88805100181873 -5.29205913609866

Na 5.05540586679928 -5.72551923945269 -5.05434019680479

Cl 5.28724716989369 -3.09614388083182 -5.24088721104898

Cl 5.16101607026879 -5.92920587611941 -2.41583278021372

Na 5.09280612138682 -5.80794750516448 0.27469348167957

Cl 2.26071865353248 -5.99878859396203 0.36759344158182

Cl 5.22970142197220 -3.10474814092602 0.27081089206347

Na 5.26683037508290 -0.36167312424197 -5.17664906389121

Cl 2.55123406938944 -0.33392014939692 -5.37206817110797

Cl 5.37165314650984 -0.36232214471832 -2.46847134670774

Cl -5.71103519091644 2.51644344392309 -5.52805889085412

Na -5.48593003144092 5.14358606364798 -5.29665695433089

Cl -2.84967215275119 5.30691168151839 -5.42793596258649

Cl -5.75547386294482 5.29958325829492 -2.65607885915497

Na -5.71197348313266 5.26798886025222 0.05306749181968

Cl -3.00773359895020 5.36575398434727 0.10839740482796

Cl -5.95279668738172 2.44896674488657 0.12652805978625

Na -0.12320942561201 5.18258139652479 -5.28159054151864

Cl -0.16448094716872 2.46364866990773 -5.44004541644459

Cl -0.19303884771427 5.33769787659046 -2.57118286318774

Cl 2.59336479503542 5.22900208088408 -5.30779965767091

Na 5.21872418618034 4.99754414251592 -5.04481294733258

Cl 5.38599248899769 2.36156872741935 -5.25586586174065

Cl 5.36103190069541 5.20011916255409 -2.41219572776714

Na 5.21420625477131 5.11751217388134 0.32324125103632

Cl 2.50191865087075 5.30127671080275 0.24782202468807

Cl 5.35578058951394 2.40597026472633 0.29697488436824

Cl -0.32304401872420 -3.17046909540530 5.79324316955882

Cl -1.21056257563535 -1.16708456896995 8.45459637238821

Cl 2.65303181590676 -6.02767638014910 5.71428826781491

Na 5.20943118993564 -5.69416237276166 5.61351642064479

Cl 5.21794123247875 -3.05237910672776 5.79647079926768

Cl 5.43230288426491 -5.85325835083567 2.92475698169923

Na 5.03096130484227 -0.37217046109861 5.76872161574805

Cl 2.33163709263377 -0.39692928337573 5.82381506768726

Cl 5.31234177115766 -0.34097364241623 3.02494569757431

Cl -5.97553150332386 2.50805078897726 5.41630470039374

Na -5.72286745543722 5.11138225874244 5.43012798842414

Cl -3.10187937017456 5.19920552522935 5.66249398927214

Cl -5.83066042811004 5.52483265239192 2.75090816026846

Na -0.41669268552000 5.05602778825800 5.67799502844748

Cl -0.52107196047242 2.33846560062901 5.74660459957640

Cl -0.30580175833226 5.34953406903105 2.93850083466064

Cl 2.28820539469274 5.13550053309310 5.81886995826786

Na 4.92495102340732 4.95697303171240 5.68103753225596

Cl 5.13543188795968 2.34134522117618 5.85666151445167

Cl 5.24013792170218 5.21773234441302 3.05240498441393

Na -0.23937039109294 5.26140965112187 0.18704808572437

Na -5.86330509176220 -0.27834111818744 -0.13888666848473

Na 5.25406758259203 -0.34752345617914 0.28719968767553

Na -0.18989318961905 -0.28807868045438 -5.36399558350254

Na -0.52388945293996 -0.42430989543584 5.70307912771719

Cl -0.29177392502252 2.49818201600248 0.16033027728403

Cl -3.14357946443286 -0.25532026657182 0.10560178571047

Cl 2.49742525664096 -0.33446377687530 0.21701386526697

Na 2.53218629044896 -0.35594189252284 3.06175712923903

Cl -0.26545369844108 -0.29697860572894 -2.59280982125491

Cl -0.16233813284208 -0.18048436593077 2.87075170887664

Na -3.00318632326109 -3.01971602263125 -5.38052731327294

Cl -3.07798538996800 -3.00554863877091 -2.58736978307190

Na -3.11322959030532 -5.76594191881642 -2.64204028263579

Na -5.83343106553442 -2.96945522780239 -2.74211669073722

Na -2.92763241712425 2.52668792027730 -5.43908404596224

Cl -3.01294436903908 2.50948492356071 -2.65997136927134

Na -2.95362505867976 5.29390078461961 -2.65613487435142

Na -5.77586532193210 2.55096734787186 -2.74396346327669

Na 2.51552956082672 -3.10500003369987 -5.26498006935789

Cl 2.42416591223513 -3.11876052239601 -2.47976295807792

Na 2.41176504605178 -5.88464541090401 -2.49675145360629

Na 5.21477418274377 -3.12831189877467 -2.46864632912696

Na 2.61218891232483 2.44647653434368 -5.29400888336624

Cl 2.54537228343106 2.44407525441878 -2.52812434331389

Na 2.58726991096546 5.21786350032810 -2.51736481736364

Na 5.32306110471885 2.41607804111113 -2.46600445332106

Na -3.28652264891039 2.30086331324466 5.60527425889254

Cl -3.14172992565606 2.55214869371896 2.82301109402248

Na -3.05539876711596 5.35560788381905 2.91220432043258

Na -5.87574235095731 2.83973092741559 2.82364374397719

Na 2.36264193521253 -3.29593311053482 5.84527524373556

Cl 2.34947015518531 -3.16765588306238 3.00443852205050

Na 2.72484144408621 -5.88031526863936 3.11124832601512

Na 5.18586371492038 -3.09389312345146 3.05715529155438

Na 2.31692127231884 2.33361749527926 5.79206126446841

Cl 2.46624324859799 2.45939404312433 3.00642801297435

Na 5.22670443628604 2.42593925730958 3.08683902824452

Na 2.46526992873056 5.21251191623472 3.03932671196524

Na -3.21615218717015 -3.03976095723384 0.34115828581620

Na -3.06383524100046 2.57480364041934 0.08223227769221

Na 2.42283386084667 -3.12118654328912 0.29521170322659

Na 2.53131119228082 2.46933786936930 0.25441107654977

Na -0.32875332451962 -3.08111173667213 -2.57511283413519

Na -0.21492386284399 2.51741722828224 -2.62133936865891

Na -3.04656241145411 -0.25467269267281 -2.72017243235234

Na 2.55724704252274 -0.32838662076050 -2.55256375384122

Na -0.54786016530083 -2.86591973929955 3.16201065464240

Na -0.29255892451627 2.55924787581903 2.97336645896951

Na -2.80533983990858 -0.08488175378519 2.75347532459968

H -0.97774968074397 -2.19932463608530 7.68051802671783

O -2.86188741877507 -0.70539111151493 4.96526810361951

C -3.04916634144488 -1.89715220580226 4.57876027397464

H -3.39408734689010 -2.63456313858963 5.32847802190008

O -2.87292390388917 -2.30747309699243 3.38720613584564

**Figure S16: IM_62_C**

Na 0.00684450490338 0.09109127837517 0.05190181737935

Cl -0.24363473528665 -2.73431475900075 0.33550376789313

Na -0.26578559397512 -5.44346781311203 0.04346054080091

Cl -2.98024041628151 -5.46268900584418 0.11653970166685

Na -5.64428296620540 -5.25058522713409 -0.00673090024249

Cl -5.75660936625213 -2.57397190097016 0.03899136912213

Cl -5.75973520242022 -5.41002917165983 -2.68308070573357

Na -5.53091699728975 -5.24660231624527 -5.32055316568889

Cl -2.90143200055522 -5.49904797833449 -5.45034418512939

Cl -5.66872338304030 -2.60943303017260 -5.52876665154234

Na -0.18448683451747 -5.46560565663303 -5.35692579662471

Cl -0.13864774583864 -2.75830498002193 -5.50568907860705

Cl -0.20082438593982 -5.57347740911201 -2.62696821848603

Na -5.53395666584500 0.11707956574389 -5.51130381231075

Cl -2.82422655041821 0.05256146385754 -5.59842422978665

Cl -5.69957818976441 0.14793769948098 -2.79185970523454

Cl 2.51555709962076 -5.62876508920166 -5.37779079346871

Na 5.15194156115672 -5.48475973700110 -5.20703088443091

Cl 5.38825524478108 -2.86180832118837 -5.45342740128114

Cl 5.31024742049946 -5.63452750644128 -2.56610340146325

Na 5.30185572284355 -5.45183435476723 0.12255518529774

Cl 2.47077648743254 -5.61518554533469 0.29716060812524

Cl 5.44804976624342 -2.75248831647482 0.05269836393821

Na 5.38188967190289 -0.12534868638904 -5.45821710719428

Cl 2.66198710262028 -0.08155602832142 -5.57276651032540

Cl 5.55748680680509 -0.07157183542240 -2.75745027502222

Cl -5.58467769107305 2.83629731687120 -5.55167288817675

Na -5.34372885384409 5.46640820393081 -5.35325959828416

Cl -2.71192744228069 5.60459463649315 -5.57263746600634

Cl -5.53356598317543 5.65021034193035 -2.70828373045438

Na -5.40739363657356 5.65321454948020 0.01010378763275

Cl -2.70508631428670 5.74929504791202 -0.03985335970456

Cl -5.65769103204632 2.82955172530060 0.10513557682446

Na 0.01623803048660 5.46096481427007 -5.51641445129237

Cl -0.04067000396807 2.73778046925301 -5.61822798573621

Cl 0.02943877335635 5.65999288188738 -2.81068423401908

Cl 2.73065289969228 5.48128641178913 -5.63987910199481

Na 5.36251648248596 5.23658008664252 -5.46160544236692

Cl 5.51023375211141 2.59343028770531 -5.61257337195422

Cl 5.58697557926589 5.49397674724674 -2.84282888325369

Na 5.52302443480280 5.47276284860618 -0.11037004895056

Cl 2.81006527085103 5.66911050721267 -0.09533190345457

Cl 5.64699870903241 2.75740360003594 -0.07401105562305

Cl 0.14757024985679 -2.53088559464739 5.87813624071244

Cl -0.62937256004741 -0.38852779710072 8.45912338835330

Cl 3.04039600820010 -5.49150210759478 5.64537098929355

Na 5.59800128397983 -5.20322324717318 5.44901602145429

Cl 5.66773984084100 -2.55730832948577 5.56008816359659

Cl 5.71723813525228 -5.43649167383275 2.75974208907249

Na 5.54373104161399 0.13420124205609 5.46499520679014

Cl 2.85871959861760 0.14763186054362 5.62673007626227

Cl 5.67561112307039 0.07932031471215 2.71784520777232

Cl -5.46807415407807 2.87647558510451 5.38193716030773

Na -5.20021015027794 5.47747381653851 5.39062276505437

Cl -2.59538630457206 5.65630895441429 5.51934855280592

Cl -5.44528186159807 5.92785777678919 2.70832168978906

Na 0.08499960877152 5.59630868449447 5.44192232237702

Cl 0.04044532846453 2.90389058531218 5.57402255952556

Cl 0.09964516715836 5.80689050369570 2.68740357957243

Cl 2.81558868551104 5.68136751318571 5.47185870628584

Na 5.44206091842630 5.47105951109360 5.24447949343499

Cl 5.65917774506111 2.85680250429959 5.47860245013580

Cl 5.64963922344166 5.64452644030141 2.60051092457309

Na 0.06316556368511 5.63848751933859 -0.04992231201692

Na -5.61455753932660 0.10509895060817 -0.13298166525123

Na 5.51699863464405 0.00571711987525 -0.00529701055474

Na -0.07295813427337 -0.00658925950990 -5.48849722853712

Na 0.06141637073449 0.20052761728325 5.65237005485672

Cl 0.00044098728710 2.86925851153657 -0.02190738913235

Cl -2.88724177539333 0.09356768937692 0.08615566862469

Cl 2.75601147058819 0.03178174734295 0.01516797031112

Na 2.88962065352753 0.08909735838028 2.85905341858116

Cl -0.08425876614049 0.02134265842155 -2.70845428160445

Cl 0.18313679686120 0.25308610579469 2.76444880773252

Na -2.89260543920307 -2.71508854617863 -5.47385990271038

Cl -2.90769660896072 -2.67142452793985 -2.68759877485572

Na -2.96270626667029 -5.44792996090271 -2.67879404275335

Na -5.67984290718481 -2.61885351429424 -2.75831950421149

Na -2.80072899342080 2.83113733352529 -5.54206145493219

Cl -2.80549408963957 2.84342682748510 -2.75823468686176

Na -2.73293474684321 5.63519835805999 -2.79501597751283

Na -5.56820069198087 2.90574962280775 -2.76521398642983

Na 2.62113898602535 -2.84731780694140 -5.40499806642052

Cl 2.58687205291622 -2.80492079272268 -2.61236343250874

Na 2.56456694933254 -5.56789232099923 -2.58279097287486

Na 5.37928421832418 -2.83476001771654 -2.68012674645184

Na 2.73751243268548 2.70094695314368 -5.56373966295021

Cl 2.75235426148386 2.75158119088143 -2.79979682123456

Na 2.80908657817195 5.52740450317621 -2.85240201218143

Na 5.53085203854104 2.71137597022096 -2.82641987113476

Na -2.75757857458588 2.53304580200148 5.51500501044988

Cl -2.74098717336394 3.00095711829069 2.70508821433025

Na -2.65710282815996 5.78738595317644 2.78399405921596

Na -5.46710305653953 3.25158070359091 2.79861002180500

Na 2.82151249063516 -2.74877385727697 5.73405045971516

Cl 2.64632167685107 -2.71809445515606 2.89382954832824

Na 3.02057696646940 -5.42438689793955 3.03534487686001

Na 5.49689723051518 -2.66872884675612 2.83169602936789

Na 2.84455266359674 2.88509196535522 5.51900686076939

Cl 2.85748119264954 2.89795531601336 2.72640852759550

Na 5.61758897611966 2.85403874829493 2.71140852506398

Na 2.87017168872892 5.65816152576703 2.69500530639246

Na -2.98881298510397 -2.68553956597857 0.08396880257891

Na -2.77311894791384 2.95366398220013 -0.01452702342857

Na 2.63689536817493 -2.74550466301240 0.17830873082401

Na 2.82215662667383 2.84110290422875 -0.01923725411434

Na -0.15415414862439 -2.75146097745198 -2.66538254996815

Na -0.00858494066605 2.84398539572417 -2.79957009106303

Na -2.85965345136617 0.09025544727312 -2.78696316905861

Na 2.74395604728594 -0.01617044532274 -2.75549066692053

Na -0.30261739699234 -2.38088825433111 3.25837538692889

Na 0.09439742953951 3.01353088976494 2.79987594098065

Na -2.46572473567765 0.36871089468747 2.74528562199102

H -0.41435593919131 -1.46394878694406 7.74106757557297

O -2.38035667357454 0.18832457129125 5.09642889271580

C -2.61880794997137 -1.05041998594666 4.92726801004458

H -2.91415581902393 -1.64008800202815 5.81594729057054

O -2.54082381187659 -1.64868611964789 3.81662998346151

**Figure S16: Na_62_Cl_60_(HCOO)^+^**

Na 0.00606868053108 0.08633583957724 0.04837442708039

Cl -0.24365634308088 -2.73467139301629 0.33402039383233

Na -0.26403313952043 -5.44240403081012 0.04434866872326

Cl -2.97971183898277 -5.46306971048032 0.11478992635272

Na -5.64239221547450 -5.25113740415761 -0.00742948854501

Cl -5.75690972385953 -2.57437508974367 0.03699381406714

Cl -5.75920611722917 -5.41057802182584 -2.68437267032310

Na -5.52888111019650 -5.24696170299989 -5.32113254540395

Cl -2.89979309346454 -5.49880972009253 -5.45125355454516

Cl -5.66780712923951 -2.60969493053095 -5.52999308122186

Na -0.18251380301437 -5.46488774434100 -5.35638202536646

Cl -0.13764993962300 -2.75755597990679 -5.50554171420521

Cl -0.19979777959714 -5.57339643673907 -2.62768835605656

Na -5.53294786491303 0.11624169676804 -5.51280356432056

Cl -2.82375464944109 0.05298637801480 -5.59941334524356

Cl -5.70001573605943 0.14840009042930 -2.79352352583782

Cl 2.51741576112054 -5.62749392677334 -5.37931751698044

Na 5.15359252139309 -5.48231936202024 -5.20707699792732

Cl 5.38969144373007 -2.85935689530116 -5.45415547583582

Cl 5.31298138792541 -5.63268401766880 -2.56721007361330

Na 5.30381460113068 -5.44973330922290 0.12305613931594

Cl 2.47279031670584 -5.61620177399525 0.29073532022354

Cl 5.45099488119990 -2.75079497173547 0.05182414835733

Na 5.38270158418337 -0.12357289049245 -5.45809347933802

Cl 2.66278185473953 -0.07961823312346 -5.57380795084044

Cl 5.55838186483129 -0.06897720570250 -2.75781473048846

Cl -5.58465553694679 2.83620623982645 -5.55459204814179

Na -5.34337368285115 5.46588144609812 -5.35607172996093

Cl -2.71180005012057 5.60523003859581 -5.57432708992081

Cl -5.53377675571896 5.65053759770610 -2.71083370071066

Na -5.40754244863217 5.65256176647762 0.00624723857965

Cl -2.70550428163989 5.74908776339057 -0.04016549933935

Cl -5.65913188651529 2.83061608719751 0.10214379179783

Na 0.01627400921382 5.46120659476730 -5.51641926574587

Cl -0.04036600381600 2.73870319453551 -5.61930105009531

Cl 0.02840686764725 5.66028691746725 -2.81018948403152

Cl 2.73018679711114 5.48347491991354 -5.63976792121919

Na 5.36191903469758 5.23840502754530 -5.46093790682394

Cl 5.51073258129245 2.59591038881433 -5.61280532609379

Cl 5.58566480753620 5.49646470419728 -2.84205206517904

Na 5.52107648544645 5.47323748171592 -0.11034386569332

Cl 2.80764731368636 5.66991585166704 -0.09413341821641

Cl 5.64630554312245 2.75918598036317 -0.07368910944766

Cl 0.12890068800049 -2.46168213479959 5.88336289857736

Cl 3.02571592060753 -5.51089325006391 5.63501872328237

Na 5.57928055638455 -5.20239204069955 5.44947670472304

Cl 5.64202904939271 -2.55902007659750 5.56672080041981

Cl 5.71779946628835 -5.43649536658480 2.75963568354702

Na 5.54446251811772 0.12987502189764 5.46472901236452

Cl 2.86495989635167 0.16794810017849 5.63271061045660

Cl 5.67649185744123 0.08072955370502 2.71850713762772

Cl -5.47508277780766 2.87715568931791 5.37735142045441

Na -5.20531747647774 5.47807392592233 5.38586573891333

Cl -2.59711041405164 5.63733666221272 5.52036129467735

Cl -5.44501923203222 5.92936760023625 2.70654683751318

Na 0.08428057404695 5.57726538104861 5.44475800353903

Cl 0.03729427657642 2.87789023536166 5.59823870765220

Cl 0.09732271868324 5.79534008773708 2.69379884048379

Cl 2.81272592288524 5.67653047228649 5.47475647145837

Na 5.44288416732809 5.47209896864093 5.24359154021096

Cl 5.66790949093701 2.85980512552987 5.47793222282498

Cl 5.64622383449478 5.64667849603948 2.60074866241821

Na 0.06244418957994 5.63420039226511 -0.04898155401220

Na -5.61398995120777 0.10370806373260 -0.13457085579988

Na 5.51723130575588 0.00499591789679 -0.00432198289017

Na -0.07213897493263 -0.00665305670783 -5.48956968740804

Na 0.06988216810787 0.15948334452055 5.61567924324107

Cl -0.00051273885217 2.86840443624919 -0.02234392501666

Cl -2.88873321669993 0.09570671712125 0.08752999155222

Cl 2.75667738295007 0.03377840926797 0.01351369640507

Na 2.88867532513690 0.08321178308902 2.86237084776682

Cl -0.08444200617479 0.02231705542816 -2.71048938799521

Cl 0.17948523746195 0.25105732761930 2.75395783069432

Na -2.89104031801660 -2.71526603861707 -5.47324015721735

Cl -2.90763420371105 -2.67070811146162 -2.68752830708435

Na -2.96097370827867 -5.44765736371007 -2.67899416173186

Na -5.67873868355663 -2.61957911397285 -2.75930538223318

Na -2.80017179482280 2.83078218488361 -5.54455602276035

Cl -2.80612870774173 2.84410778937997 -2.76078694435184

Na -2.73301847481085 5.63486185725145 -2.79696675129329

Na -5.56867009865831 2.90512030293771 -2.76870435424164

Na 2.62231444179996 -2.84615469594130 -5.40510039733335

Cl 2.58816515194760 -2.80336812459186 -2.61417629203639

Na 2.56652004026999 -5.56718548014846 -2.58329929145516

Na 5.38055397712664 -2.83331474944606 -2.67950943395249

Na 2.73794037502981 2.70210364807517 -5.56448999753259

Cl 2.75219042593765 2.75348569501081 -2.80067285235217

Na 2.80789163334690 5.52800292057807 -2.85218395795692

Na 5.53086245902467 2.71249660359305 -2.82627614145035

Na -2.76435938821566 2.54600037758511 5.48877076210714

Cl -2.74297731492637 3.00251741259121 2.70108469702978

Na -2.65795903683847 5.78681843341743 2.78083183041351

Na -5.47177744545430 3.25184131666718 2.79271572131935

Na 2.76467452568247 -2.76585380596880 5.73851075895102

Cl 2.65381697254927 -2.71799525951977 2.88637429084963

Na 3.01833491672594 -5.42416599047340 3.02876180275966

Na 5.48890049166968 -2.66974359650434 2.83695375726718

Na 2.85523841696112 2.88664611797102 5.51063814448418

Cl 2.85604378759997 2.89803374207999 2.72602782127833

Na 5.61884189899309 2.85289478908641 2.71106431319152

Na 2.86885986120748 5.65656674144213 2.69431174855698

Na -2.98711256242382 -2.68535548194181 0.08633360736199

Na -2.77259672261803 2.95083994194428 -0.01899751664869

Na 2.63763719556178 -2.74522295545970 0.17707533835141

Na 2.82142400058620 2.83910068032667 -0.02022043052733

Na -0.15341842852813 -2.75195488787606 -2.66262166166837

Na -0.00851483730181 2.84329840399578 -2.80058046924622

Na -2.85950293274539 0.08967252851504 -2.78710616000363

Na 2.74487392732312 -0.01560612347917 -2.75594752320706

Na -0.30646849787211 -2.39256664999479 3.28895443923649

Na 0.09524078880491 3.00263153851016 2.80233868279160

Na -2.46799529593323 0.37083346718112 2.75254893266952

O -2.37592400325422 0.19380166863555 5.12274059668578

C -2.62392306624180 -1.04417386446826 4.94306762651516

H -2.91001229142165 -1.64175332805852 5.82824120886116

O -2.56519659414712 -1.62916259049701 3.82525334861617

**References**

[1] A. Herburger, C. van der Linde, M. K. Beyer, *Phys. Chem. Chem. Phys.* **2017**, *19*, 10786–10795.

[2] R. F. Höckendorf, C. van der Linde, O. P. Balaj, I. Herber, M. K. Beyer, *Int. J. Mass Spectrom.* **2011**, *300*, 44–49.

[3] J. E. Bartmess, R. M. Georgiadis, *Vacuum* **1983**, *33*, 149–153.

[4] C. van der Linde, *Dissertation*, Christian-Albrechts-Universität zu Kiel, Kiel, **2012**.

[5] A. Baloglou, M. Pritzi, T. F. Pascher, J. C. Hartmann, M.-L. Grutza, M. Ončák, P. Kurz, M. K. Beyer, *Int. J. Mass Spectrom.* **2021**, *464*, 116558.

[6] C. G. Gray, K. E. Gubbins, Theory of Molecular Fulids. Oxford University Press, 1984.

[7] G. Kummerlöwe, M. K. Beyer, *Int. J. Mass Spectrom.* **2005**, *244*, 84–90.

[8] T. Su, M. T. Bowers, *Int. J. Mass Spectrom. Ion Process.* **1975**, *17*, 211–212.

[9] F. Neese, *WIREs Comput. Mol. Sci.* **2012**, *2*, 73–78.

[10] F. Neese, *WIREs Comput. Mol. Sci.* **2022**, *12*.

[11] B. de Souza, *Angew. Chem. Int. Ed.* **2025**, *64*, e202500393.

[12] S. Grimme, C. Bannwarth, P. Shushkov, *J. Chem. Theory Comput.* **2017**, *13*, 1989–2009.

[13] S. Grimme, A. Hansen, S. Ehlert, J.-M. Mewes, *J. Chem. Phys.* **2021**, *154*, 64103.

[14] K. A. Peterson, T. B. Adler, H.-J. Werner, *J. Chem. Phys.* **2008**, *128*, 84102.

[15] F. Neese, *J. Comput. Chem.* **2003**, *24*, 1740–1747.

[16] S. Kossmann, F. Neese, *J. Chem. Theory Comput.* **2010**, *6*, 2325–2338.

[17] T. D. Kühne, M. Iannuzzi, M. Del Ben, V. V. Rybkin, P. Seewald, F. Stein, T. Laino, R. Z. Khaliullin, O. Schütt, F. Schiffmann et al., *J. Chem. Phys.* **2020**, *152*, 194103.

[18] J. P. Perdew, K. Burke, M. Ernzerhof, *Phys. Rev. Lett.* **1996**, *77*, 3865–3868.

[19] G. J. Martyna, M. E. Tuckerman, *J. Chem. Phys.* **1999**, *110*, 2810.

[20] S. Goedecker, M. Teter, J. Hutter, *Phys. Rev. B* **1996**, *54*, 1703–1710.

[21] S. Grimme, J. Antony, S. Ehrlich, H. Krieg, *J. Chem. Phys.* **2010**, *132*, 154104.

[22] G. Bussi, D. Donadio, M. Parrinello, *J. Chem. Phys.* **2007**, *126*, 14101.

[23] G. A. Tribello, M. Bonomi, D. Branduardi, C. Camilloni, G. Bussi, *Comput. Phys. Commun.* **2014**, *185*, 604–613.

[24] M. Invernizzi, M. Parrinello, *J. Phys. Chem. Lett.* **2020**, *11*, 2731–2736.

[25] A. Barducci, G. Bussi, M. Parrinello, *Phys. Rev. Lett.* **2008**, *100*, 20603.

[26] H. Fu, H. Chen, X. Wang, H. Chai, X. Shao, W. Cai, C. Chipot, *J. Chem. Inf. Model.* **2020**, *60*, 5366–5374.

[27] P. J. Linstrom, W. G. Mallard (Eds.) *NIST Chemistry WebBook, NIST Standard Reference Database Number 69*, National Institute of Standards and Technology, Gaithersburg MD, 20899, **2005**.
